# Rice transcriptome analysis to identify possible herbicide quinclorac detoxification genes

**DOI:** 10.3389/fgene.2015.00306

**Published:** 2015-09-29

**Authors:** Wenying Xu, Chao Di, Shaoxia Zhou, Jia Liu, Li Li, Fengxia Liu, Xinling Yang, Yun Ling, Zhen Su

**Affiliations:** ^1^State Key Laboratory of Plant Physiology and Biochemistry, College of Biological Sciences, China Agricultural UniversityBeijing, China; ^2^Department of Applied Chemistry, College of Sciences, China Agricultural UniversityBeijing, China

**Keywords:** rice, quinclorac, transcriptome, P450, gene ontology

## Abstract

Quinclorac is a highly selective auxin-type herbicide and is widely used in the effective control of barnyard grass in paddy rice fields, improving the world's rice yield. The herbicide mode of action of quinclorac has been proposed, and hormone interactions affecting quinclorac signaling has been identified. Because of widespread use, quinclorac may be transported outside rice fields with the drainage waters, leading to soil and water pollution and other environmental health problems. In this study, we used 57K Affymetrix rice whole-genome array to identify quinclorac signaling response genes to study the molecular mechanisms of action and detoxification of quinclorac in rice plants. Overall, 637 probe sets were identified with differential expression levels under either 6 or 24 h of quinclorac treatment. Auxin-related genes such as *GH3* and *OsIAA*s responded to quinclorac treatment. Gene Ontology analysis showed that genes of detoxification-related family genes were significantly enriched, including cytochrome P450, GST, UGT, and ABC and drug transporter genes. Moreover, real-time RT-PCR analysis showed that top candidate genes of P450 families such as CYP81, CYP709C, and CYP72A were universally induced by different herbicides. Some Arabidopsis genes of the same P450 family were up-regulated under quinclorac treatment. We conducted rice whole-genome GeneChip analysis and the first global identification of quinclorac response genes. This work may provide potential markers for detoxification of quinclorac and biomonitors of environmental chemical pollution.

## Introduction

Rice (*Oryza sativa*) is a major crop feeding about half of the world's population. However, some grass weeds such as barnyard grass (*Echinochloa crusgalli*) significantly reduce the world's rice yield. Quinclorac (3,7-dichloro-8-quinolinecarboxylic acid) is a herbicide with specific selectivity between annual grass weeds and some graminaceous crops (Koo et al., 1996; Resgalla et al., 2007). Quinclorac is a compound of the chemical group of quinolines with toxicological class III (low toxicity), and in the recent years, it has been widely used on paddy rice fields to effectively control barnyard grass (Resgalla et al., 2007). Quinclorac is a highly selective auxin-type herbicide, originally emerged from lead optimization with more than 3000 synthesized derivatives by BASF and was introduced into rice cultivation systems in 1988 (Haden et al., 1985; Grossmann, 2000b, 2003).

Many studies have examined the mode of quinclorac action including plant uptake, translocation, metabolism, biochemical and molecular mechanism, etc. (Haden et al., 1985; Grossmann, 2000a,b, 2003). For example, the physiological and biochemical basis for quinclorac resistance in a false cleavers (*Galium spurium* L.) biotype was examined for the auxin signal transduction pathway and the mechanism of quinclorac action (Van Eerd et al., 2005). A model of the selective mode of action of quinclorac in grasses was proposed in which, in sensitive grasses, the quinclorac induces ACC (1-aminocyclopropane-1-carboxylic acid) synthase activity in the root, and then, ACC is transported to the shoot, where it is converted to ACC to ethylene and cyanide and causes phytotoxicity, whereas quinclorac cannot induce ACC synthase in resistant grasses (Grossmann, 2000b). The stimulation of ACC synthase acts as the target process responsible for the herbicidal growth inhibition in sensitive grasses, but the overproduction of cyanide (an ethylene co-product) is more important in growth inhibition and the actual cell death response to quinclorac (Grossmann, 1996) because cyanide accumulation in susceptible grasses is the primary phytotoxic compound that causes growth inhibition and tissue necrosis with physiologically damaging concentrations. The model also shows that ethylene further elicits the downward curvature of leaves and stimulates abscisic acid (ABA) biosynthesis through increasing xanthophyll cleavage to the ABA precursor xanthoxin by 9-cis-epoxycarotenoid dioxygenase in the plastid (Hansen and Grossmann, 2000; Grossmann, 2000a; Grossmann et al., 2001; Raghavan et al., 2006; Kraft et al., 2007). There is only a slight change in concentrations of other phytohormones such as gibberellins and cytokinins (Grossmann, 2000a).

Due to widespread use, quinclorac may be transported outside rice fields with the drainage waters, leading to soil and water pollution and other severe environmental health problems. The auxinic herbicide quinclorac, unlike endogenous auxin, has a long-lasting effect, and risk analyses of herbicide quinclorac residues in irrigated rice areas are very important. Recently, deterministic and probabilistic risk analyses were carried out for seven hydrographic basins in the State of Santa Catarina (Brazil) (Resgalla et al., 2007)—quinclorac was the most frequently detected agrochemical residue, occurring in five of seven hydrographic basins. Furthermore, quinclorac residues were also detected in rivers flowing through irrigated rice production areas. The effect of quinclorac on animals and microbes has also been studied. The potential effect on culturable microorganisms was investigated in a flooded paddy soil to which different quinclorac concentrations were added. Quinclorac concentration is a key factor affecting the populations of various culturable microorganisms (Lü et al., 2004a,b, 2006; Chen et al., 2005). In addition, quinclorac caused increased enzyme activity in the brain of silver catfish and inhibitions in muscle tissue (Moraes et al., 2007). Therefore, the quinclorac detoxification analysis is very important for herbicide tolerance of crops and environmental health issues. Enzyme activity analysis showed that superoxide dismutase is critical in the defense against quinclorac-induced oxidative stress (Lu et al., 2007). The action of quinclorac has been broadly studied and discussed (Haden et al., 1985; Grossmann and Kwiatkowski, 2000; Grossmann, 2000a,b, 2003), but molecular-based detoxification analysis of quinclorac remains limited. There have been many reports concerning resistance to and detoxification of other herbicides (Still and Kuzirian, 1967; Shimabukuro, 1975; Dixon et al., 2003; Hirose et al., 2005; Karavangeli et al., 2005; Labrou et al., 2005; Marcacci et al., 2005; Poienaru and Sarpe, 2006). Plant transcriptome mapping studies have become popular in revealing the possible mechanisms of herbicide and insecticide resistance and hormone signal transduction pathways (Hansen and Grossmann, 2000; Zhong and Burns, 2003; Armstrong et al., 2004; Pasquer et al., 2005; Andersson-Gunnerås et al., 2006; Laskowski et al., 2006; Nemhauser et al., 2006; Kim et al., 2007; Lee et al., 2007; Manabe et al., 2007; Shimono et al., 2007; Zhang et al., 2007a,b; Bruce et al., 2008; Poupardin et al., 2008; Vriezen et al., 2008; Wenzel et al., 2008). For example, microarray screening identified up-regulation of benzothiadiazole (BTH)- and salicylic acid (SA)-inducible WRKY transcription factor (TF) genes within 3 h of BTH treatment. Two defense-related genes, encoding a glutathione S-transferase and a cytochrome P450, were regulated by WRKY45 (Laskowski et al., 2006). Among the enzyme systems, glutathione S-transferase and cytochrome P450 monooxygenase are the enzymes most responsible for increasing herbicide metabolism. Using a similar approach, Arabidopsis genes induced by cis-jasmone were revealed to affect the chemical ecology of multitrophic interactions with aphids and their parasitoids, including a cytochrome P450 (Poupardin et al., 2008). Recently, RNA-seq has been applied to characterize the transcriptome responding to quinclorac in barnyard grass (Li et al., 2013a; Yang et al., 2013).

In this study, we carried out rice whole-genome array screening for quinclorac response genes and used real-time RT-PCR validation and bioinformatics data mining. There were two main purposes: to give an overview of the transcription map for rice under quinclorac treatment and global function classification for the response genes; and most importantly, to obtain a number of candidate genes, especially P450 and GST genes, potentially acting as detoxification markers for quinclorac.

## Materials and methods

### Plant material and growth conditions

Rice (*Oryza sativa* L. cv. Nipponbare) seeds were surface-sterilized in 5% sodium hypochlorite for 20 min and washed in distilled water for three or four times, and then germinated in a greenhouse (28/25°C and 12/12 h light/dark cycles with 83% relative humidity). About 3 weeks after germination, rice plants were at the second leaf stage and were treated with different chemicals.

*Arabidopsis thaliana* (Col-0) seeds were surface-sterilized and sown on MS-agar Petri plates. Then seeds were stratified for 3 d at 4°C in darkness, and then transferred into a growth chamber (16/8 h light/dark at 22°C). The plants were grown on MS medium for a further 2 weeks and then treated with quinclorac.

### Chemical treatments and RNA isolation

All pure herbicides were obtained commercially as follows: quinclorac from BASF AG, Ludwigshafen (Germany), bentazone from Sigma Aldrich, clomazone [2-(2-chlorophenyl)methyl-4,4-dimethyl-3-isoxazolidinone] from Amersham Co., metsulfuron-methyl from Aventis Pharma (Frankfurt/M), and methyl viologen (paraquat) from Aldrich Chem. Co. (Cat. No. 85617-7). The chemical structures of these five herbicides are shown in **Figure 4**.

Based on the dosage used in the field and references concerning several sensitivity or resistance experiments (Qian and Ma, 2004; Abdallah et al., 2006; Peng et al., 2008), the plants were sprayed with the chemicals at the following concentrations: 1.65 mM of quinclorac, 19.98 mM of bentazone, 1.31 mM of metsulfuron-methyl, 1.75 mM of clomazone, and 10 μM of methyl viologen, with 1% DMSO (dimethyl sulfoxide) as a solvent in all cases. The plant samples were harvested after chemical treatment, frozen in liquid nitrogen, and stored at −80°C for further analysis. Control samples (treated with 1% DMSO) were also harvested at the same time. Rice shoots were treated with quinclorac for 3, 6, and 24 h, and the Arabidopsis seedlings were treated with quinclorac for 6 and 12 h. Rice shoots were also treated with the other chemicals for 6 h.

Total RNA was extracted using TRizol (Invitrogen, CA, USA) and purified by using Qiagen RNeasy kit (Qiagen, Hilden, Germany). OD260/OD280 and OD260/OD230 measurements were taken to determine the concentration and quality of RNA solution using an Eppendorf Biophotometer.

### Affymetrix GeneChip analysis

For each sample, 8 μg of total RNA was used to make biotin-labeled cRNA targets. All the processing for cDNA and cRNA synthesis, cRNA fragmentation, hybridization, washing and staining, and scanning, followed the GeneChip Standard Protocol (Eukaryotic Target Preparation). In this experiment, a Poly-A RNA Control Kit and a One-Cycle cDNA Synthesis kit were applied. Affymetrix GCOS software was used for data normalization and comparative analysis. Raw chip data for the expression profilings are shown in Data Sheet 1.

### Reverse transcription and real-time RT-PCR

Reverse transcription was performed with 10-μl samples containing 2 μg of total RNA using M-MLV (Invitrogen). The cDNA samples were diluted to 8 ng/μl. Triplicate quantitative assays were performed on 1 μl of each cDNA dilution using the SYBR Green Master Mix (Applied Biosystems, PN 4309155) with an ABI 7900 sequence detection system according to the manufacturer's protocol (Applied Biosystems). The gene-specific primers were designed by using Primer Express software. The amplification of 18S rRNA was used as an internal control to normalize all data (forward primer, 5′-CGGCTACCACATCCAAGGAA-3′ and reverse primer, 5′-TGTCACTACCTCCCCGTGTCA-3′). Gene-specific primers are listed in Supplemental Table 2. The relative quantification method (ΔΔCT) was used to evaluate quantitative variation between the replicates examined.

### Microarray data analysis

All CEL files were processed using Affymetrix GCOS software to produce the CHP file, and the TGT (target mean value) was scaled as 500 for each chip. The raw expression data is listed in the Data Sheet 1.

The signal intensity for each probe set of GeneChip microarray was extracted by MAS 5.0 algorithm. To determine differentially expressed genes between quinclorac treatment and control samples, the log_2_-transformed signal ratio of each gene was calculated by applying the GCOS baseline tool. Another, algorithm dChip was also applied to these raw chip data, and the expression difference of each gene was calculated by the “compare samples” tool. For each time point (6 or 24 h treatment), the probe set assigned significant changes according to the following rules: the expression ratios (quinclorac vs. mock treatment) were ≥ 2 [i.e., signal log ratio (SLR) ≥ 1 or SLR ≤ −1] in both biological duplicates based on MAS 5.0 algorithm; the difference in expression levels was considered as significant based on dChip algorithm.

EasyGO (http://bioinformatics.cau.edu.cn/easygo) (Zhou and Su, 2007) and GeneBins (Goffard and Weiller, 2007) were used for functional categorization. MapMan (http://mapman.gabipd.org) was used for key regulation group analysis.

## Results

### A microarray-based screen of quinclorac-regulated transcripts

To identify quinclorac signaling response genes, 57K Affymetrix rice whole-genome array was used to generate the expression patterns. To simulate the field effect (Qian and Ma, 2004; Abdallah et al., 2006; Peng et al., 2008), we sprayed 3-week-old rice (Nipponbare in *japonica* cultivar-group) plants with 1.65 mM quinclorac (about 400 mg/L) and 1% DMSO as mock and extracted RNAs from shoot tissue samples of quinclorac- and mock-treated plants at 6 and 24 h after treatment. Two sets of biological duplicates were collected and a total of eight chips were analyzed.

**Figure 1:**
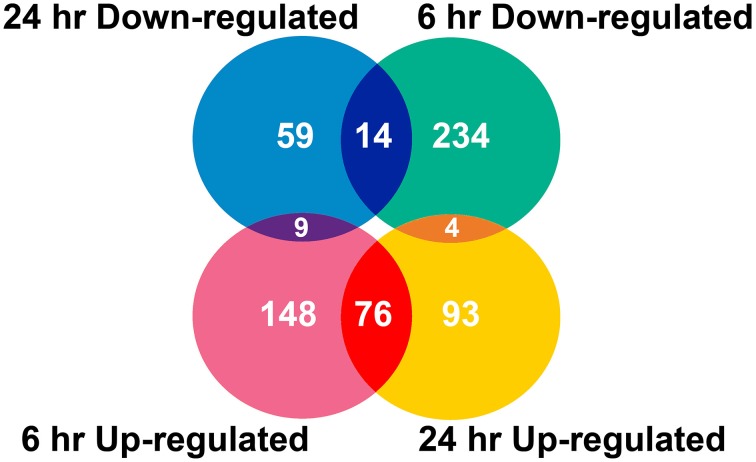
**Venn diagram of rice genes (probe sets) responded to quinclorac**.

Two algorithms were applied to the GeneChip data: MAS5 and dChip (Li and Wong, 2001). The change of expression level for each gene under quinclorac treatment was compared with mock treatment using the MAS5 algorithm through GCOS baseline tool and the dChip sample comparison tool. We selected the probe sets that were considered to change significantly during quinclorac treatment by both algorithms and in both two duplicates in 6 or 24 h. In total, there were 637 probe sets with the expression level changed either at 6 or at 24 h quinclorac treatment (*p* ≤ 0.05); these genes were considered as quinclorac response genes for further analysis. The detailed expression level (based on MAS5 algorithm) and annotation of each probe set are listed in Supplemental Table 1. Among the 637 probe sets, 626 probe sets were associated to 551 locus IDs based on the annotations from TIGR Rice Genome version 5. There were 233 probe sets up-regulated with 6 h and 173 up-regulated with 24 h quinclorac treatment, and 76 of these were up-regulated for both 6 and 24 h; there were 252 probe sets down-regulated with 6 h and 82 down-regulated with 24 h quinclorac treatment, and 14 of these were down-regulated for both 6 and 24 h (Figure 1).

### Gene ontology (GO)-based functional categorization of quinclorac response genes

**Figure 2:**
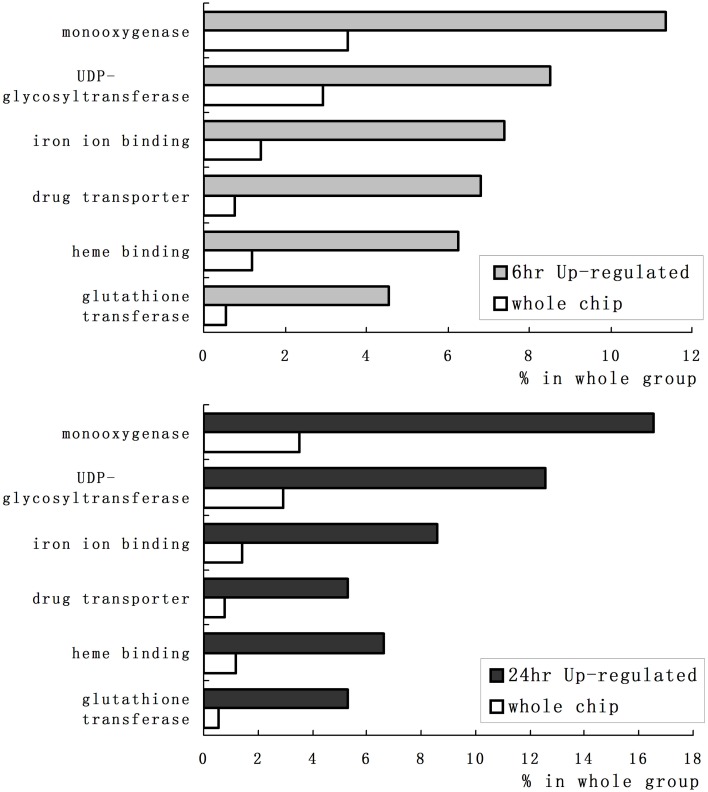
**The significantly enriched GO terms of the quinclorac-induced genes**. The white bars represent the percentage of each GO terms in rice whole-genome chip, the light gray bars (top) represent the percentage of each GO terms in the quinclorac-induced genes at 6 h of treatment, the dark gray bars (bottom) represent the percentage of each GO terms in the quinclorac-induced genes at 24 h of treatment.

We employed GO category enrichment analysis to obtain an overview of these quinclorac response genes. We searched the GO terms using EasyGO (Zhou and Su, 2007) for respective up- and down-regulated probe sets at 6 and 24 h treatments. Using *p* ≤ 0.05 as cut-off, there were no significant GO categories within the down-regulated probe sets; however, there were significantly enriched GO terms in both 6 and 24 h up-regulated probe sets. Supplemental Figure 1 shows the GO category results of molecular function enrichment analysis. Among these up-regulated probe sets, several GO terms including monooxygenase, iron ion binding, heme binding, UDP-glycosyltransferase, drug transporter, and glutathione transferase were significantly enriched both in 6 and 24 h quinclorac treatment. These GO terms enriched > 2-folds are shown in Figure 2. Supplemental Figure 2 shows the enriched GO categories of biological process, several GO terms including toxin catabolic process, electron transport, and drug transport, and regulation of transcription were significantly enriched under quinclorac treatment.

Furthermore, we classified these quinclorac-regulated genes using another analysis tool, GeneBins (Goffard and Weiller, 2007). Supplemental Figure 3 highlights several classes (BINs) of genes significantly up-regulated at 6 and 24 h treatment including glutathione S-transferase, flavonol 3-O- glycosyltransferase, unspecific monooxygenase, membrane transport, glutathione metabolism, and tryptophan metabolism.

The results of the two independent tools highlighted similar groups. In addition, most probe sets highlighted in GO term monooxygenase, iron ion binding, and heme binding were cytochrome P450 genes. Four GO terms and function categorization were highlighted in the quinclorac up-regulated genes including glutathione S-transferase, P450 family genes, UDP-glycosyltransferase, and drug transporter. Listed genes for these four families extracted from the 637 probe sets are shown in Table 1.

**Table 1:** **The quinclorac-responsive genes related to significant GO categories**.

**Probe Set ID**	**Locus ID**	**6 h**	**24 h**	**Family/member**
**CYTOCHROME P450**				
Os.4291.1.S1_at	LOC_Os06g03930	↑	↑	CYP704A4
Os.9067.1.S1_at	LOC_Os07g44140	↑	↑	CYP709C5
Os.9017.1.S1_x_at	LOC_Os07g23570	↑	↑	CYP709C9
Os.767.1.S1_at	LOC_Os01g43700	↑	↑	CYP72A17
Os.6452.2.A1_a_at	LOC_Os01g43710	↑	↑	CYP72A18
Os.6452.1.S1_at	LOC_Os01g43710	–	↑	CYP72A18
Os.11193.1.S1_at	LOC_Os03g55240	↑	↑	CYP81A6
Os.17865.1.S1_at	LOC_Os10g36980	↑	↑	CYP89B9
Os.12145.1.S1_at	LOC_Os02g47470	↑	–	CYP707A5
Os.30664.1.S1_at	LOC_Os01g12740	↑	–	CYP71T1
Os.45887.1.S1_at	LOC_Os01g12750	↑	–	CYP71T2
Os.53884.1.S1_at	LOC_Os05g25640	↑	–	CYP73A38
Os.10430.1.S1_at	LOC_Os03g55260	↑	–	CYP81A8
OsAffx.13348.1.S1_s_at	LOC_Os03g44740	↑	–	CYP92C1
Os.25621.2.S1_at	LOC_Os12g16720	–	↑	CYP71P1
OsAffx.25718.1.S1_at	LOC_Os03g55250	–	↑	CYP81A7
Os.38346.1.S1_x_at	LOC_Os01g36350	↓	–	CYP71C18P
Os.22415.1.S1_s_at	LOC_Os06g43430	↓	–	CYP71K9
Os.8493.1.A1_at	LOC_Os06g43430	↓	–	CYP71K9
Os.43417.1.S1_at	LOC_Os01g12760	↓	–	CYP71T3
Os.27715.1.S1_a_at	LOC_Os10g08474	↓	–	CYP76H8
Os.15537.1.S1_at	LOC_Os08g39660	↓	–	CYP76M10
Os.49566.1.S1_at	LOC_Os06g22340	↓	–	CYP89C1
Os.54421.1.S1_at	LOC_Os05g37250	–	↓	CYP94C4
Os.10689.1.S1_at	LOC_Os06g02019	↓	↓	CYP88A5
**GLUTATHIONE S-TRANSFERASE**				
Os.46635.1.S1_x_at	LOC_Os10g38350	↑	–	Glutathione S-transferase GSTU6
Os.2612.1.S1_at	LOC_Os10g38495	↑	↑	Glutathione S-transferase GSTU6
Os.7794.1.S1_at	LOC_Os10g38501	–	↑	Glutathione S-transferase GSTU6
Os.15699.1.S1_at	LOC_Os10g38590	↑	↑	Glutathione S-transferase GSTU6
Os.4762.1.S1_at	LOC_Os10g38740	↑	↑	Glutathione S-transferase GSTU6
Os.9101.1.S1_at	LOC_Os10g38780	↑	↑	Glutathione S-transferase GSTU6
Os.7911.1.S1_at	LOC_Os01g27210	↑	↑	Glutathione S-transferase IV
Os.12200.1.S1_s_at	LOC_Os03g57200	↑	↑	Glutathione S-transferase parA
Os.49030.1.A1_s_at	LOC_Os09g20220	↑	↑	Glutathione S-transferase
**UDP-GLUCOSYL TRANSFERASE**				
Os.46328.1.S1_at	LOC_Os04g46980	–	↑	Cis-zeatin O-glucosyltransferase 1
Os.46328.1.S1_x_at	LOC_Os04g46980	–	↑	Cis-zeatin O-glucosyltransferase 1
Os.14631.1.S1_at	LOC_Os07g13770	↑	↑	Cytokinin-N-glucosyltransferase 1
Os.12253.1.S1_at	LOC_Os01g45110	↑	↑	Cytokinin-O-glucosyltransferase 1
Os.28129.1.S1_at	LOC_Os05g42040	↑	–	Cytokinin-O-glucosyltransferase 1
OsAffx.11989.2.S1_s_at	LOC_Os02g11700	↑	↑	Cytokinin-O-glucosyltransferase 3
Os.32668.1.S1_at	LOC_Os01g08090	↑	↑	Flavonol-3-O-glycoside-7-O-glucosyltransferase 1
Os.10348.1.S1_at	LOC_Os01g41430	–	↑	Flavonol-3-O-glycoside-7-O-glucosyltransferase 1
Os.8045.1.S1_at	LOC_Os01g08440	↑	–	Indole-3-acetate beta-glucosyltransferase
Os.39636.1.A1_x_at	LOC_Os04g12720	↑	–	Indole-3-acetate beta-glucosyltransferase
Os.6101.1.S1_at	LOC_Os04g12970	↑	↓	Indole-3-acetate beta-glucosyltransferase
Os.51337.1.S1_at	LOC_Os06g39330	↑	–	Indole-3-acetate beta-glucosyltransferase
Os.10546.1.S1_s_at	LOC_Os09g34230	↑	↑	Indole-3-acetate beta-glucosyltransferase
OsAffx.30138.1.S1_at	LOC_Os09g34230	↑	↑	Indole-3-acetate beta-glucosyltransferase
Os.48216.1.S1_at	LOC_Os09g34250	↑	↑	Indole-3-acetate beta-glucosyltransferase
**TRANSPORTERS**				
Os.467.1.S1_a_at	LOC_Os01g42380	↑	–	ATP-binding Cassette (ABC)
Os.11800.1.S1_at	LOC_Os01g50100	↑	–	ATP-binding Cassette (ABC)
Os.11800.1.S1_s_at	LOC_Os01g50100	↑	–	ATP-binding Cassette (ABC)
Os.54482.1.S1_at	LOC_Os02g32690	↑	↑	ATP-binding Cassette (ABC)
OsAffx.24543.1.S1_at	LOC_Os02g32690	↑	↑	ATP-binding Cassette (ABC)
Os.24644.1.S1_at	LOC_Os04g49890	–	↑	ATP-binding Cassette (ABC)
Os.27657.1.A1_at	LOC_Os04g49890	–	↑	ATP-binding Cassette (ABC)
OsAffx.29871.1.S1_x_at	LOC_Os09g16330	–	↑	ATP-binding Cassette (ABC)
Os.48761.1.S1_at	LOC_Os02g27490	↑	–	Bile Acid:Na+ Symporter (BASS)
Os.27828.1.S1_a_at	LOC_Os05g51610	↓	–	Ca2+:Cation Antiporter (CaCA)
Os.14849.1.S1_a_at	LOC_Os06g49500	–	↓	Drug/Metabolite Transporter (DMT)
Os.17405.1.S1_a_at	LOC_Os12g31860	↓	–	Drug/Metabolite Transporter (DMT)
Os.53009.1.A1_x_at	LOC_Os02g54640	↓	↓	Glutamate-gated Ion Channel (GIC) of Neurotransmitter Receptors
Os.50175.2.S1_at	LOC_Os04g51820	↑	–	K+ Transporter (Trk)
Os.16325.1.S1_at	LOC_Os03g37830	↑	–	K+ Uptake Permease (KUP)
Os.8504.1.S1_at	LOC_Os04g42420	↑	–	Major Facilitator (MFS)
Os.55618.1.S1_at	LOC_Os07g37454	↓	↓	Major Facilitator (MFS)
Os.18177.1.S1_at	LOC_Os11g04104	↑	↑	Major Facilitator (MFS)
Os.9752.3.S1_x_at	LOC_Os11g04104	↑	↑	Major Facilitator (MFS)
Os.9752.1.S1_a_at	LOC_Os12g03899	↑	↑	Major Facilitator (MFS)
Os.9752.2.S1_x_at	LOC_Os12g03899	↑	↑	Major Facilitator (MFS)
Os.9556.1.S1_at	LOC_Os12g44060	↓	–	Major Facilitator (MFS)
Os.409.1.S1_at	LOC_Os07g15460	↑	↓	Metal Ion (Mn2+-iron) Transporter (Nramp)
OsAffx.17942.1.S1_at	LOC_Os09g28160	↑	–	Mitochondrial Carrier (MC)
Os.12162.1.S1_at	LOC_Os10g42299	–	↑	Mitochondrial Carrier (MC)
Os.4700.1.S1_at	LOC_Os07g47100	↑	–	Monovalent Cation:Proton Antiporter-1 (CPA1)
Os.43896.1.S1_at	LOC_Os03g08900	↑	↑	Multidrug/Oligosaccharidyl-lipid/Polysaccharide (MOP) Flippase
Os.6998.1.S1_at	LOC_Os03g37490	↑	↑	Multidrug/Oligosaccharidyl-lipid/Polysaccharide (MOP) Flippase
OsAffx.26254.1.S1_at	LOC_Os04g30490	↑	–	Multidrug/Oligosaccharidyl-lipid/Polysaccharide (MOP) Flippase
Os.6517.1.S1_at	LOC_Os08g37432	↑	↑	Multidrug/Oligosaccharidyl-lipid/Polysaccharide (MOP) Flippase
OsAffx.29571.1.S1_at	LOC_Os08g37432	↑	–	Multidrug/Oligosaccharidyl-lipid/Polysaccharide (MOP) Flippase
Os.53137.1.S1_at	LOC_Os07g09300	↓	↓	Proton-dependent Oligopeptide Transporter (POT)
Os.10754.1.S1_at	LOC_Os03g06520	↑	–	Sulfate Permease (SulP)
Os.18597.1.S1_at	LOC_Os03g09930	↑	–	Sulfate Permease (SulP)

The names of rice P450 genes are based on the nomenclature of Nelson's paper (Nelson et al., 2004). There were 25 P450 genes (belong to 17 sub-families) showing significant change under quinclorac treatment; notably seven of them were induced by quinclorac both for 6 and 24 h: CYP81A6, CYP709C5, CYP709C9, CYP72A17, CYP72A18, CYP89B9, and CYP704A4. In addition to these seven P450 genes, six genes were only up-regulated by quinclorac at 6 h, two genes were only up-regulated at 24 h, one gene was down-regulated both at 6 and 24 h, six genes were only down-regulated at 6 h, and one gene was only down-regulated at 24 h.

All nine glutathione S-transferase family genes were up-regulated under quinclorac treatment. Seven of them were induced by quinclorac both at 6 and 24 h, including six GSTU6 genes on chromosome 10. Among 13 UDP-glucosyl transferase family genes, 12 were up-regulated by quinclorac treatment including five indole-3-acetate beta-glucosyltransferase genes and four cytokinin-related glucosyltransferase genes. The one exception was LOC_Os04g12970 (indole-3-acetate beta-glucosyltransferase), which was up-regulated at 6 h and down-regulated at 24 h of treatment.

Table 1 lists 28 transport genes with up- or down-regulation under quinclorac treatment: 20 of which were up-regulated and 7 down-regulated; and one gene, LOC_Os07g15460 (metal transporter Nramp6), was up-regulated at 6 h and down-regulated at 24 h of treatment. Of different transport protein families, all five ABC genes and four Multidrug/Oligosaccharidyl-lipid/Polysaccharide (MOP) Flippase genes were up-regulated under treatment; among five major facilitator (MFS) genes, two were down-regulated and the other three were up-regulated. The other quinclorac-induced transporter genes included K+ Transporter (Trk), K+ Uptake Permease (KUP), Mitochondrial Carrier (MC), Monovalent Cation: Proton Antiporter-1 (CPA1), Oligopeptide Transporter, and Sulfate Permease (SulP).

### Regulatory proteins including transcription factor and kinase genes in the quinclorac response genes

We further analyzed some specific regulation functional groups. Based on MapMan analysis (Thimm et al., 2004), we found that a large number of transcription factor (TF) and other regulatory protein genes responded to quinclorac treatment (Supplemental Figure 4). TFs are well known to be primarily involved in the initiation stage of RNA transcription, and they are the key factors in regulating gene expression level. There were 48 diverse TF genes up- or down-regulated by quinclorac treatment (Table 2). Most genes in BHLH and HSF were up-regulated, whereas C2C2-CO-like genes, C2H2, and C3H genes were significantly down-regulated under quinclorac treatment. In addition, some abiotic and biotic stress-related TF genes also responded to quinclorac treatment; they belonged to various families such as ARF, AUX/IAA AP2-EREBP, bZIP, MYB, NAC, ZIM, and WRKY families.

**Table 2:** **Regulatory proteins responded to quinclorac treatment**.

**Probe Set ID**	**Locus ID**	**6 h**	**24 h**	**Family/member**
**TRANSCRIPTION FACTORS**				
Os.8031.1.S1_at	LOC_Os01g21120	↑	–	AP2-EREBP
OsAffx.12799.1.S1_s_at	LOC_Os03g09170	↑	↑	AP2-EREBP
Os.55386.1.A1_at	LOC_Os10g41330	↓	–	AP2-EREBP
Os.8374.1.S1_at	LOC_Os04g43910	↑	–	ARF
Os.11608.1.S1_at	LOC_Os01g53880	↓	–	AUX/IAA
Os.17655.1.S1_at	LOC_Os02g49160	↓	–	AUX/IAA
Os.8585.1.S1_at	LOC_Os03g53150	–	↑	AUX/IAA
Os.26512.1.S1_at	LOC_Os03g58350	↑	–	AUX/IAA
Os.8622.1.S1_at	LOC_Os07g08460	↑	–	AUX/IAA
Os.10300.1.S1_at	LOC_Os01g01840	↑	–	bHLH
Os.10300.1.S1_x_at	LOC_Os01g01840	↑	–	bHLH
Os.12498.1.S1_at	LOC_Os01g72370	↑	–	bHLH
Os.9216.1.S1_at	LOC_Os03g26210	↑	–	bHLH
Os.51063.1.S1_at	LOC_Os09g28210	–	↑	bHLH
Os.35049.1.S1_a_at	LOC_Os01g59350	↓	–	bZIP
Os.26437.1.A1_at	LOC_Os12g40920	↑	–	bZIP
Os.26437.1.A1_s_at	LOC_Os12g40920	↑	–	bZIP
OsAffx.20062.1.S1_at	LOC_Os12g40920	↑	–	bZIP
Os.3400.1.S1_s_at	LOC_Os02g49230	↓	–	C2C2-CO-like
Os.50246.2.S1_x_at	LOC_Os02g49880	↓	–	C2C2-CO-like
Os.12977.1.S1_at	LOC_Os06g19444	↓	–	C2C2-CO-like
Os.21282.1.S1_at	LOC_Os03g13600	↓	–	C2H2
Os.23145.1.S1_at	LOC_Os08g03310	↓	–	C3H
Os.37238.1.S1_at	LOC_Os08g09690	–	↑	CCAAT-HAP2
Os.49245.1.S1_at	LOC_Os02g43330	↑	↑	HB
Os.40021.1.S1_a_at	LOC_Os03g06630	–	↑	HSF
Os.2292.3.S1_x_at	LOC_Os03g53340	–	↑	HSF
Os.39876.1.S1_at	LOC_Os04g48030	–	↑	HSF
Os.50642.1.S1_at	LOC_Os04g48030	–	↑	HSF
Os.40018.1.S1_at	LOC_Os05g45410	↑	–	HSF
Os.10570.1.S1_at	LOC_Os06g35960	↑	↑	HSF
Os.27176.1.S1_at	LOC_Os09g28354	–	↑	HSF
Os.11941.2.S1_at	LOC_Os09g35790	–	↑	HSF
Os.54707.1.S1_x_at	LOC_Os09g35790	↓	↑	HSF
OsAffx.30145.1.S1_at	LOC_Os09g35790	–	↑	HSF
Os.10942.1.S1_a_at	LOC_Os10g28340	–	↑	HSF
Os.1726.1.S1_at	LOC_Os01g18240	↑	–	MYB
Os.408.1.S1_a_at	LOC_Os01g74410	↓	–	MYB
Os.3386.1.S1_x_at	LOC_Os06g10350	–	↓	MYB
Os.10174.1.S1_at	LOC_Os11g47460	↓	–	MYB
Os.41164.1.S1_at	LOC_Os01g44390	↓	–	MYB-related
Os.35196.1.S1_at	LOC_Os05g51160	↓	–	MYB-related
Os.10901.1.S1_a_at	LOC_Os08g06110	↑	–	MYB-related
Os.26695.1.S1_at	LOC_Os03g60080	–	↑	NAC
Os.37548.1.S1_at	LOC_Os05g34830	–	↑	NAC
Os.5549.1.S1_at	LOC_Os07g12340	–	↑	NAC
Os.15198.1.S1_at	LOC_Os01g10580	–	↓	Orphans
Os.48625.1.A1_s_at	LOC_Os05g51690	↑	–	Orphans
Os.9713.1.S1_at	LOC_Os05g51690	↑	–	Orphans
OsAffx.30176.1.S1_at	LOC_Os09g37710	↑	↑	RWP-RK
Os.29979.1.S1_at	LOC_Os01g09100	–	↓	WRKY
Os.37565.2.S1_at	LOC_Os05g25770	↑	↑	WRKY
Os.11404.1.S1_s_at	LOC_Os08g17400	↓	–	WRKY
Os.27039.1.S1_at	LOC_Os08g38990	↓	–	WRKY
Os.25606.1.S1_at	LOC_Os09g25060	↑	↑	WRKY
**KINASES AND PP2C**				
Os.18633.1.S1_at	LOC_Os04g43710	↓	–	CAMK_like(CAMK_1)
Os.19584.1.A1_at	LOC_Os07g35390	–	↓	DUF26-Ic
Os.26816.1.A1_s_at	LOC_Os07g35410	↓	–	DUF26-Ic
Os.27185.1.S1_x_at	LOC_Os07g35750	↓	–	DUF26-Id
Os.12664.1.S1_at	LOC_Os07g35810	↓	–	DUF26-Id
Os.4627.1.S1_x_at	LOC_Os07g48100	↑	–	KIN1/SNF1/Nim1_like(CAMK_2)
Os.19031.1.A1_at	LOC_Os09g18360	–	↓	LRR-Ia
Os.14862.1.S1_a_at	LOC_Os08g10320	↓	–	LRR-VI
Os.14862.1.S1_s_at	LOC_Os08g10320	↓	–	LRR-VI
Os.52345.1.S1_at	LOC_Os01g60280	↑	–	LRR-VIII-1
Os.17824.1.S1_x_at	LOC_Os02g42620	↓	–	LRR-VIII-2
Os.17190.1.A1_s_at	LOC_Os11g36150	↓	–	LRR-XII
Os.20861.1.S1_at	LOC_Os11g36150	↓	–	LRR-XII
Os.26792.1.S1_at	LOC_Os11g36160	↓	–	LRR-XII
Os.27092.1.S1_at	LOC_Os07g08000	↓	–	NimA_NEK_like
Os.7304.1.S1_at	LOC_Os01g10450	↑	–	Raf
Os.17144.1.A1_at	LOC_Os04g59000	↓	–	Raf
Os.52163.1.S1_at	LOC_Os06g35850	↓	–	SD-2b
Os.23327.2.S1_a_at	LOC_Os10g10130	↓	–	WAKb
Os.15191.1.S1_s_at	LOC_Os05g41220	↓	–	SNF1-related protein kinase regulatory subunit beta-1
Os.5367.1.S1_at	LOC_Os01g40094	↑	↑	Protein phosphatase 2C ABI2
Os.27906.1.S1_a_at	LOC_Os01g62760	↑	–	Protein phosphatase 2C
Os.9022.1.S1_at	LOC_Os09g15670	↑	–	Protein phosphatase 2C

We found an interesting phenomenon during gene function classification, in that most kinase genes were down-regulated under quinclorac treatment, especially at 6 h treatment. We listed 18 kinase genes; 15 of which were down-regulated under quinclorac treatment including four members from the DUF26 family and five from LRR families (Table 2). However, three PP2Cs including ABI2 were up-regulated under quinclorac treatment.

### Real-time RT-PCR validation of microarray data

**Figure 3:**
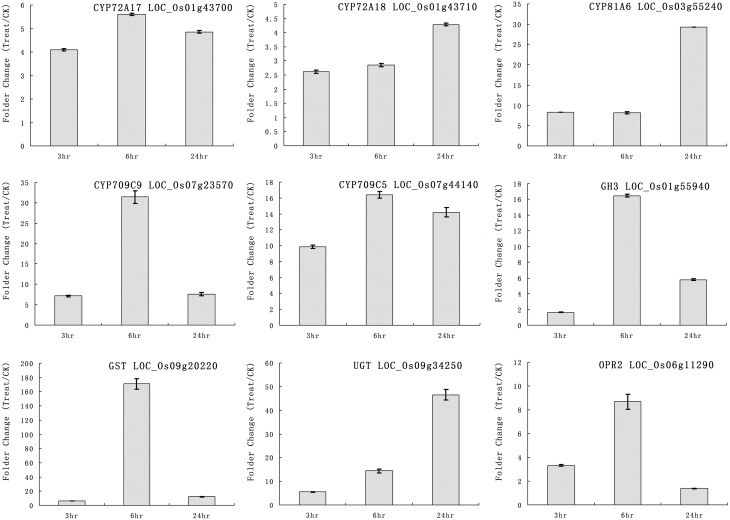
**The expression pattern of selected rice genes responded to different times of quinclorac treatment**. The expression values were detected by real-time RT-PCR. The gray bars indicate the fold change of the gene between quinclorac treatment and mock; the error bars represent the standard error of three replicates. LOC_Os01g43700—CYP72A17; LOC_Os01g43710—CYP72A18; LOC_Os03g55240—CYP81A6; LOC_Os07g23570—CYP709C9; LOC_Os07g44140—CYP709C5; LOC_Os01g55940—GH3; LOC_Os09g20220—GST; LOC_Os09g34250—UGT; LOC_Os06g11290—OPR2.

We selected some genes for real-time RT-PCR analysis under quinclorac treatment including five P450 genes, one GH3 (auxin-responsive GH3 gene), one GST (glutathione S-transferase), one UGT (indole-3-acetate beta-glucosyltransferase), and OPR2 (12-oxophytodienoate reductase 2). The five cytochrome P450 genes were induced by quinclorac treatment: CYP72A17, CYP72A18, CYP81A6, CYP709C5, and CYP709C9 (Figure 3). CYP81A6 was much more induced at 24 h, and CYP709C9 was much more induced at 6 h; CYP72A17 and CYP709C5 were highly induced in similar level all the time, whereas CYP72A18 was only slightly induced at 24 h. All the other genes were induced by quinclorac, most reaching a peak at 6 h, and only UGT (LOC_Os09g34250) kept increasing throughout the treatment time.

## Discussion

### Cross-talk among hormone-related genes under quinclorac treatment in rice

Because quinclorac is an auxin-type herbicide, we investigated the response of auxin and other hormone-related genes under quinclorac treatment using MapMan in which we assigned the rice GeneChip probe sets by BLAST against Arabidopsis genes using 1.0E-5 as the e-value cut-off. Auxin and other hormone-related genes were up- or down-regulated under quinclorac treatment (Supplemental Figure 4). While searching the MapMan database, we found that auxin-related genes significantly responded to quinclorac treatment including *GH3* (indole-3-acetic acid-amido synthetase), *OsIAA* (auxin-responsive Aux/IAA), *OsSAUR39*, and *IN2-2* genes. Most auxin-related genes were up-regulated, especially at 24 h of treatment. Ethylene has a crucial role in the herbicide mode of action of quinclorac (Grossmann and Scheltrup, 1997; Grossmann, 2000a,b). Based on microarray data, some rice ethylene response genes were down-regulated under quinclorac treatment. Other plant hormone gene expression was also investigated through microarray analysis, such SA and GA. This suggested that there may be cross-talks between herbicide quinclorac and plant hormones.

### Comparison between quinclorac and other herbicide-response genes

GO analysis for the quinclorac response genes showed that three families of genes were significantly enriched including cytochrome P450 family genes, glutathione S-transferase (GST), and UDP-glycosyltransferase. Especially, P450 enzymes are the most important enzyme system for phase I herbicide metabolism in plants, joining GST (phase II metabolism) as the primary enzyme system metabolizing exogenous chemicals entering the plant system. GSTs found in all eukaryotes detoxify electrophilic xenobiotics including herbicides by catalyzing their conjugation with glutathione. Several quinclorac-response GST genes were recently identified in *E. crusgalli* recently (Li et al., 2013b). Following the hydroxylation of herbicides by P450 monooxygenases, the UDP-glucosyltransferases typically add glucose to herbicides. These gene families may be associated with general herbicide detoxification. We compared the rice genes responding to different herbicide treatments, such as bentazone, metsulfuron-methyl, clomazone, and methyl viologen.

We performed text mining based on the paper from Shimono (Shimono et al., 2007), which showed the microarray data on rice genes responding to bentazone treatment. Bentazone, a benzothiadiazinone contact herbicide, is normally used for controlling many broadleaf weeds and sedges including rice and leguminous crops (McFadden et al., 1990). We re-mapped the locus ID from the TIGR database to the gene names used in Shimono's paper and listed the rice genes responding to both quinclorac and bentazone treatments (Table 3). The herbicide quinclorac up-regulated P450, GST, and UGT genes were up-regulated under bentazone treatment. Notably, some P450 genes (Table 3) were already widely utilized for engineering crops for trait improvements. For example, CYP81A6 was identified as conferring resistance to two different classes of herbicides including bentazone and sulfonylurea (Pan et al., 2006). We also found that the TF genes such as WRKY45 and WRKY76 were up-regulated under both quinclorac and bentazone treatments. WRKY45 regulated glutathione S-transferase (such as GSTU6) and cytochrome P450 (such as CYP709C9) during bentazone treatment (Shimono et al., 2007). Most bentazone-responsive genes in Arabidopsis were reported to be regulated by NPR (Wang et al., 2006). We found that a *NPR1* gene was up-regulated in both herbicide treatments. These results suggest that these two herbicides may share a similar detoxification pathway in rice.

**Table 3:** **List of selected rice genes responded to both quinclorac and bentazone treatment**.

**Locus ID (TIGR)**	**Quinclorac/mock**		**Gene name**	**BTH/mock**		**Description**
	**6 h**	**24 h**		**Fold change**	**FDR (q-value)**	
**CYTOCHROME P450 AND OTHER OXIDOREDUCTASE**						
LOC_Os01g43710	1.45	1.7	Os01g0627500	4.27	0.0357	Cytochrome P450 72A18
LOC_Os12g16720	1.05	1.4	Os12g0268000	8.99	0.0178	Cytochrome P450 71A1
LOC_Os07g23570	3.55	7.2	Os07g0418500	38.63	< 1.00E-05	Cytochrome P450 709C9
LOC_Os07g44140	2.15	3.75	Os07g0635500	25.46	0.0002	Cytochrome P450 709C5
LOC_Os07g44440	2.8	3.55	Os07g0638400	42.41	0.0001	Peroxiredoxin
LOC_Os06g11290	1.35	1.6	Os06g0216300	5.63	0.0067	12-oxophytodienoate reductase 2
LOC_Os10g40934	−1.3	−0.2	Os10g0558700	6.33	0.0012	Flavonol synthase/flavanone 3-hydroxylase, similar to SRG1
**GLUTATHIONE S-TRANSFERASE**						
LOC_Os10g38501	0.85	1.4	Os10g0528400	9.05	0.0001	Glutathione S-transferase GSTU6
LOC_Os10g38495	2.5	3.2	Os10g0528300	97.76	< 1.00E-05	Glutathione S-transferase GSTU6
LOC_Os10g38780	1.3	1.75	Os10g0531400	9.77	4.00E-05	Glutathione S-transferase GSTU6
LOC_Os09g20220	4.4	2.75	Os09g0367700	51.45	0.0001	Glutathione S-transferase
LOC_Os03g57200	2.5	4.05	Os03g0785900	6.72	0.0016	Glutathione S-transferase parA
**UDP-GLUCOSYL TRANSFERASE**						
LOC_Os01g45110	1.85	3.05	Os01g0638000	23.42	< 1.00E-5	Cytokinin-O-glucosyltransferase 1
LOC_Os01g08090	1.45	2.4	Os01g0176000	37.31	3.00E-05	Flavonol-3-O-glycoside-7-O-glucosyltransferase 1
LOC_Os01g41430	1.05	1.35	Os01g0597800	28.74	0.0061	Flavonol-3-O-glycoside-7-O-glucosyltransferase 1
LOC_Os09g34230	2.25	1.9	Os09g0518000	21.58	< 1.00E-05	Indole-3-acetate beta-glucosyltransferase
LOC_Os09g34250	5.55	5.85	Os09g0518200	60.23	< 1.00E-05	Indole-3-acetate beta-glucosyltransferase
**TRANSCRIPTION REGULATOR**						
LOC_Os05g25770	1.5	1	Os05g0322900	35.78	< 1.00E-05	OsWRKY45 - Superfamily of rice TFs having WRKY and zinc finger domains
LOC_Os09g25060	2.2	1.6	Os09g0417600	312.93	< 1.00E-05	OsWRKY76 - Superfamily of rice TFs having WRKY and zinc finger domains
LOC_Os01g01840	3.1	1.05	Os01g0108400	163.74	< 1.00E-05	Helix-loop-helix DNA-binding domain containing protein
LOC_Os05g45410	1.1	0.05	Os05g0530400	5.97	0.0038	Heat shock factor
LOC_Os03g46440	1.65	0.8	Os03g0667100	6.81	0.0015	Regulatory protein NPR1
LOC_Os02g45780	0.15	1.35	Os02g0682300	5.44	0.0047	Protein binding protein
**OTHERS**						
LOC_Os09g28160	1.1	0.85	Os09g0454600	5.77	0.0043	phosphate carrier protein, mitochondrial precursor
LOC_Os03g08900	1.95	2.75	Os03g0188100	27.38	< 1.00E-05	Transparent testa 12 protein
LOC_Os03g05750	0.4	2.3	Os03g0152000	4.78	0.0153	Metal ion binding protein
LOC_Os12g36910	1.3	1	Os12g0556200	8.66	0.0002	Calmodulin binding protein
LOC_Os04g58090	1.25	0.6	Os04g0677300	4.84	0.0267	NHL25
LOC_Os04g27060	0.95	2.25	Os04g0339400	24.56	0.0033	Auxin-induced protein PCNT115
LOC_Os08g07080	3	1.45	Os08g0167800	11.05	1.00E-05	Terpene synthase 10
LOC_Os07g34260	2.35	−0.05	Os07g0526400	25.05	< 1.00E-05	Chalcone synthase DII
LOC_Os08g33740	2.55	0.7	Os08g0434500	7.03	0.001	CSLA11 - cellulose synthase-like family A
LOC_Os07g22930	2.1	0.45	Os07g0412100	0.14	0.0221	Granule-bound starch synthase 1b, chloroplast precursor
LOC_Os09g02270	1.8	3.2	Os09g0110300	12.26	< 1.00E-05	Cyclase
LOC_Os05g10310	−1.3	−0.4	Os05g0191500	5.98	0.0045	Acid phosphatase
LOC_Os04g31120	−1.2	−0.55	Os04g0380300	4.59	0.0217	Influenza virus NS1A binding protein isoform 3; kelch repeat-containing F-box family protein
LOC_Os04g58280	−1.3	−0.3	Os04g0679400	6.58	0.002	Stem-specific protein TSJT1
LOC_Os03g16950	−1.4	−0.95	Os03g0277600	7.9	0.0004	Serine/threonine kinase-like protein
LOC_Os01g32670	0.8	−2.45	Os01g0510200	14.77	1.00E-05	Expressed protein
LOC_Os01g32460	2	0.7	Os01g0508500	54.14	< 1.00E-05	Expressed protein
LOC_Os08g04630	1.05	0.55	Os08g0141400	4.24	0.0348	Expressed protein
LOC_Os05g30500	2.55	1.9	Os05g0368000	57.52	< 1.00E-05	Expressed protein
LOC_Os05g38940	1.75	2.15	Os05g0465000	4.6	0.0214	Hypothetical protein
LOC_Os01g40290	0.65	−3.2	Os01g0585200	13.02	< 1.00E-05	Expressed protein
LOC_Os04g49370	−1.9	0.2	Os04g0583200	5.73	0.0052	Expressed protein
LOC_Os04g54210	−1.8	0.35	Os04g0634800	4.99	0.0117	Expressed protein

**Figure 4:**
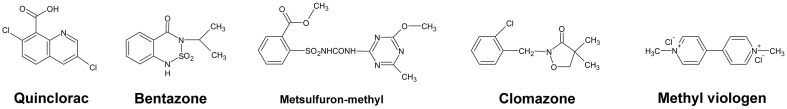
**The chemical structures of five herbicides**.

**Figure 5:**
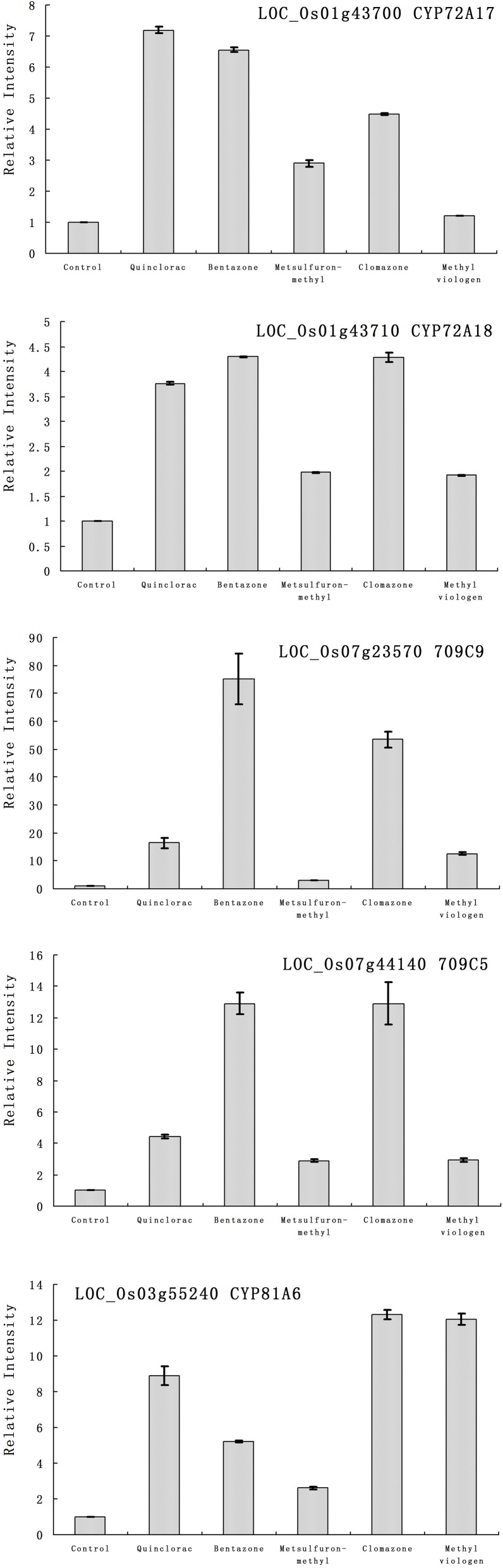
**The expression pattern of selected rice cytochrome P450 genes responded to different chemical treatments**. The expression values were detected by real-time RT-PCR. The gray bars indicated the fold change of the gene between chemical treatment and mock; the error bars represent the standard error of three replicates. LOC_Os01g43700—CYP72A17; LOC_Os01g43710—CYP72A18; LOC_Os03g55240—CYP81A6; LOC_Os07g23570—CYP709C9; LOC_Os07g44140—CYP709C5.

Furthermore, we studied the responses of the five highlighted rice cytochrome P450 genes under different herbicide treatments. We selected several herbicides including quinclorac, bentazone, metsulfuron-methyl, clomazone, and methyl viologen (Figure 4). Clomazone is an herbicide that kills susceptible species by blocking pigment synthesis (Norman et al., 1990a,b). Metsulfuron-methyl is used to control select broadleaf weeds and some annual grasses (Sanyal et al., 2006). Methyl viologen (paraquat) induces oxidative stresses in plants (Tsang et al., 1991; Beligni and Lamattina, 1999; Cummins et al., 1999; Lim et al., 2008). We used real-time RT-PCR to analyze the expression pattern of the five rice cytochrome P450 genes under the selected herbicide treatments. CYP709C5 and CYP709C9 were induced at 6 h of treatment for all the herbicides and highly induced by bentazone and clomazone (Figure 5). CYP72A17 and CYP72A18 were induced by quinclorac, bentazone, and clomazone, whereas CYP81A6 was induced by all herbicides and highly induced by clomazone and methyl viologen (Figure 5).

### Cross-species analysis for the expression patterns of quinclorac up-regulated P450 genes between rice and arabidopsis

**Figure 6:**
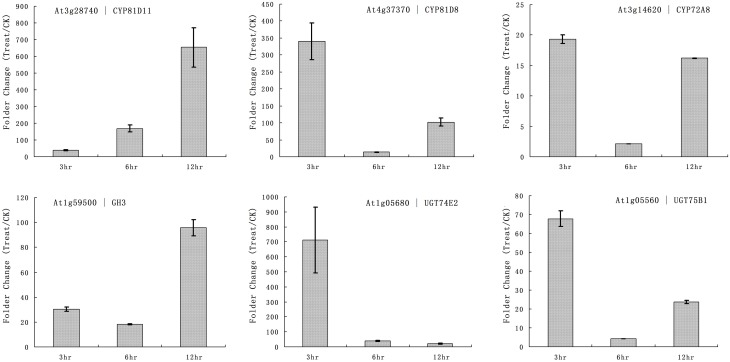
**The expression pattern of selected Arabidopsis genes responded to different times of quinclorac treatment**. The expression values were detected by real-time RT-PCR. The gray bars indicated the fold change of the gene between quinclorac treatment and mock; the error bars represent the standard error of three replicates. At3g28740—CYP81D11; At4g37370—CYP81D8; At3g14620—CYP72A8; At1g59500—GH3; At1g05680—UGT74E2; At1g05560—UGT75B1.

The primary function of P450 is detoxification of xenobiotics, and they utilize herbicides as substrates. Our microarray results showed that multiple rice P450s were responsible for the metabolism of quincorac. We selected several members of the cytochrome P450 family in Arabidopsis (CYP72 and CYP81) and studied their responses to quinclorac treatment using real-time RT-PCR. Three Arabidopsis cytochrome P450 genes were induced by quinclorac treatment; CYP81D11 increased from 3 to 12 h treatment; CYP81D8 and CYP72A8 were highly induced at 3 and 12 h but slightly decreased at 6 h of treatment (Figure 6). As marker genes, GH3 was up-regulated at 12 h, and UGT74E2 and UGT75B1 were much more induced at 3 h by the quinclorac treatment. The Arabidopsis CYP81D11 and CYP81D8 are possible orthologs/homologs of rice CYP81A6, and Arabidopsis CYP72A8 is a possible ortholog/homolog of members of the rice CYP72A subfamily. Therefore, some specific members of CYP81 and CYP72 responded to quinclorac both in the dicot Arabidopsis and the monocot rice. They may play critical roles for detoxification of and resistance to quinclorac.

In summary, microarray analysis indicated that quinclorac showed an auxin-like nature, inducing rice auxin response genes and affecting expression patterns of genes related to other hormones. The GO analysis showed that the detoxification genes including P450, GST, UGT, ABC, and other drug transporter genes were up-regulated and significantly enriched among all quinclorac response genes. These gene families are strongly related to herbicide resistance and detoxification. Real-time RT-PCR results showed that some members of the P450 gene family (CYP81, CYP709C, and CYP72A) were universally induced by different herbicides and also showed up-regulation both in rice and Arabidopsis. Interestingly, some rice receptor-like kinase genes and ubiquitination-related genes were down-regulated under quinclorac treatment. This may open discussion concerning the regulation of quinclorac-involved signal transduction pathways in rice plants. We believe it will be very important for further explanation of the selectivity of quincorac in resistant rice plants and susceptible grasses and dicot weeds. In the meanwhile, the discovery of some key family genes for herbicide resistance will be beneficial to produce transgenic plants to monitor some environmental pollution caused by herbicides; for example, CYP81A6 is already applied for rendering transgenic rice sensitive to bentazone (Lin et al., 2008). Glutathione transferases functioned as glutathione peroxidases in resistance to multiple herbicides in black-grass (Cummins et al., 1999). Furthermore, the detoxification-related genes such as P450s and GSTs may play important roles in protection from herbicide-caused soil and water pollution and improve worldwide environmental health.

## Funding

This work was supported by grants from the Ministry of Science and Technology of China (Grant Numbers: 31371291 and 30570139) and the Ministry of Education of China (Grant Numbers: NCET-09-0735).

### Conflict of interest statement

The authors declare that the research was conducted in the absence of any commercial or financial relationships that could be construed as a potential conflict of interest.

## Supplementary material

Data Sheet 1 **The raw chip data for the rice expression profilings under quinclorac treatment**.

Supplemental Figure 1 **The GO category (molecular function) enrichment analysis result of the up-regulated probe sets under quinclorac treatment for 6 and 24 h**.

Supplemental Figure 2 **The GO category (biological process) enrichment analysis result of the up-regulated probe sets under quinclorac treatment for 6 and 24 h**.

Supplemental Figure 3 **The significant enriched BINs of the up-regulated probe sets under quinclorac treatment for 6 h (top) and 24 h (bottom) through GeneBins analysis**.

Supplemental Figure 4 **The quinclorac-responsive genes for 6 h (left) and 24 h (right) of treatment related to regulation categories**. The colored squares represent the expression change of probe sets in log_2_ ratio, with red indicating the gene induced by quinclorac treatment and the blue indicating the gene reduced by quinclorac treatment, detailed range is shown as a colored bar.

Supplemental Table 1 **List of 637 probe sets responed to quinclorac treatment in rice**.

Supplemental Table 2 **List of primer sequences used in real-time RT-PCR**.
