# Supplementary figures and images for: Rice transcriptome analysis to identify possible herbicide quinclorac detoxification genes
Source: Front Genet. 2015 Sep 29;6:306. doi: 10.3389/fgene.2015.00306 (PMC4586585; doi:10.3389/fgene.2015.00306)

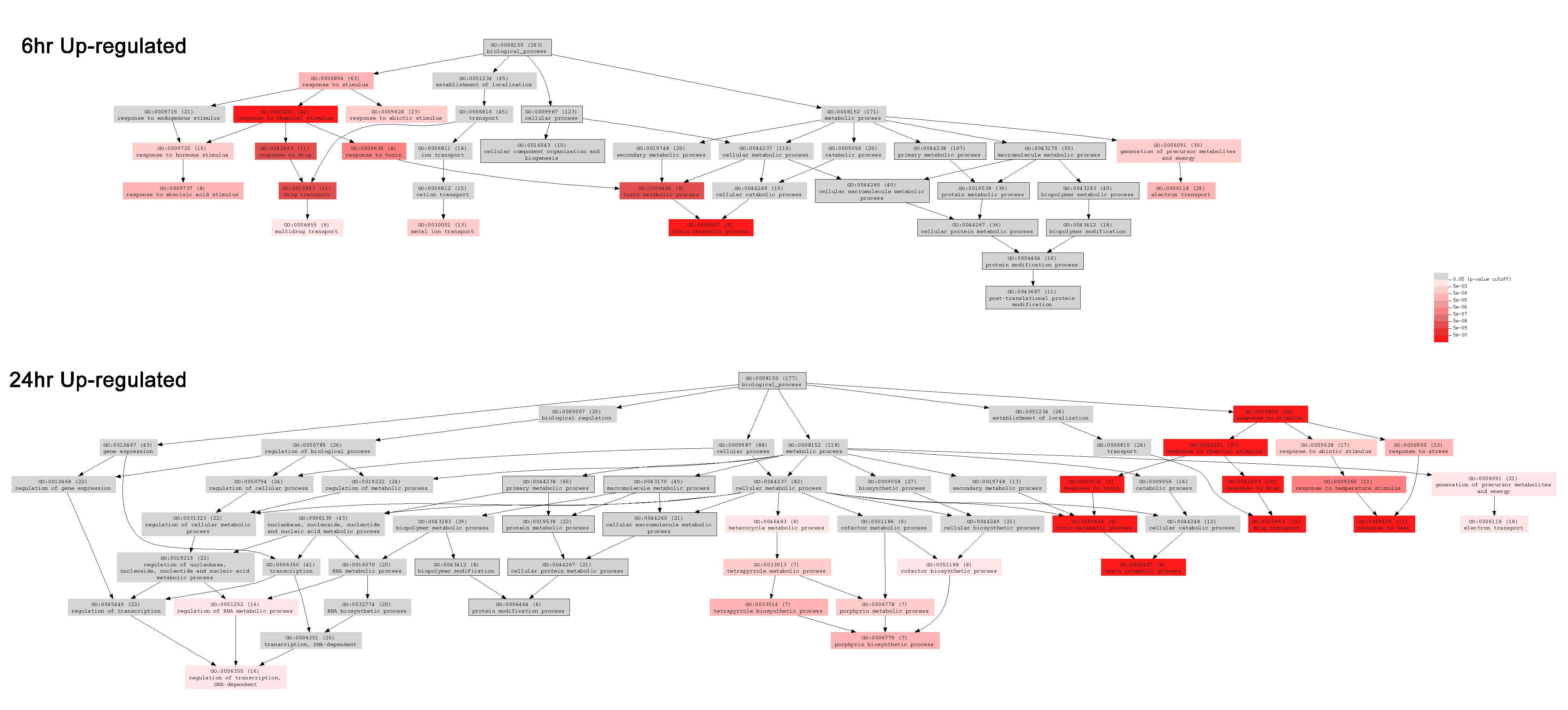

Supplement: Supplemental Figure 2 — The GO category (biological process) enrichment analysis result of the up-regulated probe sets under quinclorac treatment for 6 and 24 h. [file Image2.PNG]

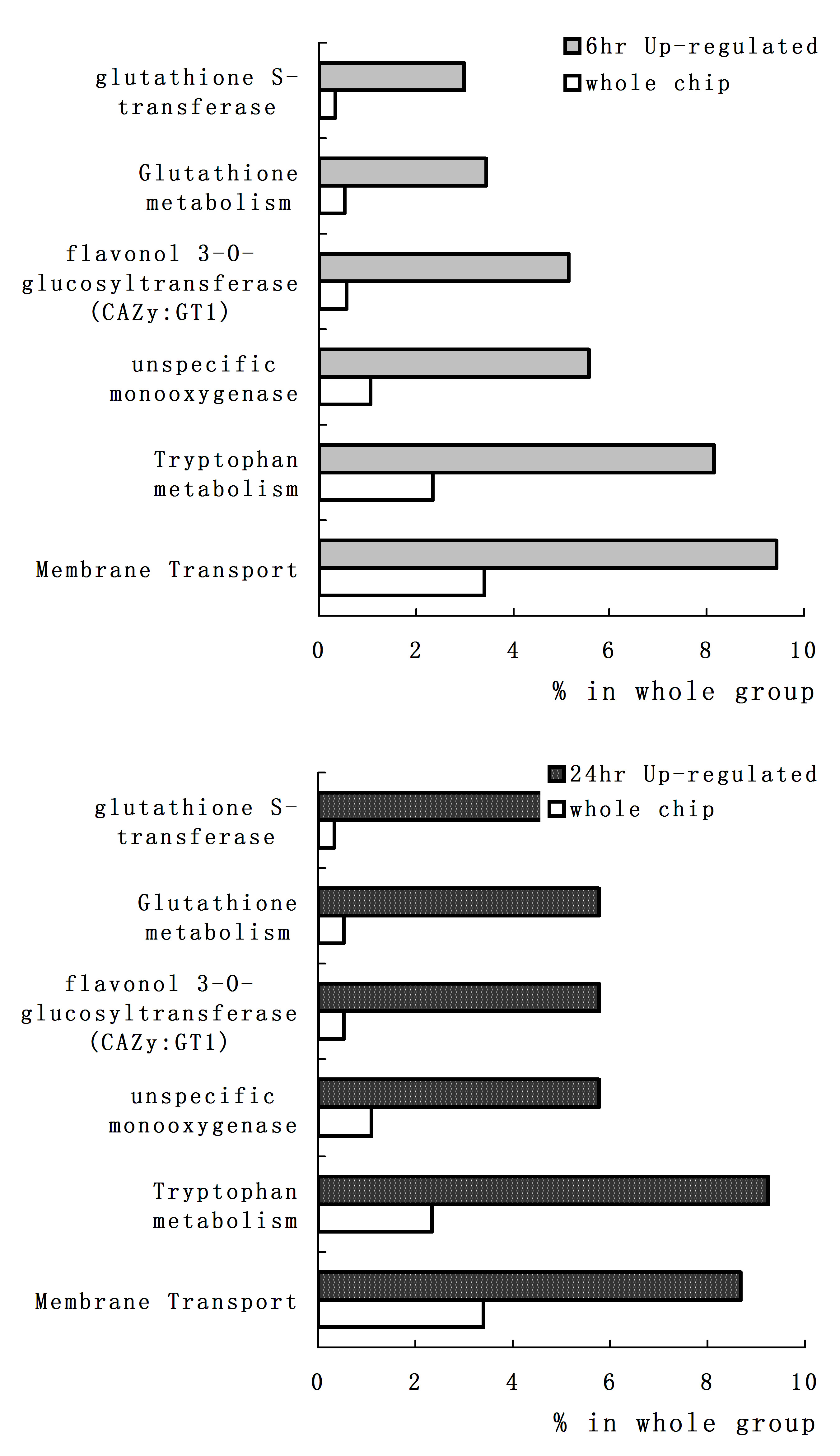

Supplement: Supplemental Figure 3 — The significant enriched BINs of the up-regulated probe sets under quinclorac treatment for 6 h (top) and 24 h (bottom) through GeneBins analysis. [file Image3.JPEG]
