# Supplementary material for: Rice transcriptome analysis to identify possible herbicide quinclorac detoxification genes
Source: Front Genet. 2015 Sep 29;6:306. doi: 10.3389/fgene.2015.00306 (PMC4586585; doi:10.3389/fgene.2015.00306)
Supplement: Supplemental Table 1 — List of 637 probe sets responed to quinclorac treatment in rice. [file Table1.PDF]

Supplemetal Table 1. List of 637 probe sets responed to quinclorac treatment in rice

Probe Set ID/Locus ID	Time	Quinclorac vs. Mock Repeat 1				Quinclorac vs. Mock Repeat 2				Assignment	Description
		Quinclorac	Mock	Log Ratio	P-value	Quinclorac	Mock	Log Ratio	P-value		
Os.10546.1.S1_s_at	6hr	5405.4	1482.8	1.90	2.00E-05	4657.8	679.9	2.60	2.00E-05	Up-regulated	indole-3-acetate beta-glucosyltransferase
LOC_Os09g34230	24hr	4955.2	1124.1	2.20	2.00E-05	4441.2	1384.9	1.60	2.00E-05	Up-regulated	
Os.10570.1.S1_at	6hr	233.4	82.2	1.70	3.00E-05	251.6	105.9	1.30	7.80E-05	Up-regulated	heat shock factor protein 3
LOC_Os06g35960	24hr	269.1	52.7	1.60	1.89E-04	350	46.7	2.10	8.80E-05	Up-regulated	
Os.11193.1.S1_at	6hr	7120.5	2199.3	1.60	2.00E-05	9584	3332.5	1.60	2.00E-05	Up-regulated	cytochrome P450 81A6
LOC_Os03g55240	24hr	9312.2	1650.6	2.10	2.00E-05	15579.3	1555	3.20	2.00E-05	Up-regulated	
Os.11218.1.S1_at	6hr	2558.9	632.5	1.90	2.00E-05	3757.6	282.9	3.70	2.00E-05	Up-regulated	peroxiredoxin
LOC_Os07g44440	24hr	3362.5	544.7	2.60	2.00E-05	12325.7	458.8	4.50	2.00E-05	Up-regulated	
Os.11549.1.S1_at	6hr	556.8	271.6	1.20	3.50E-05	488.8	224.5	1.50	2.30E-05	Up-regulated	peroxidase 52 precursor
LOC_Os12g02080	24hr	415.6	139.5	1.30	2.00E-05	338.3	153.1	1.20	6.00E-05	Up-regulated	
Os.11632.1.S1_at	6hr	15847	7177.6	1.10	2.00E-05	18464.4	7858.6	1.20	2.00E-05	Up-regulated	mtN19-like protein
LOC_Os08g42590	24hr	18337.3	5769.2	1.60	2.00E-05	23352.7	6409.4	1.90	2.00E-05	Up-regulated	
Os.11812.1.S1_at	6hr	834	86.3	3.60	2.00E-05	661.8	83	3.20	2.00E-05	Up-regulated	jasmonate O-methyltransferase
LOC_Os02g48770	24hr	202.2	24.9	3.20	2.00E-05	339.8	18.4	3.70	2.00E-05	Up-regulated	
Os.11837.1.S1_at	6hr	11719.9	6635.7	1.10	2.00E-05	13121.4	7483.4	1.00	2.00E-05	Up-regulated	IN2-2 protein
LOC_Os04g26920	24hr	12239.4	5226.1	1.30	2.00E-05	13568.3	6524.6	1.30	2.00E-05	Up-regulated	
Os.12165.1.S1_at	6hr	5936.8	1410.7	2.00	2.00E-05	4749.2	827.5	2.10	2.00E-05	Up-regulated	OsAPRL1 - Oryza sativa adenosine 5'-phosphosulfate
LOC_Os07g32570	24hr	4289.5	2126.7	1.00	2.00E-05	6549.1	1751.1	1.80	2.00E-05	Up-regulated	reductase-like
Os.12200.1.S1_s_at	6hr	897.3	310.3	2.10	2.00E-05	2193.1	440.1	2.90	2.00E-05	Up-regulated	glutathione S-transferase parA
LOC_Os03g57200	24hr	3244.7	329.3	4.10	2.00E-05	6785.5	486.6	4.00	2.00E-05	Up-regulated	
Os.12253.1.S1_at	6hr	1109.1	374.1	1.50	4.00E-05	1381.8	285.4	2.20	2.00E-05	Up-regulated	cytokinin-O-glucosyltransferase 1
LOC_Os01g45110	24hr	1435.9	189.8	2.60	2.00E-05	3134.4	221.9	3.50	2.00E-05	Up-regulated	
Os.12363.1.S1_at	6hr	1416.1	712.8	1.10	2.00E-05	1235.9	378.4	1.70	2.00E-05	Up-regulated	protochlorophyllide reductase A, chloroplast
LOC_Os04g58200	24hr	3061.7	433	3.20	2.00E-05	983.7	468.8	1.10	3.50E-05	Up-regulated	precursor
Os.12501.1.S1_at	6hr	3608.5	403.8	3.20	2.00E-05	4009.7	380	3.20	2.00E-05	Up-regulated	indole-3-acetic acid-amido synthetase GH3.2
LOC_Os01g55940	24hr	3147.6	382.5	2.80	2.00E-05	7843.5	272	4.80	2.00E-05	Up-regulated	
Os.14631.1.S1_at	6hr	2344	860.4	1.40	3.00E-05	3006.5	1356.2	1.20	6.00E-05	Up-regulated	cytokinin-N-glucosyltransferase 1
LOC_Os07g13770	24hr	2945.3	715.7	1.80	3.50E-05	4758.8	1448.9	1.70	2.00E-05	Up-regulated	
Os.15281.1.S1_at	6hr	530.3	106.1	2.80	1.47E-04	848.8	139.4	3.10	2.00E-05	Up-regulated	sex determination protein tasselseed-2
LOC_Os07g46920	24hr	618.3	258.3	1.70	2.30E-05	1369	286.5	3.20	2.30E-05	Up-regulated	
Os.15281.1.S1_x_at	6hr	581.4	106.3	2.40	1.08E-03	856.2	154.4	3.20	2.73E-04	Up-regulated	sex determination protein tasselseed-2
LOC_Os07g46920	24hr	593	268.4	1.60	1.67E-04	1410.4	256.7	2.90	5.20E-05	Up-regulated	
Os.15699.1.S1_at	6hr	321.8	184.4	1.40	3.04E-03	544.2	149.7	1.90	7.74E-04	Up-regulated	glutathione S-transferase GSTU6
LOC_Os10g38590	24hr	306.4	194.2	1.00	7.80E-05	558.7	108.4	2.40	1.67E-04	Up-regulated	
Os.17865.1.S1_at	6hr	1027.3	255.8	1.80	2.00E-05	1495.9	235.8	2.50	2.00E-05	Up-regulated	transposon protein
LOC_Os10g36980	24hr	924	223.4	2.40	2.00E-05	1439.1	111	3.60	2.00E-05	Up-regulated	
Os.18177.1.S1_at	6hr	730.9	316.6	1.30	2.00E-05	929.7	321.8	1.50	2.00E-05	Up-regulated	carbohydrate transporter/ sugar porter/
LOC_Os11g04104	24hr	912.8	378.6	1.30	2.00E-05	1564.5	413	1.90	2.00E-05	Up-regulated	transporter
Os.18592.1.S1_at	6hr	158.9	89.8	1.00	1.47E-04	134.1	69.7	1.30	4.92E-04	Up-regulated	cytokinin-O-glucosyltransferase 2
LOC_Os07g13780	24hr	214.4	57.8	2.30	2.00E-05	121.9	94	1.20	1.31E-02	Up-regulated	
Os.20154.1.S1_at	6hr	292.9	50.8	1.80	8.65E-04	427.4	21.3	4.00	2.30E-05	Up-regulated	dihydroflavonol-4-reductase
LOC_Os02g56700	24hr	725.4	12.7	4.70	2.30E-05	625.6	6.5	5.90	6.80E-05	Up-regulated	
Os.21103.1.S1_x_at	6hr	7414.8	3291.6	1.00	2.00E-05	7701.9	3074.9	1.20	2.00E-05	Up-regulated	gibberellin 20 oxidase 2
LOC_Os03g42130	24hr	10870.4	4057.2	1.20	2.00E-05	12208.7	4992.9	1.30	2.00E-05	Up-regulated	
Os.21103.2.S1_at	6hr	567.8	211.6	1.30	2.30E-05	540.8	191.9	1.40	5.20E-05	Up-regulated	gibberellin 20 oxidase 2
LOC_Os03g42130	24hr	741.6	261.2	1.40	2.00E-05	725.1	185.2	1.90	3.00E-05	Up-regulated	
Os.23397.1.S1_at	6hr	1719	1005.7	1.00	2.00E-05	1818	920.3	1.30	2.00E-05	Up-regulated	oxidoreductase
LOC_Os08g37874	24hr	1633.2	672.4	1.40	2.00E-05	2208	585.8	2.30	2.00E-05	Up-regulated	
Os.24993.1.A1_s_at	6hr	1154.9	568.6	1.10	2.00E-05	1300.1	538	1.30	2.00E-05	Up-regulated	thiosulfate transferase
LOC_Os02g07044	24hr	1975.9	706.4	1.50	2.00E-05	2822.5	635.2	2.10	2.00E-05	Up-regulated	

Probe Set ID/Locus ID	Time	Quinclorac vs. Mock Repeat 1				Quinclorac vs. Mock Repeat 2				Assignment	Description
		Quinclorac	Mock	Log Ratio	P-value	Quinclorac	Mock	Log Ratio	P-value		
Os.25606.1.S1_at	6hr	664.2	180.8	2.20	6.00E-05	463.3	149.5	2.20	6.00E-05	Up-regulated	OsWRKY76 - Superfamily of rice TFs having WRKY and
LOC_Os09g25060	24hr	807.3	236.7	2.20	8.80E-05	397.6	172.8	1.00	4.60E-05	Up-regulated	zinc finger domains
Os.2612.1.S1_at	6hr	1269.9	366.7	2.00	3.50E-05	3004.4	405.1	3.00	2.00E-05	Up-regulated	glutathione S-transferase GSTU6
LOC_Os10g38495	24hr	2208.9	270.8	2.40	2.00E-05	4891.5	317.5	4.00	2.00E-05	Up-regulated	
Os.26820.1.A1_s_at	6hr	274.1	4.2	5.90	2.00E-05	194.6	2.7	4.90	8.80E-05	Up-regulated	terpene synthase 10
LOC_Os08g07080	24hr	199.4	64.2	1.40	2.30E-05	1084.6	284.5	2.00	2.00E-05	Up-regulated	
Os.32668.1.S1_at	6hr	894.4	302.1	1.30	2.70E-05	1450.7	485.9	1.60	2.00E-05	Up-regulated	flavonol-3-O-glycoside-7-O-glucosyltransferase 1
LOC_Os01g08090	24hr	1029.3	228.8	2.20	2.00E-05	1448.9	241.6	2.60	2.00E-05	Up-regulated	
Os.37565.2.S1_at	6hr	2247.8	513.3	1.60	2.00E-05	1001	438.4	1.40	6.80E-05	Up-regulated	OsWRKY45 - Superfamily of rice TFs having WRKY and
LOC_Os05g25770	24hr	1500.5	571.4	1.00	6.80E-05	421.7	186.2	1.00	2.73E-04	Up-regulated	zinc finger domains
Os.39973.1.S1_s_at	6hr	3603.8	1178.9	1.60	2.00E-05	2238.4	864.6	1.40	2.00E-05	Up-regulated	actin-depolymerizing factor 4
LOC_Os03g60580	24hr	3068.2	1154.6	1.50	2.00E-05	6621	843.1	2.90	2.00E-05	Up-regulated	
Os.4291.1.S1_at	6hr	17094.3	7064.2	1.20	2.00E-05	15668	6769.3	1.20	2.00E-05	Up-regulated	cytochrome P450 704A4
LOC_Os06g03930	24hr	17646.5	6509.7	1.70	2.00E-05	23055.4	5414.2	2.20	2.00E-05	Up-regulated	
Os.43896.1.S1_at	6hr	151.2	41.3	1.70	3.00E-05	166.4	42.5	2.20	3.07E-04	Up-regulated	transparent testa 12 protein
LOC_Os03g08900	24hr	460.6	64.5	2.70	2.00E-05	570.8	92.4	2.80	6.00E-05	Up-regulated	
Os.4762.1.S1_at	6hr	5323.1	1942.7	1.50	2.00E-05	11518.3	2550.2	2.20	2.00E-05	Up-regulated	glutathione S-transferase GSTU6
LOC_Os10g38740	24hr	3884.3	828.3	2.30	2.00E-05	6076.2	828.1	3.00	2.00E-05	Up-regulated	
Os.48216.1.S1_at	6hr	5923.1	208.8	5.20	2.00E-05	7921.2	151.2	5.90	2.00E-05	Up-regulated	indole-3-acetate beta-glucosyltransferase
LOC_Os09g34250	24hr	11102.8	245.6	5.40	2.00E-05	17561.9	187.5	6.30	2.00E-05	Up-regulated	
Os.4863.1.S1_at	6hr	3726.6	1174.1	1.70	2.00E-05	3717.4	1095.6	1.60	2.00E-05	Up-regulated	siroheme synthase
LOC_Os01g44050	24hr	5133.3	1141.5	2.20	2.00E-05	5445.2	1752.6	1.80	2.00E-05	Up-regulated	
Os.4863.2.S1_at	6hr	928.3	276.1	1.70	3.00E-05	1004.6	257.2	1.90	2.00E-05	Up-regulated	siroheme synthase
LOC_Os01g44050	24hr	1747.8	324.5	2.40	3.00E-05	1757.1	412	2.00	2.00E-05	Up-regulated	
Os.49030.1.A1_s_at	6hr	819.8	79.8	3.00	3.50E-05	1388.7	92.9	3.60	2.70E-05	Up-regulated	glutathione S-transferase
LOC_Os09g20220	24hr	1733.3	74.1	4.30	2.70E-05	6056.9	81.1	6.20	2.00E-05	Up-regulated	
Os.49245.1.S1_at	6hr	735.4	114.9	2.70	2.00E-05	375.2	86.6	2.00	2.00E-05	Up-regulated	homeobox-leucine zipper protein ATHB-6
LOC_Os02g43330	24hr	240.8	49.4	1.40	1.14E-04	369.6	44	2.40	2.30E-05	Up-regulated	
Os.51574.1.S1_at	6hr	403.9	205.3	1.50	5.52E-04	445	195.3	1.30	2.00E-05	Up-regulated	expressed protein
LOC_Os04g56390	24hr	519.2	234.9	1.20	6.80E-05	750.1	271	1.50	1.14E-04	Up-regulated	
Os.5367.1.S1_at	6hr	1500.7	652.6	1.70	2.00E-05	1116.4	369.8	1.50	4.93E-03	Up-regulated	protein phosphatase 2C ABI2
LOC_Os01g40094	24hr	955.7	494.3	1.30	5.20E-05	1339.6	543.7	1.30	7.80E-05	Up-regulated	
Os.54037.1.S1_s_at	6hr	635.3	283.3	1.10	2.00E-05	771.4	363.2	1.00	2.70E-05	Up-regulated	expressed protein
LOC_Os02g15700	24hr	991.1	308.7	2.10	2.70E-05	1577.4	269.8	2.30	2.00E-05	Up-regulated	
Os.54482.1.S1_at	6hr	207.5	38.4	1.90	3.00E-05	193.6	53.8	1.50	2.30E-05	Up-regulated	ATP binding protein
LOC_Os02g32690	24hr	259.3	82.3	1.80	2.00E-05	322.4	95.4	2.00	2.00E-05	Up-regulated	
Os.55250.1.S1_at	6hr	731.4	107	3.20	2.00E-05	1922.4	193.5	3.20	2.00E-05	Up-regulated	TMV response-related gene product
LOC_Os09g32000	24hr	1670.6	111.5	3.70	2.00E-05	1254.7	159.8	4.00	2.00E-05	Up-regulated	
Os.55787.1.S1_at	6hr	269.2	93.8	1.60	2.00E-05	282.3	103.1	1.80	7.80E-05	Up-regulated	glutathione reductase, chloroplast precursor
LOC_Os10g28000	24hr	199.1	79.5	1.60	3.50E-05	223.4	47.2	2.30	1.67E-04	Up-regulated	
Os.57316.1.S1_at	6hr	509.5	219.2	1.60	2.00E-05	664	188.9	1.90	2.00E-05	Up-regulated	hypothetical protein
LOC_Os05g38940	24hr	255	89.8	1.40	2.00E-05	609.4	101.9	2.90	2.00E-05	Up-regulated	
Os.5794.1.S1_a_at	6hr	283.9	111.1	1.40	2.30E-05	310.3	162.4	1.20	1.14E-04	Up-regulated	transposon protein, Mutator sub-class
LOC_Os03g10800	24hr	613.8	163.4	1.70	2.00E-05	780.2	124.9	2.20	2.00E-05	Up-regulated	
Os.6157.1.S1_at	6hr	717.2	380.7	1.10	2.00E-05	863.1	403.2	1.20	2.00E-05	Up-regulated	esterase PIR7B
LOC_Os01g37630	24hr	1043	402.3	1.30	6.18E-04	1461.6	379.6	2.00	2.30E-05	Up-regulated	
Os.6452.2.A1_a_at	6hr	8811.5	3316.1	1.40	2.00E-05	7443.4	2328.3	1.50	2.00E-05	Up-regulated	cytochrome P450 72A18
LOC_Os01g43710	24hr	7636.7	2851.6	1.40	2.00E-05	8781.9	2014.4	2.00	2.00E-05	Up-regulated	
Os.6517.1.S1_at	6hr	1685.1	633.1	1.20	2.00E-05	1335	501.8	1.30	2.00E-05	Up-regulated	transparent testa 12 protein
LOC_Os08g37432	24hr	2499.2	1141.5	1.00	2.00E-05	1628.6	897.9	1.20	2.30E-05	Up-regulated	
Os.6863.1.S1_at	6hr	1413.3	428.3	2.00	2.00E-05	824.7	253.2	1.70	2.00E-05	Up-regulated	jasmonate-induced protein
LOC_Os12g14440	24hr	21	4.3	2.00	5.00E-01	1478.8	171.3	3.40	2.00E-05	Up-regulated	
Os.6998.1.S1_at	6hr	3217.5	1269.9	1.30	2.00E-05	3274.9	965.5	1.70	2.00E-05	Up-regulated	transparent testa 12 protein

Probe Set ID/Locus ID	Time	Quinclorac vs. Mock Repeat 1				Quinclorac vs. Mock Repeat 2				Assignment	Description
		Quinclorac	Mock	Log Ratio	P-value	Quinclorac	Mock	Log Ratio	P-value		
LOC_Os03g37490	24hr	3318.3	875.4	2.00	2.00E-05	4341.5	747.6	2.70	2.00E-05	Up-regulated	
Os.7539.1.S1_at	6hr	621	214	1.60	2.00E-05	681.2	226.6	1.70	2.00E-05	Up-regulated	expressed protein
LOC_Os03g03320	24hr	687.4	317.8	1.00	2.00E-05	1619.2	411.6	1.70	2.00E-05	Up-regulated	
Os.767.1.S1_at	6hr	1099.7	295.9	2.20	3.00E-05	1320.7	152.4	3.00	2.00E-05	Up-regulated	cytochrome P450 72A17
LOC_Os01g43700	24hr	1943.1	429.8	2.50	2.00E-05	2640	551.1	3.00	2.00E-05	Up-regulated	
Os.7806.1.S1_at	6hr	2905.6	938.8	1.70	2.00E-05	5338.4	1056.5	1.70	4.60E-05	Up-regulated	gibberellin 20 oxidase 2
LOC_Os04g39980	24hr	6730.6	1998	1.50	2.00E-05	11547.1	1656.5	2.10	2.00E-05	Up-regulated	
Os.7911.1.S1_at	6hr	6588.3	3008.3	1.10	2.00E-05	9817	3160.3	1.50	2.00E-05	Up-regulated	glutathione S-transferase IV
LOC_Os01g27210	24hr	7060.3	2336.2	1.60	2.00E-05	10516.6	1849.6	2.40	2.00E-05	Up-regulated	
Os.8902.1.S1_at	6hr	560.8	76.4	2.50	6.00E-05	688.6	36.3	4.00	3.50E-05	Up-regulated	transferase
LOC_Os02g28220	24hr	1890.8	117.9	4.00	2.00E-05	2584.3	58	4.70	2.00E-05	Up-regulated	
Os.8947.1.S1_at	6hr	599.9	132.8	1.90	2.00E-05	1118.5	190.5	2.50	2.00E-05	Up-regulated	monooxygenase
LOC_Os03g05910	24hr	1940.6	179.2	3.50	2.00E-05	4675	160.8	4.90	2.00E-05	Up-regulated	
Os.9017.1.S1_x_at	6hr	1719.8	238.3	3.00	2.00E-05	2292.9	161.2	4.10	2.00E-05	Up-regulated	cytochrome P450 709C9
LOC_Os07g23570	24hr	1156.2	43.9	5.20	2.00E-05	2182.8	10.6	9.20	2.00E-05	Up-regulated	
Os.9067.1.S1_at	6hr	1446.2	655.7	1.70	2.00E-05	2016.2	439.4	2.60	2.00E-05	Up-regulated	cytochrome P450 709C5
LOC_Os07g44140	24hr	4748	328.8	3.50	2.00E-05	3761.3	263.5	4.00	2.00E-05	Up-regulated	
Os.9101.1.S1_at	6hr	3778.2	1591.3	1.10	2.00E-05	5748.8	1905.4	1.50	2.00E-05	Up-regulated	glutathione S-transferase GSTU6
LOC_Os10g38780	24hr	5110.3	1256.1	1.70	2.00E-05	5208.1	1554.6	1.80	2.00E-05	Up-regulated	
Os.9105.1.S1_at	6hr	6341.9	1517.6	2.00	2.00E-05	6549.1	1095.1	2.40	2.00E-05	Up-regulated	cyanogenic beta-glucosidase precursor
LOC_Os09g33680	24hr	12788.3	4816.1	1.40	2.00E-05	15955.5	3823.5	2.00	2.00E-05	Up-regulated	
Os.9752.1.S1_a_at	6hr	241.5	134.3	1.00	2.14E-04	380.4	141.4	1.50	1.14E-04	Up-regulated	carbohydrate transporter/ sugar porter/ transporter
LOC_Os12g03899	24hr	418.6	144.9	1.20	3.50E-05	1044.9	235.3	2.20	2.00E-05	Up-regulated	
Os.9752.2.S1_x_at	6hr	2425.7	774.3	1.40	2.00E-05	1942.5	796.1	1.40	2.00E-05	Up-regulated	carbohydrate transporter/ sugar porter/ transporter
LOC_Os12g03899	24hr	2842.4	1295	1.20	2.00E-05	5683.6	1306.4	1.90	2.00E-05	Up-regulated	
Os.9752.3.S1_x_at	6hr	2024.5	1004	1.30	2.00E-05	1876	861.7	1.40	2.00E-05	Up-regulated	carbohydrate transporter/ sugar porter/ transporter
LOC_Os11g04104	24hr	2902.2	1383.5	1.20	2.00E-05	5181.8	1337.9	1.90	2.00E-05	Up-regulated	
Os.9776.1.S1_a_at	6hr	1410.4	694.2	1.10	2.00E-05	1674.2	664.3	1.20	2.00E-05	Up-regulated	leucoanthocyanidin dioxygenase
LOC_Os03g32470	24hr	1455.7	720	1.00	2.00E-05	2243.1	1143.6	1.20	2.00E-05	Up-regulated	
Os.9823.1.S1_at	6hr	1283.3	421.6	1.50	2.00E-05	2011.5	349.7	2.10	2.00E-05	Up-regulated	cyclase
LOC_Os09g02270	24hr	4737.5	535.2	2.90	2.00E-05	6335.4	508.4	3.50	2.00E-05	Up-regulated	
OsAffx.11989.2.S1_s_at	6hr	521.6	192	1.70	1.47E-04	460.2	200.1	1.30	2.00E-05	Up-regulated	cytokinin-O-glucosyltransferase 3
LOC_Os02g11700	24hr	2203.9	1062.6	1.10	2.00E-05	4372.5	761.6	2.10	2.00E-05	Up-regulated	
OsAffx.12799.1.S1_s_at	6hr	1221.5	47.9	3.40	2.00E-05	524.1	100.6	2.20	2.73E-04	Up-regulated	DRE binding factor
LOC_Os03g09170	24hr	192	69.3	1.10	1.01E-04	155.5	35.4	1.80	2.75E-03	Up-regulated	
OsAffx.14888.1.S1_at	6hr	1317.9	205.1	2.70	2.00E-05	1124.7	181.9	2.40	2.00E-05	Up-regulated	expressed protein
LOC_Os05g30500	24hr	852.6	158.5	2.20	2.00E-05	436.3	123	1.60	1.89E-04	Up-regulated	
OsAffx.19165.1.S1_at	6hr	242.1	92.8	1.70	1.67E-04	487.3	72.4	2.70	2.00E-05	Up-regulated	tyrosine aminotransferase
LOC_Os11g35040	24hr	274.2	36.9	2.60	2.00E-05	440.6	71.3	2.70	2.00E-05	Up-regulated	
OsAffx.24543.1.S1_at	6hr	299.3	25.5	3.50	3.46E-04	344.1	32.2	3.60	4.38E-04	Up-regulated	ATP binding protein
LOC_Os02g32690	24hr	644.4	166.1	2.40	2.00E-05	951.9	158.5	2.30	6.00E-05	Up-regulated	
OsAffx.26173.1.S1_s_at	6hr	8108.3	2907.3	1.40	2.00E-05	7453.7	3073	1.40	2.00E-05	Up-regulated	IN2-2 protein
LOC_Os04g26920	24hr	9034.5	2596.5	1.80	2.00E-05	9073.4	2712.6	1.80	2.00E-05	Up-regulated	
OsAffx.30138.1.S1_at	6hr	3610.2	947.4	1.80	2.00E-05	3108.1	716.1	2.10	2.00E-05	Up-regulated	indole-3-acetate beta-glucosyltransferase
LOC_Os09g34230	24hr	3203.6	565	2.40	2.00E-05	3731	1147.8	1.60	2.00E-05	Up-regulated	
OsAffx.30176.1.S1_at	6hr	855.3	370.9	1.00	2.00E-05	636.4	327.8	1.10	2.00E-05	Up-regulated	NIN-like protein 1
LOC_Os09g37710	24hr	458.2	154.3	1.30	2.00E-05	452.9	237.5	1.00	4.00E-05	Up-regulated	
OsAffx.4180.1.S1_at	6hr	212.4	94.7	1.30	3.00E-05	341.6	48.8	2.70	2.00E-05	Up-regulated	expressed protein
LOC_Os04g55100	24hr	403.3	126.3	1.30	1.67E-04	621.6	93.1	2.70	2.00E-05	Up-regulated	
Os.10097.1.S1_at	6hr	5094.2	1641.3	1.50	2.00E-05	4020.4	1606.4	1.40	2.00E-05	Up-regulated	farnesylated protein 1
LOC_Os04g17100	24hr	5235.8	3747.4	0.40	2.41E-04	8095.8	4259.7	1.00	2.00E-05	Un-changed	
Os.10099.1.S1_at	6hr	1857.8	496.6	2.30	2.00E-05	1224.6	328.8	2.00	2.00E-05	Up-regulated	expressed protein
LOC_Os03g13870	24hr	281	412.4	-0.40	9.29E-03	593.4	443.8	0.50	2.49E-03	Un-changed	

Probe Set ID/Locus ID	Time	Quinclorac vs. Mock Repeat 1				Quinclorac vs. Mock Repeat 2				Assignment	Description
		Quinclorac	Mock	Log Ratio	P-value	Quinclorac	Mock	Log Ratio	P-value		
Os.10300.1.S1_at	6hr	660.4	70.3	3.20	2.00E-05	578.1	76.9	3.00	2.00E-05	Up-regulated	helix-loop-helix DNA-binding domain containing
LOC_Os01g01840	24hr	125.9	147.8	0.00	5.00E-01	391.8	102.7	2.10	2.00E-05	Un-changed	protein
Os.10300.1.S1_x_at	6hr	316.3	70	2.60	2.00E-05	325.2	75.6	2.00	2.00E-05	Up-regulated	helix-loop-helix DNA-binding domain containing
LOC_Os01g01840	24hr	98.8	132.9	-0.30	3.79E-01	322.4	100.1	0.80	4.92E-04	Un-changed	protein
Os.10430.1.S1_at	6hr	336.1	166.9	1.00	2.30E-05	464.9	137.1	1.30	4.38E-04	Up-regulated	cytochrome P450 81E1
LOC_Os03g55260	24hr	659.6	208.5	1.50	2.00E-05	446.1	286.5	0.90	2.30E-05	Un-changed	
Os.10556.1.S1_at	6hr	1111.7	302.6	1.70	2.00E-05	801.9	132.3	2.40	2.00E-05	Up-regulated	expressed protein
LOC_Os02g15860	24hr	1328.8	719.7	0.80	2.00E-05	1447.6	396.7	1.80	2.00E-05	Un-changed	
Os.10617.1.S1_at	6hr	12396.5	4986.5	1.30	2.00E-05	8687.9	2632.9	1.50	2.00E-05	Up-regulated	lysine-specific histone demethylase 1
LOC_Os04g57560	24hr	2643	3541.1	-0.10	5.00E-01	1695.6	2401.5	-0.50	4.00E-05	Un-changed	
Os.10754.1.S1_at	6hr	878.4	375.9	1.30	2.00E-05	492.3	295	1.00	2.00E-05	Up-regulated	sulfate transporter 3.1
LOC_Os03g06520	24hr	771.7	911.1	-0.20	3.79E-01	1175.3	702.9	0.70	2.00E-05	Un-changed	
Os.10901.1.S1_a_at	6hr	3033.3	982.8	1.70	2.00E-05	3155.5	900.2	1.90	2.00E-05	Up-regulated	LHY protein
LOC_Os08g06110	24hr	22845.8	18853.4	0.20	1.81E-02	23386.3	19634.1	0.20	7.12E-03	Un-changed	
Os.11307.1.S1_at	6hr	7978.5	2347.5	1.60	2.00E-05	4429.9	1792.8	1.40	2.00E-05	Up-regulated	expressed protein
LOC_Os06g40440	24hr	928.9	853.6	0.10	3.67E-01	788.3	864.3	-0.20	5.00E-01	Un-changed	
Os.11407.1.S1_at	6hr	3539.5	1171.6	1.40	2.00E-05	2695.3	1056.8	1.40	2.00E-05	Up-regulated	lysine-specific histone demethylase 1
LOC_Os04g57550	24hr	1110.2	1227.2	0.00	5.00E-01	1184.9	1009.4	0.20	6.98E-02	Un-changed	
Os.11575.3.S1_x_at	6hr	204.4	92.3	1.10	2.73E-04	174.2	38.6	2.20	1.30E-04	Up-regulated	regulatory protein NPR1
LOC_Os03g46440	24hr	185.1	124.5	0.90	8.65E-04	246.7	153.8	0.70	5.20E-05	Un-changed	
Os.11770.1.S1_at	6hr	997.6	508.8	1.60	5.20E-05	922.1	399.7	1.10	2.41E-04	Up-regulated	peroxidase 2 precursor
LOC_Os07g48040	24hr	407.2	231.8	0.60	1.34E-03	196.8	198	0.10	1.31E-01	Un-changed	
Os.11800.1.S1_at	6hr	1273.6	608.6	1.00	2.00E-05	2012.8	762.6	1.40	2.00E-05	Up-regulated	multidrug resistance protein 4
LOC_Os01g50100	24hr	229.1	360.1	-0.80	2.14E-04	315.5	292.7	-0.10	5.00E-01	Un-changed	
Os.11800.1.S1_s_at	6hr	2462.1	1222.6	1.10	2.70E-05	3264.5	1615.3	1.10	2.70E-05	Up-regulated	multidrug resistance protein 4
LOC_Os01g50100	24hr	440.3	727.4	-0.70	7.80E-05	594.5	612.5	-0.10	5.00E-01	Un-changed	
Os.11816.1.S1_at	6hr	2380.5	752.3	1.60	2.00E-05	2776.1	920.6	1.60	2.00E-05	Up-regulated	NAD(P)H-dependent oxidoreductase
LOC_Os04g37490	24hr	1439.8	942	0.80	6.00E-05	2144.9	763.2	1.50	2.00E-05	Un-changed	
Os.12145.1.S1_at	6hr	594.6	314.5	1.00	2.00E-05	497.1	240.6	1.10	4.60E-05	Up-regulated	cytochrome P450 90D2
LOC_Os02g47470	24hr	465.3	154	1.80	2.00E-05	364.7	212.6	0.70	1.89E-04	Un-changed	
Os.12498.1.S1_at	6hr	247.4	77.7	2.20	8.80E-05	248	18	3.20	3.50E-05	Up-regulated	ORG3
LOC_Os01g72370	24hr	300.2	413.1	-0.40	2.48E-02	107.4	343.2	-3.40	2.00E-05	Un-changed	
Os.12629.1.S1_at	6hr	29286.1	9993.3	1.60	2.00E-05	24486.5	5696.8	2.00	2.00E-05	Up-regulated	expressed protein
LOC_Os01g45914	24hr	14389.9	28938.1	-0.90	2.00E-05	9919	33847.8	-1.80	2.00E-05	Un-changed	
Os.12629.1.S2_at	6hr	424.4	78.1	2.50	2.30E-05	211.9	141.6	1.40	3.00E-05	Up-regulated	expressed protein
LOC_Os01g45914	24hr	396.4	706.1	-0.80	1.30E-04	138.4	691.6	-2.40	2.00E-05	Un-changed	
Os.1327.1.S1_at	6hr	1543.1	707	1.10	2.00E-05	1492.6	587.9	1.20	2.00E-05	Up-regulated	glycine-rich cell wall structural protein 2
LOC_Os02g43540	24hr	917.2	745.2	0.50	6.00E-05	954.6	587.4	0.50	8.65E-04	Un-changed	precursor
Os.14121.1.S1_at	6hr	1002.1	475.4	1.20	2.00E-05	838.1	327.1	1.30	2.00E-05	Up-regulated	N-acylethanolamine amidohydrolase
LOC_Os11g06900	24hr	1097.5	458.3	1.30	2.00E-05	454.8	535.7	-0.40	1.81E-02	Un-changed	
Os.14616.1.S1_at	6hr	1652	402.1	1.90	6.00E-05	1308.4	185.1	2.80	2.00E-05	Up-regulated	chalcone synthase DII
LOC_Os07g34260	24hr	217	215.4	0.20	6.56E-02	227.6	267.3	-0.30	5.41E-03	Un-changed	
Os.16325.1.S1_at	6hr	1472.8	343.1	2.00	2.00E-05	960.7	355.8	1.40	2.00E-05	Up-regulated	potassium transporter 16
LOC_Os03g37830	24hr	650.1	392.8	0.80	2.00E-05	713.6	348.6	0.80	3.00E-05	Un-changed	
Os.17030.1.S1_at	6hr	1397.9	627.5	1.20	5.20E-05	1315.3	595.2	1.30	2.00E-05	Up-regulated	C2 domain containing protein
LOC_Os01g03820	24hr	632	297.6	0.60	2.00E-05	593.4	333.9	0.70	9.66E-04	Un-changed	
Os.1726.1.S1_at	6hr	1482.6	752.5	1.00	6.80E-05	1716.2	673.2	1.40	2.00E-05	Up-regulated	MYB2
LOC_Os01g18240	24hr	1158.5	804.2	0.40	1.14E-04	1348.6	1232.2	0.10	1.53E-01	Un-changed	
Os.17536.1.S1_at	6hr	722.7	271.5	1.60	2.00E-05	531.1	272.6	1.00	6.18E-04	Up-regulated	expressed protein
LOC_Os09g26670	24hr	630.2	1295.4	-1.10	2.00E-05	1087.1	1309.6	-0.20	6.56E-02	Un-changed	
Os.17887.1.S1_at	6hr	700.6	235.5	1.40	2.00E-05	773.6	316.5	1.20	6.80E-05	Up-regulated	calmodulin binding protein
LOC_Os12g36910	24hr	361.4	222.9	0.80	2.00E-05	521.2	285.7	1.20	2.00E-05	Un-changed	
Os.17900.1.S1_s_at	6hr	477.7	112.5	1.80	2.70E-05	302.9	87.7	1.80	2.00E-05	Up-regulated	gibberellin-regulated protein 2 precursor

Probe Set ID/Locus ID	Time	Quinclorac vs. Mock Repeat 1				Quinclorac vs. Mock Repeat 2				Assignment	Description
		Quinclorac	Mock	Log Ratio	P-value	Quinclorac	Mock	Log Ratio	P-value		
LOC_Os05g35690	24hr	32.4	11.9	1.40	5.00E-01	13.3	60.5	-1.30	2.23E-01	Un-changed	
Os.17916.1.S1_at	6hr	4818.6	848.1	2.50	2.00E-05	3985.1	627.6	2.90	2.00E-05	Up-regulated	expressed protein
LOC_Os05g12630	24hr	1605.8	1467.3	0.30	3.10E-02	3779.3	1987.3	0.90	2.00E-05	Un-changed	
Os.18559.1.S1_at	6hr	1637.6	470.2	1.90	2.00E-05	1144.6	259.4	1.70	2.30E-05	Up-regulated	beta-lactamase, class A
LOC_Os02g45520	24hr	952.9	841.1	0.60	2.30E-05	747.9	697.4	0.00	5.00E-01	Un-changed	
Os.18597.1.S1_at	6hr	1013.4	399.7	1.30	5.20E-05	939	377.8	1.60	2.00E-05	Up-regulated	sulfate transporter 2.1
LOC_Os03g09930	24hr	1599	825	0.80	2.00E-05	1748.5	1195.3	0.60	2.70E-05	Un-changed	
Os.18856.1.S1_at	6hr	3689.9	1295.5	1.60	2.00E-05	3734.3	915.6	2.10	2.00E-05	Up-regulated	bifunctional 3-phosphoadenosine 5-phosphosulfate
LOC_Os04g02050	24hr	3362.7	3059.1	0.20	2.75E-03	3184	2728.6	0.30	1.83E-03	Un-changed	synthetase 2
Os.19141.1.S1_at	6hr	1242.8	424.1	1.40	2.00E-05	979.7	441.9	1.20	2.70E-05	Up-regulated	expressed protein
LOC_Os01g47630	24hr	3652.7	3470.6	0.20	7.74E-04	2478.7	2708.2	0.00	5.00E-01	Un-changed	
Os.19519.1.S1_s_at	6hr	5882.3	1646.9	1.80	2.00E-05	6602.1	1874.6	1.80	2.00E-05	Up-regulated	OsAPRL1 - Oryza sativa adenosine 5'-phosphosulfate
LOC_Os07g32570	24hr	4543.5	2352	0.90	2.70E-05	9030.4	2842.7	1.60	2.00E-05	Un-changed	reductase-like
Os.2019.1.S1_at	6hr	1870.1	653.8	1.50	2.00E-05	1379.8	559.2	1.30	2.00E-05	Up-regulated	seven-transmembrane-domain protein 1
LOC_Os01g50460	24hr	5054.5	5904	-0.20	5.00E-01	4301	5579	-0.40	1.01E-04	Un-changed	
Os.2019.2.S1_x_at	6hr	1964	589.5	1.50	2.70E-05	1631.7	569.9	1.20	2.00E-05	Up-regulated	seven-transmembrane-domain protein 1
LOC_Os01g50460	24hr	6294.7	6893.4	-0.10	5.00E-01	5173.7	6726.4	-0.40	2.30E-05	Un-changed	
Os.21280.1.S1_at	6hr	920.5	457.5	1.50	3.00E-05	1204.1	568.3	1.30	2.30E-05	Up-regulated	O-methyltransferase ZRP4
LOC_Os12g25490	24hr	3813.4	2359	0.80	2.30E-05	2767.3	2776.4	0.00	5.00E-01	Un-changed	
Os.21839.1.S1_at	6hr	1031.6	669	1.10	2.14E-04	860.7	535.9	1.10	2.00E-05	Up-regulated	gibberellin receptor GID1L2
LOC_Os06g11130	24hr	1023.2	990.8	0.40	3.36E-03	1077.4	876.9	0.50	1.89E-04	Un-changed	
Os.21858.1.S1_at	6hr	380.8	102.2	1.90	6.80E-05	336.3	131.8	1.20	7.80E-05	Up-regulated	cDNA clone:J023105E08, full insert sequence.
	24hr	196.6	149.2	0.10	3.79E-01	70.2	177.8	-0.60	1.89E-04	Un-changed	
Os.21858.2.S1_x_at	6hr	1083.7	374.7	1.40	2.00E-05	781.8	296.1	1.30	2.30E-05	Up-regulated	Oryza sativa U43a snoRNA.
	24hr	621.4	475.7	0.40	4.92E-04	241.8	378.8	-0.60	5.20E-05	Un-changed	
Os.22580.1.S1_s_at	6hr	2231	692	1.80	2.00E-05	2213.2	991.9	1.20	2.00E-05	Up-regulated	bundle sheath cell specific protein 1
LOC_Os01g73250	24hr	533.8	549.2	-0.30	2.74E-01	370.8	510.9	-0.30	7.89E-02	Un-changed	
Os.22832.1.S1_at	6hr	1628.9	521.4	1.70	2.30E-05	1498	339.4	2.20	7.80E-05	Up-regulated	expressed protein
LOC_Os09g24620	24hr	436.7	354.5	0.40	4.48E-03	565.1	392.1	0.50	4.93E-03	Un-changed	
Os.23113.1.S1_at	6hr	9682.7	3132.7	1.80	2.00E-05	9100	2711.3	1.80	2.00E-05	Up-regulated	expressed protein
LOC_Os04g34610	24hr	2191.9	1789.1	0.20	8.89E-02	1801.1	1915.9	-0.20	4.81E-01	Un-changed	
Os.24363.1.A1_at	6hr	1030.8	183.3	2.30	3.00E-05	876.1	118	2.80	4.60E-05	Up-regulated	CSLA11 - cellulose synthase-like family A
LOC_Os08g33740	24hr	392.9	312.2	0.50	1.81E-02	868.7	428.5	0.90	6.92E-04	Un-changed	
Os.26437.1.A1_at	6hr	226.8	138.8	1.00	6.18E-04	318.7	198.1	1.00	1.67E-04	Up-regulated	light-inducible protein CPRF-2
LOC_Os12g40920	24hr	35.3	41.6	0.10	5.00E-01	36.5	38.4	0.40	5.00E-01	Un-changed	
Os.26437.1.A1_s_at	6hr	473.7	150.6	1.40	1.67E-04	598.9	269.5	1.20	3.36E-03	Up-regulated	light-inducible protein CPRF-2
LOC_Os12g40920	24hr	20.7	17.3	-0.20	4.94E-01	61.7	10.4	3.00	5.00E-01	Un-changed	
Os.26512.1.S1_at	6hr	271.2	117	1.10	2.00E-05	403.6	90.2	2.10	3.00E-05	Up-regulated	OsIAA14 - Auxin-responsive Aux/IAA gene family
LOC_Os03g58350	24hr	432.8	206.8	1.00	2.00E-05	402.6	284.7	0.20	5.06E-02	Un-changed	member
Os.2677.1.S1_at	6hr	5638.7	1280.4	2.10	2.00E-05	4416.1	1220.7	1.70	2.00E-05	Up-regulated	galactinol synthase 3
LOC_Os07g48830	24hr	1829.9	1224.3	0.70	2.00E-05	1693	1394.5	0.40	3.07E-04	Un-changed	
Os.27494.1.S1_at	6hr	2501.7	1202.2	1.10	2.00E-05	2374.7	740.2	1.50	2.00E-05	Up-regulated	expressed protein
LOC_Os07g41200	24hr	2831.6	1870.1	0.70	2.00E-05	2958.5	1794.9	0.80	2.00E-05	Un-changed	
Os.27606.1.S1_at	6hr	2166.2	1070.9	1.00	2.00E-05	1914.4	899.1	1.20	2.00E-05	Up-regulated	aspartokinase
LOC_Os03g63330	24hr	1336.9	733.4	0.90	2.00E-05	1302.2	590.1	1.20	2.00E-05	Un-changed	
Os.27906.1.S1_a_at	6hr	858.2	374.9	1.70	3.00E-05	568	289.9	1.50	3.50E-05	Up-regulated	protein phosphatase 2C
LOC_Os01g62760	24hr	483.9	503.6	0.50	2.49E-03	455.9	441	1.20	1.30E-04	Un-changed	
Os.28129.1.S1_at	6hr	885.9	299.7	1.50	2.00E-05	725.9	244.3	1.50	4.00E-05	Up-regulated	cytokinin-O-glucosyltransferase 1
LOC_Os05g42040	24hr	591.3	333.2	1.10	3.50E-05	448.8	285.7	0.60	7.12E-03	Un-changed	
Os.28290.1.S1_at	6hr	9338.3	3280.3	1.50	2.00E-05	7156.9	2803.6	1.30	2.00E-05	Up-regulated	carotenoid cleavage dioxygenase 1
LOC_Os02g47510	24hr	641.8	995.7	-0.80	3.89E-04	625.7	777.5	-0.40	1.34E-03	Un-changed	
Os.30664.1.S1_at	6hr	877.9	378	1.30	5.20E-05	673.6	385.8	1.20	3.07E-04	Up-regulated	cytochrome P450 71A1
LOC_Os01g12740	24hr	564.2	728.8	-0.10	4.81E-01	744.1	701	0.30	1.96E-02	Un-changed	

Probe Set ID/Locus ID	Time	Quinclorac vs. Mock Repeat 1				Quinclorac vs. Mock Repeat 2				Assignment	Description
		Quinclorac	Mock	Log Ratio	P-value	Quinclorac	Mock	Log Ratio	P-value		
Os.30866.1.S1_at	6hr	1068	251.2	2.20	1.47E-04	1042.1	296.5	1.60	3.00E-05	Up-regulated	expressed protein
LOC_Os07g02460	24hr	205.3	189.4	0.30	5.00E-01	516.6	181.6	1.30	2.41E-04	Un-changed	
Os.33939.1.A1_at	6hr	1797.5	699.1	1.40	2.00E-05	1239.5	479	1.20	4.38E-04	Up-regulated	expressed protein
LOC_Os01g04590	24hr	874.2	684.2	0.50	1.01E-02	1047.8	707.2	0.50	7.12E-03	Un-changed	
Os.34139.1.S1_at	6hr	911.7	541.3	1.00	3.50E-05	896.5	461.7	1.10	2.00E-05	Up-regulated	expressed protein
LOC_Os08g04630	24hr	338.9	276	0.50	2.49E-03	466.5	326.9	0.60	4.48E-03	Un-changed	
Os.34462.1.S1_at	6hr	368	179.9	1.20	6.80E-05	339.2	100.3	1.40	2.30E-05	Up-regulated	ras-related protein Rab11C
LOC_Os01g62950	24hr	333.1	230.3	0.30	3.46E-04	195	214.7	-0.10	5.00E-01	Un-changed	
Os.34873.1.S1_at	6hr	297.8	81.2	1.70	4.00E-05	248.4	129	1.00	3.46E-04	Up-regulated	expressed protein
LOC_Os07g46510	24hr	126.8	136	-0.40	5.00E-01	200	149.3	0.60	3.33E-02	Un-changed	
Os.35573.1.S1_at	6hr	433.5	325.4	1.10	4.00E-05	444.7	290.7	1.10	8.80E-05	Up-regulated	sigma factor sigB regulation protein rsbQ
LOC_Os01g41240	24hr	177.6	302.1	-0.60	5.40E-02	408.6	296.5	0.60	1.10E-02	Un-changed	
Os.38414.1.S1_at	6hr	385.1	91.5	1.90	2.30E-05	327.6	85.1	1.20	6.92E-04	Up-regulated	granule-bound starch synthase 1, chloroplast precursor
LOC_Os06g04200	24hr	255.7	422.9	-0.70	2.00E-05	219.7	273.4	-0.50	9.99E-02	Un-changed	
Os.39505.1.S1_at	6hr	2291.3	716.1	1.60	2.00E-05	2217.2	813.2	1.30	2.00E-05	Up-regulated	ABA-induced protein
LOC_Os02g50140	24hr	3048.7	2028.9	0.60	3.00E-05	2873.5	2228.2	0.30	2.00E-05	Un-changed	
Os.39636.1.A1_x_at	6hr	1198.5	1053.8	1.00	5.52E-04	2745.9	1671.2	1.10	6.00E-05	Up-regulated	indole-3-acetate beta-glucosyltransferase
LOC_Os04g12720	24hr	39.1	216.5	-2.80	1.01E-04	75.5	76.7	-0.50	6.56E-02	Un-changed	
Os.40018.1.S1_at	6hr	955.8	354.4	1.20	2.00E-05	928.9	564.3	1.00	1.08E-03	Up-regulated	heat shock factor
LOC_Os05g45410	24hr	148.8	276.5	-0.40	2.33E-01	312.4	219.7	0.50	2.88E-02	Un-changed	
Os.40598.1.S1_x_at	6hr	832.5	414.5	1.10	2.00E-05	1142.6	533	1.00	5.52E-04	Up-regulated	LEC14B
LOC_Os01g28680	24hr	1352.8	880.6	0.60	4.38E-04	1414.5	727.5	0.80	4.38E-04	Un-changed	
Os.4179.1.S1_at	6hr	7013.2	2711.8	1.40	2.00E-05	5217.1	2074.3	1.30	2.00E-05	Up-regulated	1,4-alpha-glucan branching enzyme IIB, chloroplast precursor
LOC_Os04g33460	24hr	1507.4	1485.4	0.10	3.91E-01	1237.5	1590.2	-0.30	7.12E-03	Un-changed	
Os.43491.1.S1_x_at	6hr	3517.7	1220.9	1.20	2.00E-05	2744.9	1140.3	1.30	2.00E-05	Up-regulated	esterase precursor
LOC_Os01g11620	24hr	4399.3	1870.2	1.30	2.00E-05	2677.3	1700.5	0.70	2.00E-05	Un-changed	
Os.45887.1.S1_at	6hr	1180.7	458.3	1.10	5.20E-05	1413.1	510.3	1.20	2.41E-04	Up-regulated	cytochrome P450 71A4
LOC_Os01g12750	24hr	267.1	412.4	-0.50	4.92E-04	495.1	395.9	0.40	6.18E-04	Un-changed	
Os.45928.1.S1_at	6hr	2588.3	752.5	1.90	2.00E-05	2371	709.2	2.10	2.00E-05	Up-regulated	expressed protein
LOC_Os01g32460	24hr	3280.8	1657.6	1.10	2.30E-05	1946.7	1622.4	0.30	1.14E-04	Un-changed	
Os.46081.1.S1_at	6hr	957.9	313.7	1.70	2.00E-05	465	183.5	1.40	3.00E-05	Up-regulated	expressed protein
LOC_Os10g13850	24hr	3975.3	4179	0.00	5.00E-01	2929.5	2881.3	0.20	2.85E-01	Un-changed	
Os.4627.1.S1_x_at	6hr	6783.5	3071	1.00	2.00E-05	5946	2736.5	1.10	2.00E-05	Up-regulated	CBL-interacting serine/threonine-protein kinase 15
LOC_Os07g48100	24hr	5234.5	3660.4	0.40	2.70E-05	5421.2	3470.4	0.60	2.00E-05	Un-changed	
Os.46633.1.A1_at	6hr	244.6	108.5	1.50	2.00E-05	298.5	127.6	1.10	2.00E-05	Up-regulated	beta-carotene hydroxylase
LOC_Os10g38940	24hr	2057.6	1252.1	0.80	2.00E-05	1372.1	1242.2	-0.10	5.00E-01	Un-changed	
Os.46635.1.S1_x_at	6hr	682.7	242.7	1.40	6.18E-04	737.9	242.9	1.60	2.70E-05	Up-regulated	glutathione S-transferase GSTU6
LOC_Os10g38350	24hr	303.1	272.1	0.30	2.12E-02	286.9	184.1	1.60	1.54E-02	Un-changed	
Os.467.1.S1_a_at	6hr	2449.9	1179.1	1.10	2.30E-05	2282.9	1396.6	1.10	2.00E-05	Up-regulated	PDR5-like ABC transporter
LOC_Os01g42380	24hr	511.9	458.6	-0.10	5.00E-01	651.2	467	0.40	5.41E-03	Un-changed	
Os.46725.1.S1_at	6hr	289.1	66.9	2.70	1.65E-03	308.8	134.8	1.90	3.89E-04	Up-regulated	expressed protein
LOC_Os10g05130	24hr	136.9	284.9	-1.30	4.00E-05	342.7	192	0.50	1.10E-02	Un-changed	
Os.46884.1.S1_at	6hr	844.6	447.7	1.00	1.49E-03	731.2	387.5	1.10	4.00E-05	Up-regulated	expressed protein
LOC_Os10g39000	24hr	731.8	656.2	0.60	7.12E-03	868.7	631.7	0.90	3.07E-04	Un-changed	
Os.4700.1.S1_at	6hr	1263.1	508.2	1.10	2.00E-05	1159.9	406	1.50	2.00E-05	Up-regulated	sodium/hydrogen exchanger 2
LOC_Os07g47100	24hr	1818.7	1021.2	0.90	2.00E-05	2646	1044.4	1.40	2.00E-05	Un-changed	
Os.4741.2.S1_x_at	6hr	3575.5	1637.2	1.30	2.00E-05	4033.2	1632.3	1.40	2.00E-05	Up-regulated	catalytic/ hydrolase
LOC_Os10g41930	24hr	3945.6	3332	0.50	2.00E-05	5380.8	2767.2	1.00	2.00E-05	Un-changed	
Os.48625.1.A1_s_at	6hr	4075.8	2772.1	1.30	2.00E-05	4713.9	1848.2	1.20	2.00E-05	Up-regulated	CCT motif family protein
LOC_Os05g51690	24hr	1349.9	868.6	0.60	4.60E-05	1415.7	875.4	0.80	2.00E-05	Un-changed	
Os.48761.1.S1_at	6hr	1117.8	355.7	1.50	2.00E-05	665.2	248.1	1.50	2.30E-05	Up-regulated	bile acid sodium symporter
LOC_Os02g27490	24hr	108.6	102.8	0.20	5.00E-01	153.5	158.9	-0.20	5.00E-01	Un-changed	
Os.48846.1.S1_at	6hr	284.7	61.7	1.80	1.30E-04	225.5	73.3	1.50	3.00E-05	Up-regulated	GAST1 protein precursor

Probe Set ID/Locus ID	Time	Quinclorac vs. Mock Repeat 1				Quinclorac vs. Mock Repeat 2				Assignment	Description
		Quinclorac	Mock	Log Ratio	P-value	Quinclorac	Mock	Log Ratio	P-value		
LOC_Os09g24840	24hr	172.5	142.4	0.40	4.41E-02	105.1	137.7	-0.30	6.15E-02	Un-changed	
Os.49182.1.S1_at	6hr	587.4	394.6	1.10	2.73E-04	828.4	384.5	1.30	1.47E-04	Up-regulated	expressed protein
LOC_Os02g36590	24hr	609	448.1	0.50	1.49E-03	813.4	541.4	0.60	8.65E-04	Un-changed	
Os.49198.1.S1_at	6hr	1002.4	289.9	1.40	3.00E-05	688	294.7	1.10	5.20E-05	Up-regulated	NHL25
LOC_Os04g58090	24hr	126.2	135.4	0.00	5.00E-01	260.7	130.6	1.20	2.03E-03	Un-changed	
Os.49527.1.S1_at	6hr	920	404.6	1.20	2.73E-04	660.2	296.7	1.00	3.46E-04	Up-regulated	acyl-activating enzyme 11
LOC_Os03g03790	24hr	609.2	556.9	0.00	5.00E-01	513.5	991.3	-0.70	3.89E-04	Un-changed	
Os.50175.2.S1_at	6hr	396.6	114.6	1.70	2.00E-05	342.3	153.5	1.10	2.00E-05	Up-regulated	cation transporter HKT4
LOC_Os04g51820	24hr	237.6	247.5	0.00	5.00E-01	493.3	266.9	0.70	3.50E-05	Un-changed	
Os.50438.1.S1_at	6hr	5901.6	2330	1.30	2.00E-05	4583.3	2526.1	1.00	2.00E-05	Up-regulated	expressed protein
LOC_Os02g36530	24hr	824.8	1566.2	-1.00	3.50E-05	1511.2	1377.7	0.40	1.83E-03	Un-changed	
Os.50455.1.S1_at	6hr	140.5	30.6	2.20	6.00E-05	87.6	21.7	2.70	2.73E-04	Up-regulated	phospholipase D alpha 2
LOC_Os06g40170	24hr	54.7	38.5	0.50	5.40E-02	16.2	27.2	-1.00	4.92E-03	Un-changed	
Os.51226.1.S1_at	6hr	748.1	265.3	1.50	2.00E-05	615.6	151.8	2.00	2.00E-05	Up-regulated	uncharacterized UPF0114 domain containing protein
LOC_Os03g52910	24hr	1004.9	259.4	2.30	2.00E-05	225.6	269	0.00	5.00E-01	Un-changed	
Os.51227.1.S1_s_at	6hr	149.6	46.5	1.40	1.01E-04	147.3	46.4	2.70	1.30E-04	Up-regulated	terpene synthase 8
LOC_Os04g27790	24hr	1.9	9.5	-2.40	5.00E-01	4.4	0.9	1.30	8.51E-03	Un-changed	
Os.51227.1.S1_x_at	6hr	545.9	171.3	1.70	2.00E-05	475.5	96.1	1.90	2.00E-05	Up-regulated	terpene synthase 8
LOC_Os04g27790	24hr	19.9	48.6	-0.90	3.67E-01	18.4	77.3	-2.20	9.99E-02	Un-changed	
Os.51337.1.S1_at	6hr	363.4	232.2	1.10	2.00E-05	395.1	71.9	2.20	2.00E-05	Up-regulated	indole-3-acetate beta-glucosyltransferase
LOC_Os06g39330	24hr	869	582.7	0.70	2.30E-05	1280.1	382.4	1.70	2.00E-05	Un-changed	
Os.51460.1.S1_at	6hr	625.3	312.1	1.00	2.00E-05	650.8	216.4	1.70	2.30E-05	Up-regulated	pectinesterase inhibitor domain containing protein
LOC_Os02g33380	24hr	905.6	736.8	0.30	1.54E-02	2740.8	997.1	1.40	2.00E-05	Un-changed	
Os.52345.1.S1_at	6hr	587.9	335.6	1.00	2.00E-05	602.3	343.4	1.10	2.00E-05	Up-regulated	ATP binding protein
LOC_Os01g60280	24hr	163.7	245.5	-0.50	5.06E-02	215.1	131.1	0.30	1.95E-01	Un-changed	
Os.52767.1.A1_at	6hr	1985.2	906.6	1.50	2.00E-05	2158.7	749.4	1.30	2.30E-05	Up-regulated	chaperone protein dnaJ
LOC_Os08g35160	24hr	267.9	587.7	-0.90	2.30E-05	436.6	627.5	-0.60	1.47E-04	Un-changed	
Os.5318.1.S1_a_at	6hr	1790.7	688.2	1.60	2.00E-05	1236.3	518.9	1.60	2.00E-05	Up-regulated	TPR domain protein
LOC_Os10g42610	24hr	749.5	448.2	0.90	6.00E-05	307.1	336.4	-0.40	3.33E-02	Un-changed	
Os.53884.1.S1_at	6hr	1232	737.9	1.00	2.00E-05	1161.7	734.4	1.00	2.00E-05	Up-regulated	trans-cinnamate 4-monooxygenase
LOC_Os05g25640	24hr	1504.7	1222.7	0.60	2.70E-05	1124.3	1153.9	0.40	1.67E-02	Un-changed	
Os.54130.1.S1_at	6hr	270.1	63.6	2.20	2.00E-05	225.1	73.9	1.50	2.00E-05	Up-regulated	expressed protein
LOC_Os04g40050	24hr	64.8	62.4	-0.20	5.00E-01	8	60.7	-3.50	8.51E-03	Un-changed	
Os.54440.1.S1_at	6hr	737.9	376.7	1.30	2.73E-04	751.2	358.3	1.20	3.04E-03	Up-regulated	expressed protein
LOC_Os04g53180	24hr	807.6	725.9	-0.20	1.77E-01	1032.9	1165.7	-0.10	2.96E-01	Un-changed	
Os.5492.1.S1_at	6hr	4055.3	1892.7	1.20	2.00E-05	3254.3	1537.5	1.10	2.00E-05	Up-regulated	peptidyl-prolyl cis-trans isomerase CYP19-3
LOC_Os09g39780	24hr	1367	1213.8	0.30	2.29E-02	965.8	1113.8	-0.10	5.00E-01	Un-changed	
Os.54940.1.S1_at	6hr	362.8	99	1.70	6.80E-05	160.3	42.4	1.60	4.41E-02	Up-regulated	gibberellin 2-beta-dioxygenase 7
LOC_Os04g44150	24hr	92.1	99.2	0.00	1.38E-01	48.7	96.8	-1.00	1.61E-01	Un-changed	
Os.5495.1.S1_at	6hr	4828.7	2244.1	1.10	2.00E-05	3130.8	1618.2	1.00	2.00E-05	Up-regulated	expressed protein
LOC_Os04g37710	24hr	9479	6624.2	0.50	2.00E-05	8049.9	4823	0.70	2.00E-05	Un-changed	
Os.5560.1.S1_at	6hr	6070	2427.5	1.30	2.00E-05	6305.4	1785.8	1.70	2.00E-05	Up-regulated	aldose reductase
LOC_Os01g62870	24hr	6344.3	3834.6	0.80	2.00E-05	5611	3196.7	0.80	2.00E-05	Un-changed	
Os.57519.1.S1_x_at	6hr	11681.7	3864.9	1.40	2.00E-05	7267.2	2058.6	1.70	2.00E-05	Up-regulated	early light-induced protein, chloroplast precursor
LOC_Os01g14410	24hr	19473.2	24609.4	-0.30	2.41E-04	27300.4	27110.1	-0.20	3.67E-01	Un-changed	
Os.57569.2.A1_s_at	6hr	2368.3	512.5	2.20	2.00E-05	1841.6	198	3.00	2.00E-05	Up-regulated	terpene synthase 6
LOC_Os08g04500	24hr	10805	3588.5	1.40	2.00E-05	5683	4684	0.30	3.50E-05	Un-changed	
Os.7029.1.S1_at	6hr	4291.8	1337.6	1.80	2.00E-05	2213.8	889.3	1.50	2.00E-05	Up-regulated	expressed protein
LOC_Os05g24650	24hr	921.7	1395.8	-0.60	6.80E-05	1194	959	0.40	1.49E-03	Un-changed	
Os.7075.1.S1_at	6hr	6542.8	3007.6	1.20	2.00E-05	6377.3	2433.7	1.40	2.00E-05	Up-regulated	phospho-2-dehydro-3-deoxyheptonate aldolase 2, chloroplast precursor
LOC_Os03g27230	24hr	7670.7	4094.5	0.80	2.00E-05	5155.4	4182.7	0.30	2.00E-05	Un-changed	
Os.7304.1.S1_at	6hr	1006.2	337.1	1.50	2.00E-05	812.1	282	1.50	2.00E-05	Up-regulated	protein kinase
LOC_Os01g10450	24hr	661.7	396.1	0.30	1.08E-03	570.8	376.4	0.50	4.92E-04	Un-changed	

Probe Set ID/Locus ID	Time	Quinclorac vs. Mock Repeat 1				Quinclorac vs. Mock Repeat 2				Assignment	Description
		Quinclorac	Mock	Log Ratio	P-value	Quinclorac	Mock	Log Ratio	P-value		
Os.7496.1.S1_a_at	6hr	5247.2	1676.9	1.60	2.00E-05	3486.2	1006.4	1.80	3.50E-05	Up-regulated	mannitol dehydrogenase
LOC_Os10g29470	24hr	9937.6	4925.2	0.80	2.30E-05	8372.7	5867.8	0.40	7.80E-05	Un-changed	
Os.7507.1.S1_at	6hr	336.5	112.4	1.50	2.00E-05	381.5	109	1.50	4.60E-05	Up-regulated	O-methyltransferase ZRP4
LOC_Os10g02880	24hr	99.5	209.3	-1.30	2.00E-05	216.3	68.7	1.40	1.89E-04	Un-changed	
Os.7831.1.S1_at	6hr	1145.3	322.8	1.30	2.30E-05	821.6	313.9	1.50	8.80E-05	Up-regulated	expressed protein
LOC_Os06g11980	24hr	414.3	398.2	0.20	3.84E-02	406.6	404.6	0.10	5.77E-02	Un-changed	
Os.7985.1.S1_at	6hr	5869	2541	1.20	2.00E-05	5685.9	2262.4	1.30	2.00E-05	Up-regulated	omega-3 fatty acid desaturase, chloroplast precursor
LOC_Os03g18070	24hr	9423.8	13646.7	-0.50	2.00E-05	10655.5	16173.3	-0.70	2.00E-05	Un-changed	
Os.8031.1.S1_at	6hr	429.6	205.4	1.20	7.80E-05	743	325	1.00	2.00E-05	Up-regulated	ethylene-responsive factor-like protein 1
LOC_Os01g21120	24hr	108.1	55.7	0.70	5.41E-03	157.3	92.7	0.90	1.10E-02	Un-changed	
Os.8045.1.S1_at	6hr	21683.4	4420.5	2.20	2.00E-05	20432.5	4991.8	1.90	2.00E-05	Up-regulated	indole-3-acetate beta-glucosyltransferase
LOC_Os01g08440	24hr	16483.9	16038.3	0.70	4.60E-05	26133.5	17222	0.90	2.00E-05	Un-changed	
Os.8112.1.S1_at	6hr	2611.5	1193.1	1.20	2.00E-05	2564.7	1122.1	1.20	2.00E-05	Up-regulated	aldo-keto reductase/ oxidoreductase
LOC_Os07g04990	24hr	746.7	898.2	-0.30	8.51E-03	3153.2	1238.1	1.50	2.00E-05	Un-changed	
Os.8374.1.S1_at	6hr	346.2	166.4	1.10	3.89E-04	280.8	149.1	1.60	4.92E-04	Up-regulated	auxin response factor 16
LOC_Os04g43910	24hr	324.2	219.7	0.40	4.48E-03	237	217	0.40	7.89E-02	Un-changed	
Os.8504.1.S1_at	6hr	6766.3	3007.8	1.10	2.70E-05	5953.4	2470.3	1.10	3.00E-05	Up-regulated	nodulin-like protein
LOC_Os04g42420	24hr	7651.5	5034.2	0.60	2.70E-05	5954.6	4628	0.50	8.80E-05	Un-changed	
Os.8622.1.S1_at	6hr	2892.3	1810.2	1.00	2.00E-05	3292.3	1974	1.00	1.30E-04	Up-regulated	OsIAA24 - Auxin-responsive Aux/IAA gene family member
LOC_Os07g08460	24hr	3476.5	2295.3	0.40	1.30E-04	4382.5	2831	0.80	3.00E-05	Un-changed	
Os.8778.1.S1_a_at	6hr	1445.8	699.2	1.10	2.00E-05	1789.6	505.7	1.60	2.00E-05	Up-regulated	12-oxophytodienoate reductase 2
LOC_Os06g11290	24hr	885.9	363.3	0.90	3.50E-05	2072.8	444.8	2.30	2.00E-05	Un-changed	
Os.9022.1.S1_at	6hr	599.6	211.8	1.90	3.00E-05	581.5	251.5	1.00	1.83E-03	Up-regulated	protein phosphatase 2C
LOC_Os09g15670	24hr	166.9	116.6	0.50	4.48E-03	358.9	152.4	1.60	5.20E-05	Un-changed	
Os.9103.1.S1_at	6hr	2746.7	697.7	2.00	2.00E-05	2008.8	627.6	1.80	1.89E-04	Up-regulated	heavy metal-associated domain containing protein
LOC_Os09g09930	24hr	862.1	703.5	0.40	4.60E-05	1674.2	688.3	1.30	2.00E-05	Un-changed	
Os.9116.1.S1_at	6hr	7065.1	3093.6	1.00	2.00E-05	7870.6	3389.7	1.10	2.00E-05	Up-regulated	LEC14B
LOC_Os01g28680	24hr	7978.8	5195.4	0.80	2.00E-05	8887.9	5093	0.80	2.00E-05	Un-changed	
Os.9191.1.S1_s_at	6hr	13790.3	5162.6	1.40	2.00E-05	9389.7	3085.1	1.60	2.00E-05	Up-regulated	early light-induced protein, chloroplast precursor
LOC_Os01g14410	24hr	24134.3	31491.9	-0.30	3.50E-05	30388.2	34823.8	-0.20	2.29E-02	Un-changed	
Os.9212.1.S1_at	6hr	2020.5	474.7	2.10	2.00E-05	1907.7	398.9	2.10	2.00E-05	Up-regulated	granule-bound starch synthase 1b, chloroplast precursor
LOC_Os07g22930	24hr	4599	2479.6	0.80	2.00E-05	3582.4	3199.5	0.10	5.00E-01	Un-changed	
Os.9216.1.S1_at	6hr	946.2	396	1.40	2.00E-05	619.3	331.5	1.20	2.00E-05	Up-regulated	amelogenin precursor like protein
LOC_Os03g26210	24hr	1035.7	1965.3	-0.70	2.00E-05	409.3	1986.2	-2.30	2.00E-05	Un-changed	
Os.9494.1.S1_s_at	6hr	244.7	92.6	1.50	1.14E-04	327.1	115.5	1.40	4.60E-05	Up-regulated	short-chain dehydrogenase/reductase SDR
LOC_Os04g45000	24hr	358.2	228.5	1.20	2.30E-05	269.3	200.8	0.40	1.95E-01	Un-changed	
Os.9713.1.S1_at	6hr	953	372.4	1.30	2.00E-05	1163	441.4	1.40	2.00E-05	Up-regulated	CCT motif family protein
LOC_Os05g51690	24hr	281.8	153.2	1.00	2.00E-05	330.7	144.5	0.90	4.60E-05	Un-changed	
Os.9749.1.S1_at	6hr	8396	4409.8	1.00	2.00E-05	8428.9	3872.1	1.20	2.00E-05	Up-regulated	monodehydroascorbate reductase
LOC_Os08g44340	24hr	5416.5	3253.2	0.80	2.00E-05	6745.3	3031.6	1.30	2.00E-05	Un-changed	
Os.9875.1.S1_at	6hr	1343.3	628.1	1.20	2.00E-05	1091.9	354.5	1.70	2.00E-05	Up-regulated	cp protein
LOC_Os03g11210	24hr	750.1	776.5	0.10	5.00E-01	678	777.8	-0.40	1.01E-02	Un-changed	
OsAffx.11838.1.S1_x_at	6hr	2780.8	445.2	1.70	6.00E-05	2592.4	1050.4	1.30	4.00E-05	Up-regulated	bundle sheath cell specific protein 1
LOC_Os01g73250	24hr	568.7	943	-0.20	5.00E-01	219.6	305.7	-0.10	5.00E-01	Un-changed	
OsAffx.12508.1.S1_at	6hr	1657.1	508.2	1.70	2.00E-05	1141.5	361.1	1.60	2.00E-05	Up-regulated	beta-lactamase, class A
LOC_Os02g45520	24hr	1305.1	818.9	0.70	2.00E-05	654.1	573.7	0.20	3.42E-01	Un-changed	
OsAffx.13348.1.S1_s_at	6hr	411.5	86.1	1.50	8.80E-05	360.2	107.3	1.30	2.70E-05	Up-regulated	cytochrome P450 93A3
LOC_Os03g44740	24hr	272.6	284.1	-0.30	4.41E-02	371.2	351.2	0.10	5.00E-01	Un-changed	
OsAffx.14582.1.S1_at	6hr	204.1	84.8	1.40	2.00E-05	230.7	103.7	1.20	3.70E-03	Up-regulated	60S ribosomal protein L10-3
LOC_Os05g07700	24hr	196	79.3	0.80	3.36E-03	272.3	64.2	1.90	5.52E-04	Un-changed	
OsAffx.16888.1.S1_at	6hr	1590	459	1.70	2.00E-05	1328	618	1.00	2.00E-05	Up-regulated	11674.m00736
LOC_Os08g07990	24hr	145.8	244.4	-0.30	5.77E-02	160	134.1	0.00	2.74E-01	Un-changed	
OsAffx.17942.1.S1_at	6hr	609.6	341	1.10	2.00E-05	696.5	400.1	1.10	2.00E-05	Up-regulated	phosphate carrier protein, mitochondrial precursor

Probe Set ID/Locus ID	Time	Quinclorac vs. Mock Repeat 1				Quinclorac vs. Mock Repeat 2				Assignment	Description
		Quinclorac	Mock	Log Ratio	P-value	Quinclorac	Mock	Log Ratio	P-value		
LOC_Os09g28160	24hr	300.8	226.9	0.70	5.52E-04	424.5	253.6	1.00	6.00E-05	Un-changed	
OsAffx.20062.1.S1_at	6hr	512.6	175.1	1.50	2.00E-05	625	198.1	1.50	2.00E-05	Up-regulated	light-inducible protein CPRF-2
LOC_Os12g40920	24hr	1.6	30.9	-3.40	1.96E-02	23.2	5.2	2.00	4.16E-01	Un-changed	
OsAffx.21790.1.S1_at	6hr	980.1	296.6	1.70	2.00E-05	703.4	216.6	1.50	2.00E-05	Up-regulated	4-coumarate--CoA ligase
LOC_Os01g67540	24hr	281	336.1	-0.10	3.79E-01	525.1	411	0.40	2.41E-04	Un-changed	
OsAffx.23032.1.S1_at	6hr	677.1	280.9	1.30	1.67E-04	411	127.3	1.20	1.89E-04	Up-regulated	expressed protein
LOC_Os01g01670	24hr	222.2	122.1	0.80	1.06E-01	28.5	34.1	-0.30	2.74E-01	Un-changed	
OsAffx.24138.1.S1_s_at	6hr	863.4	177.1	2.20	3.50E-05	649.9	93.1	2.90	8.80E-05	Up-regulated	lipopolysaccharide-responsive and beige-like
LOC_Os02g06720	24hr	49.2	41.8	-0.20	1.61E-01	166.6	72.2	1.10	2.41E-04	Un-changed	anchor protein
OsAffx.24280.2.S1_at	6hr	6144.8	950.1	2.60	2.00E-05	2603.8	318.6	2.50	2.00E-05	Up-regulated	early light-induced protein, chloroplast precursor
LOC_Os07g08150	24hr	16873.6	18848.4	-0.10	5.00E-01	15443.7	17906.1	-0.10	5.00E-01	Un-changed	
OsAffx.25073.1.S1_x_at	6hr	1489.4	276.2	2.80	2.00E-05	827.8	146.4	1.60	4.93E-03	Up-regulated	expressed protein
LOC_Os03g12700	24hr	2457.9	2423.4	0.00	5.00E-01	2393.4	2272.8	0.20	2.53E-01	Un-changed	
OsAffx.25760.1.S1_x_at	6hr	402.6	263.1	1.00	8.80E-05	528.1	269.1	1.10	2.00E-05	Up-regulated	ATPase 2
LOC_Os03g58800	24hr	318.7	182.2	0.20	5.77E-02	343.5	241.9	0.70	3.10E-02	Un-changed	
OsAffx.26254.1.S1_at	6hr	1531.3	570.3	1.30	2.00E-05	1151.7	646.2	1.00	2.00E-05	Up-regulated	transparent testa 12 protein
LOC_Os04g30490	24hr	429.3	305.1	0.20	5.00E-01	219.6	194.6	0.20	5.00E-01	Un-changed	
OsAffx.26671.1.S1_x_at	6hr	384	40.5	4.00	2.00E-05	332.4	13.3	3.70	2.00E-05	Up-regulated	expressed protein
LOC_Os04g53612	24hr	3.4	5	-0.70	3.67E-01	6.8	4.7	-0.30	1.25E-01	Un-changed	
OsAffx.28714.1.S1_x_at	6hr	1177.4	202.8	2.20	2.30E-05	703.8	200.4	2.00	6.18E-04	Up-regulated	OsAPRL1 - Oryza sativa adenosine 5'-phosphosulfate
LOC_Os07g32570	24hr	362	372.4	0.20	3.31E-01	965.8	299.9	1.60	2.00E-05	Un-changed	reductase-like
OsAffx.29571.1.S1_at	6hr	479.6	188.3	1.00	4.92E-04	742.2	225.9	1.40	3.46E-04	Up-regulated	transparent testa 12 protein
LOC_Os08g37432	24hr	567.8	365	0.80	1.14E-04	858.5	282.4	1.60	2.00E-05	Un-changed	
OsAffx.4296.1.S1_at	6hr	1286.5	498.2	1.50	5.20E-05	962.1	326	1.50	2.30E-05	Up-regulated	OsGrx_C15 - glutaredoxin subgroup III
LOC_Os05g10930	24hr	942.2	641.2	0.60	4.92E-04	668.6	437.8	0.60	3.46E-04	Un-changed	
OsAffx.6015.1.S1_at	6hr	1258.1	234.5	2.50	2.00E-05	731.2	124	2.50	2.00E-05	Up-regulated	CSLA11 - cellulose synthase-like family A
LOC_Os08g33740	24hr	407.7	265.4	0.40	5.20E-05	495.9	369.3	0.50	2.30E-05	Un-changed	
Os.12430.1.S1_at	6hr	13832.3	2806.9	2.40	2.00E-05	7693	1049.5	3.00	2.00E-05	Up-regulated	contains ESTs D49239(S16379),AU101865(S16379)
	24hr	2303.4	6113.7	-1.40	2.00E-05	1104.1	10594	-3.00	2.00E-05	Down-regulated	unknown protein (OJ1351_C05.18), mRNA
Os.12952.1.S1_at	6hr	3008.5	630.2	2.00	2.00E-05	1481.1	540.4	1.40	2.00E-05	Up-regulated	glycosyl hydrolases family 17 protein
LOC_Os01g71860	24hr	142	679.5	-2.60	2.00E-05	78.2	411.7	-2.10	3.89E-04	Down-regulated	
Os.26063.1.S1_at	6hr	1350.9	502.4	1.50	2.00E-05	796.3	218.6	1.80	2.00E-05	Up-regulated	expressed protein
LOC_Os11g15624	24hr	703.2	2086.6	-1.50	2.00E-05	427.8	3495.4	-3.00	2.00E-05	Down-regulated	
Os.409.1.S1_at	6hr	1306.8	300.5	2.40	2.00E-05	559.9	183.1	1.30	2.00E-05	Up-regulated	metal transporter Nramp6
LOC_Os07g15460	24hr	440	1349.5	-1.60	2.00E-05	211.1	2503.9	-3.60	2.00E-05	Down-regulated	
Os.48053.1.A1_at	6hr	626.6	104.5	2.90	2.00E-05	471.3	70.1	2.40	2.00E-05	Up-regulated	Transcribed sequences
	24hr	45.8	202.4	-2.10	1.20E-03	45.6	508.8	-3.20	2.00E-05	Down-regulated	
Os.51491.1.S1_at	6hr	781.3	207	2.00	2.00E-05	441.2	117.9	2.00	6.00E-05	Up-regulated	retrotransposon protein
LOC_Os01g31020	24hr	430.5	1415.1	-1.80	2.00E-05	203.2	1909.5	-3.30	2.00E-05	Down-regulated	
Os.55079.1.S1_at	6hr	1315.1	634.4	1.20	2.00E-05	1495.4	711.7	1.30	2.00E-05	Up-regulated	cDNA clone:002-128-A08, full insert sequence.
	24hr	53.1	206	-1.50	1.01E-04	2.8	200.7	-4.50	1.67E-04	Down-regulated	
Os.6101.1.S1_at	6hr	2892.3	938.5	1.70	2.00E-05	3102.3	1450.9	1.10	2.00E-05	Up-regulated	indole-3-acetate beta-glucosyltransferase
LOC_Os04g12970	24hr	16.4	512.8	-4.70	2.30E-05	44	306.1	-1.60	6.00E-05	Down-regulated	
Os.9805.1.S1_at	6hr	134.1	9.9	3.50	1.67E-04	55.9	8.4	3.40	1.42E-02	Up-regulated	expressed protein
LOC_Os12g18410	24hr	12.4	152.9	-3.70	3.50E-05	4.6	703.1	-6.50	2.00E-05	Down-regulated	
Os.10032.1.S1_at	6hr	162.8	151.7	0.30	3.91E-01	223.5	84.3	1.30	6.18E-04	Un-changed	rac-like GTP-binding protein 2
LOC_Os05g43820	24hr	311.3	186.4	1.10	4.00E-05	345.4	183.2	1.10	2.14E-04	Up-regulated	
Os.10141.1.S1_at	6hr	2123.2	1838.6	0.30	1.10E-02	2376.5	2169.8	0.20	2.67E-02	Un-changed	narf-like protein
LOC_Os03g53750	24hr	4606.1	1669.5	1.50	2.00E-05	4423.5	1570.6	1.60	2.00E-05	Up-regulated	
Os.10348.1.S1_at	6hr	692.8	402.4	0.80	2.00E-05	976.3	379.4	1.30	2.00E-05	Un-changed	flavonol-3-O-glycoside-7-O-glucosyltransferase 1
LOC_Os01g41430	24hr	437.3	229.6	1.10	2.30E-05	587	136.1	1.60	6.80E-05	Up-regulated	
Os.1054.1.A1_at	6hr	2027.8	1323	0.60	2.14E-04	1838.3	1038.9	0.90	6.00E-05	Un-changed	HGA6
LOC_Os01g02900	24hr	2612.6	900.1	1.60	2.00E-05	1627.4	709.5	1.20	1.67E-04	Up-regulated	

Probe Set ID/Locus ID	Time	Quinclorac vs. Mock Repeat 1				Quinclorac vs. Mock Repeat 2				Assignment	Description
		Quinclorac	Mock	Log Ratio	P-value	Quinclorac	Mock	Log Ratio	P-value		
Os.10942.1.S1_a_at	6hr	125.5	193.1	-0.30	1.10E-02	131	274.6	-0.90	1.01E-04	Un-changed	heat shock factor protein HSF30
LOC_Os10g28340	24hr	535.4	128.9	1.60	2.00E-05	313.9	108.9	1.20	1.67E-04	Up-regulated	
Os.11029.1.S1_at	6hr	7065.8	6754.5	0.00	5.00E-01	6403	6355.4	0.00	5.00E-01	Un-changed	heat shock cognate 70 kDa protein 2
LOC_Os03g16860	24hr	15549.2	4623.3	1.90	2.00E-05	12711	3848.3	1.60	2.00E-05	Up-regulated	
Os.11112.1.S1_s_at	6hr	7.2	5.1	0.20	5.00E-01	14.2	9.4	-0.40	9.43E-02	Un-changed	expressed protein
LOC_Os08g40910	24hr	174.4	10.6	4.00	2.30E-05	315.5	15.4	4.20	4.60E-05	Up-regulated	
Os.11266.1.S1_at	6hr	375.9	152.7	0.80	6.80E-05	430.3	247	1.10	2.00E-05	Un-changed	auxin-induced protein PCNT115
LOC_Os04g27060	24hr	463.5	143.7	1.40	2.70E-05	801.9	114.9	3.10	2.00E-05	Up-regulated	
Os.11639.1.S1_at	6hr	703.5	604.9	0.40	8.51E-03	934.4	616.8	0.50	6.18E-04	Un-changed	myristoyl-acyl carrier protein thioesterase,
LOC_Os06g05130	24hr	1655.2	680.6	1.00	2.00E-05	1650.4	815.1	1.00	2.00E-05	Up-regulated	chloroplast precursor
Os.11941.2.S1_at	6hr	194.6	303.6	-0.70	2.14E-04	130.8	428.3	-1.60	3.00E-05	Un-changed	heat shock factor protein 7
LOC_Os09g35790	24hr	1857.9	280.2	2.40	2.00E-05	1623.2	429.8	1.90	2.00E-05	Up-regulated	
Os.12023.1.S1_at	6hr	2535.8	1939.5	0.50	2.30E-05	3308.2	2223.7	0.70	2.00E-05	Un-changed	glutathione synthetase, chloroplast precursor
LOC_Os12g34380	24hr	4327.2	1873.7	1.20	2.00E-05	4500.2	2417.1	1.00	2.00E-05	Up-regulated	
Os.12063.1.S1_at	6hr	1701.4	1402.4	0.30	1.06E-01	2346.3	1527.6	0.60	2.00E-05	Un-changed	expressed protein
LOC_Os02g11859	24hr	1146.3	561.9	1.10	2.00E-05	1101.5	367.6	1.70	2.00E-05	Up-regulated	
Os.12162.1.S1_at	6hr	554.7	282.7	0.90	3.50E-05	522.5	258.8	1.20	3.00E-05	Un-changed	mitochondrial carnitine/acylcarnitine carrier-like
LOC_Os10g42299	24hr	809.2	336.6	1.70	2.00E-05	669.7	348.2	1.50	6.80E-05	Up-regulated	protein
Os.12240.1.S1_at	6hr	361.9	125.2	1.30	1.47E-04	272.9	183	0.70	1.49E-03	Un-changed	phi-1-like phosphate-induced protein
LOC_Os02g52010	24hr	820.9	238.7	1.80	2.00E-05	327.6	78.3	1.90	2.00E-05	Up-regulated	
Os.12591.2.S1_x_at	6hr	1814.9	1213.2	0.50	6.00E-05	1928.2	1402.8	0.60	2.00E-05	Un-changed	alcohol dehydrogenase 1
LOC_Os11g10480	24hr	2082.1	769.8	1.40	2.00E-05	2333.4	978.7	1.10	2.00E-05	Up-regulated	
Os.12693.3.S1_at	6hr	317.1	421.5	-0.50	2.25E-03	308.6	391	-0.30	1.77E-01	Un-changed	OsAPx2 - Cytosolic Ascorbate Peroxidase encoding
LOC_Os07g49400	24hr	605.8	297.2	1.10	2.00E-05	939.9	344.7	1.50	2.00E-05	Up-regulated	gene
Os.12738.1.S2_a_at	6hr	2954.2	2361.8	0.30	2.25E-03	3599.2	2298.3	0.50	2.00E-05	Un-changed	glutamate synthase, chloroplast precursor
LOC_Os01g48960	24hr	3245.4	1704.5	1.00	2.00E-05	3596	1845.7	1.00	2.30E-05	Up-regulated	
Os.12843.1.S1_at	6hr	438.8	499.1	0.00	5.00E-01	706	346.6	0.90	2.70E-05	Un-changed	peroxidase 52 precursor
LOC_Os03g22020	24hr	1234.7	375.2	1.80	2.30E-05	2946.9	578.2	2.30	3.00E-05	Up-regulated	
Os.12934.1.S1_at	6hr	79.2	101.5	-0.10	2.88E-02	327.3	60.7	2.60	4.00E-05	Un-changed	AMP binding protein
LOC_Os01g24030	24hr	204	87.8	1.50	8.65E-04	378.5	54.3	3.20	2.30E-05	Up-regulated	
Os.15830.1.S1_at	6hr	5511.6	3307.3	0.70	2.30E-05	6435.9	2549.4	1.20	2.00E-05	Un-changed	alanine--glyoxylate aminotransferase 2 homolog 2,
LOC_Os05g39770	24hr	16678.8	5765.5	1.50	2.00E-05	19733.7	6055.4	1.70	2.00E-05	Up-regulated	mitochondrial precursor
Os.16245.1.S1_at	6hr	298.1	446.2	-0.70	2.30E-05	219.5	421.8	-0.80	3.00E-05	Un-changed	heat shock protein binding protein
LOC_Os03g18200	24hr	1364.5	402.8	1.40	2.70E-05	1451.2	483.9	1.40	2.00E-05	Up-regulated	
Os.16317.1.S1_at	6hr	11.8	34.3	-1.80	2.67E-02	7.6	3.3	-0.50	3.31E-01	Un-changed	heat shock protein STI
LOC_Os04g45480	24hr	161.5	49.6	1.50	4.38E-04	218.5	38.9	2.50	1.14E-04	Up-regulated	
Os.16540.1.S1_at	6hr	1502.6	957.7	0.70	2.00E-05	1912.3	1067.6	0.90	1.67E-04	Un-changed	gibberellin receptor GID1L2
LOC_Os07g06830	24hr	1498.4	619.7	1.10	2.00E-05	1522.1	837.5	1.30	2.00E-05	Up-regulated	
Os.2010.1.S1_at	6hr	852.3	497	1.30	2.00E-05	862.1	631.5	0.80	3.50E-05	Un-changed	aldehyde dehydrogenase, mitochondrial precursor
LOC_Os01g40870	24hr	449	152.7	1.80	2.00E-05	518.5	75	2.80	2.00E-05	Up-regulated	
Os.20404.1.S1_at	6hr	612.2	358.8	0.60	1.67E-04	989.7	441.2	1.20	2.00E-05	Un-changed	phenazine biosynthesis protein
LOC_Os01g16146	24hr	1631.5	423.9	1.90	2.00E-05	3724.1	680.7	2.60	2.00E-05	Up-regulated	
Os.22731.1.S1_at	6hr	277.5	436.7	-1.00	8.90E-05	350.1	512	-0.80	6.00E-05	Un-changed	17.4 kDa class I heat shock protein 2
LOC_Os03g16020	24hr	1997.1	758.9	1.30	2.00E-05	1933.9	922.1	1.20	2.00E-05	Up-regulated	
Os.2292.3.S1_x_at	6hr	34.2	40	0.70	4.29E-01	60.9	43.8	0.40	5.00E-01	Un-changed	heat shock factor protein HSF8
LOC_Os03g53340	24hr	712.5	150.2	1.90	2.00E-05	452.4	195.1	1.10	2.00E-05	Up-regulated	
Os.2376.1.S1_at	6hr	370	330.7	0.50	8.51E-03	475.5	292.3	0.80	7.80E-05	Un-changed	IN2-1 protein
LOC_Os03g17480	24hr	892.2	231.1	2.10	2.00E-05	848.5	461.8	2.20	4.00E-05	Up-regulated	
Os.24644.1.S1_at	6hr	229.2	164.6	0.60	1.20E-02	191.3	161.9	0.50	2.29E-02	Un-changed	ABC transporter family protein
LOC_Os04g49890	24hr	414	226.7	1.10	3.00E-05	378.3	145.2	2.00	7.80E-05	Up-regulated	
Os.24696.1.A1_s_at	6hr	322.3	652.1	-0.80	6.80E-05	331.4	755.9	-0.90	6.00E-05	Un-changed	WD-repeat protein 26
LOC_Os10g32770	24hr	835.6	493.4	1.00	2.00E-05	707	467.3	1.00	2.00E-05	Up-regulated	
Os.25621.2.S1_at	6hr	3786.2	1692.1	1.20	2.00E-05	5075.2	2093.9	0.90	2.00E-05	Un-changed	cytochrome P450 71A1

Probe Set ID/Locus ID	Time	Quinclorac vs. Mock Repeat 1				Quinclorac vs. Mock Repeat 2				Assignment	Description
		Quinclorac	Mock	Log Ratio	P-value	Quinclorac	Mock	Log Ratio	P-value		
LOC_Os12g16720	24hr	2155.9	1498.8	1.10	7.80E-05	3381.5	914.8	1.70	2.00E-05	Up-regulated	
Os.25736.1.S1_at	6hr	1811.8	1739.1	0.00	5.00E-01	2173.7	1335	0.60	2.00E-05	Un-changed	heat shock 70 kDa protein, mitochondrial precursor
LOC_Os09g31486	24hr	1651	670.3	1.10	2.70E-05	2504.2	531.3	2.20	2.00E-05	Up-regulated	
Os.26695.1.S1_at	6hr	3247.4	4876.4	-0.50	2.00E-05	3243.6	5763.6	-0.70	2.00E-05	Un-changed	NAC domain-containing protein 67
LOC_Os03g60080	24hr	9018.6	4146.7	1.20	2.00E-05	9611.3	4011.3	1.20	2.00E-05	Up-regulated	
Os.26955.1.S1_at	6hr	584.6	1318.1	-0.80	1.30E-04	1155	1477.4	-0.50	1.89E-04	Un-changed	expressed protein
LOC_Os08g39150	24hr	4053.4	1734.4	1.20	3.50E-05	4405.8	2040.6	1.10	3.50E-05	Up-regulated	
Os.27176.1.S1_at	6hr	984.2	978.2	-0.10	5.00E-01	1104.6	1164.9	0.20	1.20E-03	Un-changed	heat shock factor protein 4
LOC_Os09g28354	24hr	862.3	514.6	1.30	2.00E-05	1200.9	627.4	1.40	2.00E-05	Up-regulated	
Os.27657.1.A1_at	6hr	831.7	776.9	0.20	5.00E-01	1414.8	998.3	0.60	1.01E-04	Un-changed	ABC transporter family protein
LOC_Os04g49890	24hr	2197.3	847.5	1.30	2.00E-05	2713.3	1256.6	1.00	2.00E-05	Up-regulated	
Os.27824.1.S1_at	6hr	1705.9	1783.6	0.00	5.00E-01	2295.3	1474.4	0.40	4.00E-05	Un-changed	peptidase/ serine-type peptidase
LOC_Os09g28000	24hr	3236.2	1493.4	1.10	2.00E-05	3130.3	1321.4	1.30	2.00E-05	Up-regulated	
Os.28217.1.S1_at	6hr	74.3	53.9	0.30	2.43E-01	86.6	52.1	0.20	4.68E-01	Un-changed	expressed protein
LOC_Os01g62810	24hr	1765	683.3	1.40	2.00E-05	1218.5	647.6	1.10	2.00E-05	Up-regulated	
Os.313.1.S1_a_at	6hr	1264.9	1455	-0.20	1.01E-02	1982	1604.3	0.20	5.00E-01	Un-changed	aldehyde dehydrogenase, mitochondrial precursor
LOC_Os02g49720	24hr	3462.4	982	1.70	2.00E-05	7204	1182	2.60	2.00E-05	Up-regulated	
Os.3427.1.S1_at	6hr	1112.7	732.5	0.60	2.00E-05	1347.2	670.9	1.00	2.00E-05	Un-changed	NADPH oxidase
LOC_Os01g53294	24hr	2323.2	723.8	1.50	2.00E-05	2170.3	941.5	1.10	2.00E-05	Up-regulated	
Os.35463.1.S1_at	6hr	360.7	371.7	0.10	5.00E-01	335	260.1	0.00	5.00E-01	Un-changed	dnaJ homolog subfamily B member 5
LOC_Os05g48810	24hr	1730.4	316.5	2.80	2.00E-05	1031	264.2	2.30	5.20E-05	Up-regulated	
Os.36449.1.S1_at	6hr	69.7	38.3	0.10	2.12E-02	274.5	122.3	1.20	2.67E-02	Un-changed	indole-3-acetic acid-amido synthetase GH3.1
LOC_Os01g57610	24hr	337.2	19.4	3.30	7.74E-04	489.4	97.2	2.30	4.00E-05	Up-regulated	
Os.37217.1.S1_at	6hr	126.7	110.5	-0.20	5.00E-01	257.1	184	0.50	5.00E-01	Un-changed	expressed protein
LOC_Os03g60760	24hr	786.8	282.2	1.50	4.60E-05	634.3	297.4	1.00	2.00E-05	Up-regulated	
Os.37238.1.S1_at	6hr	362.5	904.2	-1.20	1.67E-04	531.8	766.2	-0.90	7.80E-05	Un-changed	nuclear transcription factor Y subunit A-3
LOC_Os08g09690	24hr	2081.1	1274.8	1.10	2.00E-05	2389.1	1313	1.00	2.00E-05	Up-regulated	
Os.37548.1.S1_at	6hr	1712.4	1132	0.70	2.70E-05	2098.6	1284.1	0.60	2.30E-05	Un-changed	NAC domain-containing protein 48
LOC_Os05g34830	24hr	2523.7	961.2	1.20	2.00E-05	5212.4	1539.1	1.50	2.00E-05	Up-regulated	
Os.37616.1.S1_at	6hr	1689.2	2487	0.30	6.15E-02	1511.1	1037.6	0.60	2.25E-03	Un-changed	heat shock protein 81-1
LOC_Os08g39140	24hr	7809.5	2945.7	1.20	2.30E-05	7569.1	1634.4	1.70	4.60E-05	Up-regulated	
Os.39876.1.S1_at	6hr	175	207.1	-0.30	1.10E-02	176.1	233.4	-0.40	7.74E-04	Un-changed	heat shock factor protein 4
LOC_Os04g48030	24hr	969.9	132.2	2.90	2.00E-05	475.9	156.6	1.90	2.00E-05	Up-regulated	
Os.40021.1.S1_a_at	6hr	99.8	124.9	0.30	1.77E-01	121.4	69.3	0.40	5.00E-01	Un-changed	heat shock factor protein 1
LOC_Os03g06630	24hr	938.7	120.3	3.20	2.00E-05	531.4	121.9	2.10	2.00E-05	Up-regulated	
Os.46024.1.S1_at	6hr	677.3	1094.4	-0.60	2.00E-05	743.8	752.9	-0.10	5.00E-01	Un-changed	heat shock cognate 70 kDa protein
LOC_Os01g62290	24hr	7901	2406.9	1.50	2.00E-05	8306.7	2276.6	1.50	2.00E-05	Up-regulated	
Os.46328.1.S1_at	6hr	118.9	86.9	0.50	1.31E-02	378	180.7	0.90	2.41E-04	Un-changed	cis-zeatin O-glucosyltransferase 1
LOC_Os04g46980	24hr	261.8	64.7	2.20	2.14E-04	448.3	189.6	1.40	4.92E-04	Up-regulated	
Os.46328.1.S1_x_at	6hr	366	244.7	0.60	4.07E-03	833.3	424.2	0.70	3.07E-04	Un-changed	cis-zeatin O-glucosyltransferase 1
LOC_Os04g46980	24hr	1009.7	181.5	2.10	4.00E-05	952.8	294.1	1.40	6.00E-05	Up-regulated	
Os.46667.1.S1_x_at	6hr	550	812.4	-0.80	5.20E-05	655.3	1025.7	-0.80	3.46E-04	Un-changed	WD-repeat protein 26
LOC_Os10g32770	24hr	1127.1	462.9	1.10	2.00E-05	1074.2	633	1.00	2.00E-05	Up-regulated	
Os.4697.1.S1_at	6hr	798.7	751.8	0.30	2.12E-02	1008.4	647.4	0.80	1.34E-03	Un-changed	glucose-6-phosphate isomerase, cytosolic B
LOC_Os06g14510	24hr	1377.4	655.1	1.00	2.00E-05	1754.8	928.9	1.10	2.00E-05	Up-regulated	
Os.48724.1.S1_at	6hr	940.7	936	0.00	5.00E-01	1348.8	619	1.10	2.00E-05	Un-changed	protein HOTHEAD precursor
LOC_Os08g31030	24hr	1782.9	631.3	1.50	2.00E-05	1579.6	755.6	1.00	2.00E-05	Up-regulated	
Os.4891.1.S1_at	6hr	7390	4354.7	0.80	2.00E-05	7565.2	3446.7	1.10	2.00E-05	Un-changed	phosphoserine aminotransferase, chloroplast precursor
LOC_Os03g06200	24hr	6820.6	2819.9	1.00	2.00E-05	6614.8	2314.9	1.40	2.00E-05	Up-regulated	
Os.49074.1.A1_at	6hr	159.8	285.2	-0.50	4.48E-03	388.1	494	-0.40	4.48E-03	Un-changed	hypothetical protein
LOC_Os07g37290	24hr	459.1	235.4	1.40	6.18E-04	718.9	338.3	1.00	7.80E-05	Up-regulated	
Os.49534.1.S1_at	6hr	314.9	216.6	0.70	3.50E-05	450.5	241.1	1.50	1.01E-04	Un-changed	early nodulin-like protein 1 precursor
LOC_Os08g17160	24hr	932.8	214.4	1.50	6.00E-05	945.2	432.6	1.10	6.00E-05	Up-regulated	

Probe Set ID/Locus ID	Time	Quinclorac vs. Mock Repeat 1				Quinclorac vs. Mock Repeat 2				Assignment	Description
		Quinclorac	Mock	Log Ratio	P-value	Quinclorac	Mock	Log Ratio	P-value		
Os.49534.1.S1_x_at	6hr	347.3	296.6	0.70	6.18E-04	522	111.3	2.00	4.00E-05	Un-changed	early nodulin-like protein 1 precursor
LOC_Os08g17160	24hr	1015.5	325.7	1.70	3.00E-05	939.5	438.5	1.10	2.00E-05	Up-regulated	
Os.49591.1.S1_at	6hr	508.1	553.9	0.30	6.56E-02	1134.2	691.3	0.50	8.80E-05	Un-changed	metal ion binding protein
LOC_Os03g05750	24hr	1826.1	342.6	2.90	2.30E-05	1102.2	267.1	1.70	2.00E-05	Up-regulated	
Os.50281.1.S1_at	6hr	235.3	174.9	0.50	3.46E-04	279.4	194.6	0.40	3.46E-04	Un-changed	actin binding protein
LOC_Os07g39920	24hr	459.3	200.5	1.30	2.00E-05	387.3	231.9	1.10	1.01E-04	Up-regulated	
Os.50642.1.S1_at	6hr	61.2	132.7	-0.60	9.99E-02	59	90.2	0.00	5.00E-01	Un-changed	heat shock factor protein 4
LOC_Os04g48030	24hr	394.2	39.4	3.10	4.00E-05	193.1	79.9	1.50	2.41E-04	Up-regulated	
Os.51021.1.S1_at	6hr	135	86.1	0.70	3.10E-02	177.4	80.3	1.20	3.46E-04	Un-changed	DNA binding protein
LOC_Os04g52920	24hr	228.8	91.4	1.40	4.00E-05	408.6	101	2.00	2.00E-05	Up-regulated	
Os.51063.1.S1_at	6hr	733.6	304.8	1.40	2.00E-05	310.1	227.3	0.60	1.81E-02	Un-changed	DNA binding protein
LOC_Os09g28210	24hr	890.8	242.6	1.90	2.00E-05	540.5	205.2	1.70	4.60E-05	Up-regulated	
Os.52329.1.S1_at	6hr	762.3	452.9	0.60	2.70E-05	796.4	418.1	1.10	2.70E-05	Un-changed	thiosulfate transferase
LOC_Os02g07044	24hr	1162.2	449.6	1.40	2.00E-05	1678.3	473.9	1.90	2.00E-05	Up-regulated	
Os.52498.1.S1_at	6hr	642.5	459.3	0.40	3.10E-02	737.3	446.8	0.40	1.89E-04	Un-changed	asp/Glu racemase
LOC_Os01g37470	24hr	1968.2	564	1.80	2.00E-05	1809.6	580.4	1.50	2.00E-05	Up-regulated	
Os.52733.1.S1_at	6hr	146.8	257.1	-0.70	4.07E-03	129.7	174.9	-0.50	1.69E-01	Un-changed	disulfide oxidoreductase/ monooxygenase/
LOC_Os04g03980	24hr	208.8	148.8	1.00	2.73E-04	340.2	163.1	1.00	4.93E-03	Up-regulated	oxidoreductase
Os.55368.1.S1_at	6hr	98.1	139.6	-0.20	5.00E-01	137.4	145.3	0.00	3.79E-01	Un-changed	expressed protein
LOC_Os05g05290	24hr	228.7	54.5	2.10	5.52E-04	122.4	28.9	2.40	3.00E-05	Up-regulated	
Os.5549.1.S1_at	6hr	2610.7	1360	1.10	2.00E-05	5389	2654.2	0.80	2.00E-05	Un-changed	NAC domain-containing protein 67
LOC_Os07g12340	24hr	3667.9	944.3	1.90	2.00E-05	4868.1	1143.2	2.00	2.00E-05	Up-regulated	
Os.55682.1.S1_at	6hr	566.5	344.3	0.60	1.67E-04	1007.9	482.4	1.00	2.00E-05	Un-changed	expressed protein
LOC_Os03g22820	24hr	486.3	154.1	1.60	2.30E-05	1163.6	158.1	2.60	2.00E-05	Up-regulated	
Os.5574.1.S1_s_at	6hr	124.1	186	-0.50	1.10E-02	47.9	124.8	-0.90	4.92E-03	Un-changed	chaperone clpB 1
LOC_Os02g08490	24hr	746.5	311.6	1.10	2.00E-05	750.6	279.9	1.50	2.00E-05	Up-regulated	
Os.56145.1.S1_at	6hr	333.5	168.8	0.70	6.92E-04	386.5	353.8	0.10	5.00E-01	Un-changed	glycine-rich cell wall structural protein 2
LOC_Os12g13890	24hr	143.2	60.7	1.20	3.89E-04	305.1	11.9	4.20	1.47E-04	Up-regulated	precursor
Os.6162.1.S1_at	6hr	749.8	1590.1	-1.10	2.00E-05	1154	1572.3	-0.50	2.00E-05	Un-changed	expressed protein
LOC_Os02g04650	24hr	5604.7	2328.6	1.20	2.00E-05	6192.8	2569.5	1.10	2.00E-05	Up-regulated	
Os.6242.1.S1_s_at	6hr	505.8	528.4	0.10	4.94E-01	427.5	325.9	0.70	8.80E-05	Un-changed	expressed protein
LOC_Os07g41600	24hr	708.3	522.1	1.20	2.00E-05	798.4	416.2	1.00	7.80E-05	Up-regulated	
Os.6452.1.S1_at	6hr	92.7	55.6	1.00	2.03E-03	154.7	41.7	0.80	8.89E-02	Un-changed	cytochrome P450 72A18
LOC_Os01g43710	24hr	193.4	71.7	1.90	6.00E-05	317.7	57.9	2.30	1.30E-04	Up-regulated	
Os.6585.1.S1_at	6hr	3591.3	3179.9	0.10	5.00E-01	4104.3	2961.6	0.50	2.00E-05	Un-changed	hydroxyacid oxidase 1
LOC_Os04g53210	24hr	7617.6	2116	1.70	2.00E-05	11499.6	3582.2	2.10	2.00E-05	Up-regulated	
Os.681.1.S1_at	6hr	187.7	277.7	0.00	5.00E-01	544.7	447.4	1.00	6.80E-05	Un-changed	4-methyl-5-thiazole monophosphate biosynthesis
LOC_Os01g11860	24hr	389.1	187.3	1.00	2.30E-05	536.1	198.6	1.00	5.20E-05	Up-regulated	protein
Os.7102.1.S1_at	6hr	2618.3	2433.3	0.10	5.00E-01	3256.6	2519.7	0.40	2.00E-05	Un-changed	cysteine synthase
LOC_Os03g53650	24hr	4669.9	2472	1.00	2.00E-05	5480.3	2506.8	1.10	2.00E-05	Up-regulated	
Os.7366.1.S1_at	6hr	415.6	358.1	0.30	2.67E-02	421.5	234.6	0.50	1.65E-03	Un-changed	expressed protein
LOC_Os05g38270	24hr	4384.3	1536.2	1.70	2.00E-05	2959.8	1437.7	1.00	2.00E-05	Up-regulated	
Os.7664.1.S1_at	6hr	2770.4	2046.8	0.40	6.92E-04	2850	1983.2	0.50	3.50E-05	Un-changed	thioredoxin
LOC_Os02g56900	24hr	8392.5	1862.8	2.10	2.00E-05	7098.3	1962.3	1.80	2.00E-05	Up-regulated	
Os.7794.1.S1_at	6hr	1432.3	1001.1	0.60	3.89E-04	1849.6	1007.4	1.10	3.50E-05	Un-changed	glutathione S-transferase GSTU6
LOC_Os10g38501	24hr	2916.8	763.5	1.30	2.00E-05	3679.7	1121.3	1.50	2.00E-05	Up-regulated	
Os.8113.1.S1_at	6hr	700.2	428.6	0.60	1.67E-04	667.9	169.3	1.50	2.00E-05	Un-changed	protein binding protein
LOC_Os01g08470	24hr	1382.1	591	1.30	2.00E-05	1332.7	712	1.10	2.00E-05	Up-regulated	
Os.8481.2.S1_at	6hr	210	246.2	-0.10	5.40E-02	112.7	296	-0.70	2.12E-02	Un-changed	expressed protein
LOC_Os03g13450	24hr	1059.2	273.7	2.00	2.00E-05	849.3	309.8	1.80	2.00E-05	Up-regulated	
Os.8481.2.S1_x_at	6hr	118.6	203.5	-0.30	7.43E-02	121.4	121	-0.20	2.33E-01	Un-changed	expressed protein
LOC_Os03g13450	24hr	863.2	237.4	1.70	1.67E-04	484.3	144.4	1.10	2.49E-03	Up-regulated	
Os.8585.1.S1_at	6hr	8077.3	6843.5	0.20	4.81E-01	10210.2	6242.1	0.70	2.00E-05	Un-changed	OsIAA13 - Auxin-responsive Aux/IAA gene family

Probe Set ID/Locus ID	Time	Quinclorac vs. Mock Repeat 1				Quinclorac vs. Mock Repeat 2				Assignment	Description
		Quinclorac	Mock	Log Ratio	P-value	Quinclorac	Mock	Log Ratio	P-value		
LOC_Os03g53150	24hr	10785.6	4555.1	1.20	2.00E-05	11831.3	5688.7	1.10	2.00E-05	Up-regulated	member
Os.8765.1.S1_a_at	6hr	2641	1810.2	0.30	3.70E-03	3168	1969	0.40	1.47E-04	Un-changed	hydroxyacylglutathione hydrolase 3, mitochondrial precursor
LOC_Os01g47690	24hr	3143.4	1789.4	1.10	2.00E-05	5219.1	1661.5	1.50	2.00E-05	Up-regulated	
Os.9558.1.S1_s_at	6hr	393.2	352.5	0.10	5.00E-01	342.6	299.9	0.20	5.40E-02	Un-changed	protein binding protein
LOC_Os02g45780	24hr	528	266.6	1.20	3.00E-05	740	316.4	1.50	5.20E-05	Up-regulated	
OsAffx.11075.1.S1_x_at	6hr	193.4	215.8	0.30	4.41E-02	234.5	241.5	0.70	1.67E-02	Un-changed	phenazine biosynthesis protein
LOC_Os01g16146	24hr	471.3	289.6	1.00	7.80E-05	684.3	300.4	1.70	3.50E-05	Up-regulated	
OsAffx.12089.1.S1_at	6hr	52.2	104.4	-1.00	5.00E-01	156.2	188	-0.10	1.95E-01	Un-changed	expressed protein
LOC_Os02g17620	24hr	333.2	108.9	1.50	2.00E-05	632.3	273.1	1.60	2.00E-05	Up-regulated	
OsAffx.23903.2.S1_s_at	6hr	202.9	237	-0.10	5.00E-01	209.5	260.6	-0.40	4.42E-01	Un-changed	hypothetical protein
LOC_Os01g63490	24hr	561.1	237.3	1.20	2.00E-05	477.3	181	1.00	3.50E-05	Up-regulated	
OsAffx.25718.1.S1_at	6hr	911.9	646.8	0.70	1.30E-04	962	461.4	1.00	2.14E-04	Un-changed	cytochrome P450 CYP81A7
LOC_Os03g55250	24hr	1218.7	701.9	1.20	2.00E-05	1393.1	616.3	1.00	2.00E-05	Up-regulated	
OsAffx.29871.1.S1_x_at	6hr	4525.9	2735.1	0.70	2.00E-05	5400.8	2687.7	1.10	2.00E-05	Un-changed	PDR5-like ABC transporter
LOC_Os09g16330	24hr	4222.9	1646.5	1.40	2.00E-05	7930.6	1411.3	2.50	2.00E-05	Up-regulated	
OsAffx.30145.1.S1_at	6hr	204.7	185.7	0.20	5.00E-01	158.3	203.7	-0.50	1.67E-02	Un-changed	heat shock factor protein 7
LOC_Os09g35790	24hr	683.5	111.3	2.30	1.49E-03	635.3	144.3	1.30	1.67E-02	Up-regulated	
OsAffx.32239.1.S1_at	6hr	1196.4	351.2	1.50	2.00E-05	286.2	186.6	0.40	2.23E-01	Un-changed	NADH dehydrogenase subunit I
LOC_Osp1g00950	24hr	465.7	183.9	1.00	2.00E-05	343.6	114.3	1.30	4.60E-05	Up-regulated	
Os.12257.1.S1_at	6hr	158.2	630.7	-1.80	2.30E-05	260.7	599.2	-1.10	1.01E-04	Down-regulated	heat shock 22 kDa protein, mitochondrial precursor
LOC_Os02g52150	24hr	3524.6	876.4	2.10	2.00E-05	2923.1	822.3	1.70	2.00E-05	Up-regulated	
Os.54707.1.S1_x_at	6hr	36	157	-1.90	2.14E-04	27.9	103.4	-1.60	2.67E-02	Down-regulated	heat shock factor protein 7
LOC_Os09g35790	24hr	474.2	187.1	1.30	1.14E-04	411.2	60.7	2.10	3.50E-05	Up-regulated	
Os.5698.1.S1_s_at	6hr	161.4	341.4	-1.00	4.92E-03	146.4	366.9	-1.50	2.73E-04	Down-regulated	LEC14B
LOC_Os05g33710	24hr	2409.1	1056	1.10	2.00E-05	2782.2	1061.8	1.60	2.00E-05	Up-regulated	
Os.9950.1.S1_at	6hr	71.1	233.8	-1.40	2.14E-04	77.6	266.6	-2.10	7.74E-04	Down-regulated	17.4 kDa class I heat shock protein 3
LOC_Os06g14240	24hr	981.8	269	1.90	2.00E-05	1083.7	302.9	1.90	4.60E-05	Up-regulated	
Os.10689.1.S1_at	6hr	302.3	776.7	-1.30	2.00E-05	393.3	1266.4	-1.50	2.00E-05	Down-regulated	cytochrome P450 88A1
LOC_Os06g02019	24hr	414.7	894.4	-1.10	2.00E-05	576.7	1368.4	-1.10	2.00E-05	Down-regulated	
Os.11897.1.S1_at	6hr	3990.4	10921.3	-1.30	2.00E-05	5035.5	15955.3	-1.70	2.00E-05	Down-regulated	expressed protein
LOC_Os03g61150	24hr	416.5	2611.1	-2.60	2.00E-05	2356.3	5711.6	-1.10	2.00E-05	Down-regulated	
Os.12340.1.S1_at	6hr	4356.9	10151.1	-1.20	2.00E-05	3962	7909.7	-1.00	2.00E-05	Down-regulated	esterase precursor
LOC_Os01g11730	24hr	2557.1	8900.1	-1.80	2.00E-05	764.2	6427.1	-3.30	2.00E-05	Down-regulated	
Os.12528.1.S1_x_at	6hr	233.7	945.3	-2.10	2.00E-05	263.6	1595	-2.70	2.00E-05	Down-regulated	pyruvate, phosphate dikinase, chloroplast precursor
LOC_Os03g31750	24hr	231	494.7	-1.00	2.30E-05	272.9	611.2	-1.00	5.20E-05	Down-regulated	
Os.25556.1.S1_x_at	6hr	1399.2	3070.6	-1.10	2.00E-05	1824.3	3582.2	-1.00	2.00E-05	Down-regulated	transposon protein, CACTA, En/Spm sub-class
LOC_Os08g28350	24hr	568	2199.4	-1.60	2.00E-05	899.4	2110.6	-1.20	2.00E-05	Down-regulated	
Os.27520.4.S1_a_at	6hr	492.1	1691.8	-1.90	2.00E-05	478.6	2669.6	-2.60	2.00E-05	Down-regulated	F-box domain containing protein
LOC_Os10g03850	24hr	513	2117.9	-2.10	2.00E-05	1190.5	2445.3	-1.00	2.00E-05	Down-regulated	
Os.27520.8.S1_x_at	6hr	201	476.2	-1.80	3.50E-05	168.6	720.8	-2.20	2.00E-05	Down-regulated	retrotransposon protein
LOC_Os12g08564	24hr	232.9	1006.9	-2.50	2.00E-05	396.5	882.9	-1.20	2.00E-05	Down-regulated	
Os.46029.1.A1_at	6hr	211.4	1247.4	-2.40	2.00E-05	395.8	1362.8	-2.30	2.00E-05	Down-regulated	hypothetical protein
LOC_Os01g48940	24hr	669	1364.5	-1.00	2.00E-05	366.5	1182	-1.50	2.00E-05	Down-regulated	
Os.53009.1.A1_x_at	6hr	140.6	323.5	-1.10	4.38E-04	191.8	200.4	-1.00	3.46E-04	Down-regulated	glutamate receptor 2.9 precursor
LOC_Os02g54640	24hr	97.4	345.7	-1.30	1.89E-04	55	182.5	-3.50	3.70E-03	Down-regulated	
Os.53098.1.S1_at	6hr	14.1	141.3	-2.00	2.30E-05	47.9	288.1	-1.90	6.80E-05	Down-regulated	expressed protein
LOC_Os06g48500	24hr	42.8	137.1	-1.80	2.25E-03	65.1	196.2	-1.10	4.92E-04	Down-regulated	
Os.53137.1.S1_at	6hr	88	284.3	-1.90	2.00E-05	38.6	189.7	-2.10	1.83E-03	Down-regulated	retrotransposon protein
LOC_Os07g09300	24hr	67.9	618.7	-2.80	2.00E-05	60.4	503.4	-3.50	2.00E-05	Down-regulated	
Os.54355.1.S1_at	6hr	101.7	168.4	-1.10	8.89E-02	73.2	256.4	-1.90	1.14E-04	Down-regulated	verticillium wilt disease resistance protein
LOC_Os12g10870	24hr	137.6	421.1	-1.60	1.14E-04	105.2	273.4	-1.30	8.90E-05	Down-regulated	
Os.55618.1.S1_at	6hr	550.5	1472.7	-1.20	4.38E-04	642.5	1511	-1.00	2.00E-05	Down-regulated	organic cation transporter 3
LOC_Os07g37454	24hr	243.5	593.3	-1.10	7.74E-04	233.1	464.7	-1.00	2.70E-05	Down-regulated	

Probe Set ID/Locus ID	Time	Quinclorac vs. Mock Repeat 1				Quinclorac vs. Mock Repeat 2				Assignment	Description
		Quinclorac	Mock	Log Ratio	P-value	Quinclorac	Mock	Log Ratio	P-value		
OsAffx.26230.1.S1_at	6hr	552.8	1786	-1.90	2.00E-05	747.1	3729.9	-2.60	2.00E-05	Down-regulated	ripening-related protein 1 precursor
LOC_Os04g29550	24hr	1055.8	3511.4	-1.80	2.00E-05	1022	3159.8	-1.80	2.00E-05	Down-regulated	
Os.10174.1.S1_at	6hr	499.9	764.2	-1.10	2.00E-05	494	874.5	-1.00	2.00E-05	Down-regulated	MYB59
LOC_Os11g47460	24hr	1466.5	1299.6	0.20	1.01E-02	1132.7	1120.2	0.00	5.00E-01	Un-changed	
Os.10180.1.S1_at	6hr	321.4	672.1	-1.10	5.20E-05	320.7	625.4	-1.20	8.90E-05	Down-regulated	expressed protein
LOC_Os11g29140	24hr	556.5	602.7	-0.20	1.69E-01	553.2	611.9	-0.30	5.93E-03	Un-changed	
Os.10214.1.S1_at	6hr	2252.5	5020.5	-1.10	2.00E-05	2549.1	6399	-1.30	2.00E-05	Down-regulated	expressed protein
LOC_Os01g69870	24hr	4863.3	6606.3	-0.50	3.50E-05	6052.6	7654.5	-0.30	6.92E-04	Un-changed	
Os.10255.1.S1_s_at	6hr	146.8	533.6	-2.40	2.70E-05	130.9	961.8	-3.00	2.00E-05	Down-regulated	retrotransposon protein, Ty3-gypsy subclass
LOC_Os01g37350	24hr	245.1	726.3	-1.70	2.00E-05	584	734.9	-0.30	4.41E-02	Un-changed	
Os.10379.1.S1_at	6hr	236.7	559.9	-1.60	2.00E-05	188.3	659.7	-1.80	2.00E-05	Down-regulated	ABA induced plasma membrane protein PM 19
LOC_Os02g49860	24hr	387.5	614.3	-0.90	7.74E-04	336.7	572.8	-0.80	2.00E-05	Un-changed	
Os.10502.1.S1_at	6hr	608.5	1540.5	-1.20	2.00E-05	755	1756.4	-1.40	2.00E-05	Down-regulated	expressed protein
LOC_Os07g28614	24hr	572.1	747.6	-0.60	2.00E-05	489.9	597.6	-0.40	2.88E-02	Un-changed	
Os.10580.1.S1_at	6hr	315.2	758.9	-1.40	2.30E-05	252.1	547.2	-1.10	3.46E-04	Down-regulated	expressed protein
LOC_Os01g34790	24hr	638.7	387.6	0.70	8.80E-05	241.2	416.1	-0.90	2.41E-04	Un-changed	
Os.10736.1.S1_at	6hr	2347.9	6630.5	-1.30	2.00E-05	2345.3	6579.7	-1.60	2.00E-05	Down-regulated	expressed protein
LOC_Os06g05440	24hr	5714.2	7866.9	-0.40	2.00E-05	4697.6	6478.9	-0.50	2.00E-05	Un-changed	
Os.11327.1.S1_at	6hr	6026.5	16330.3	-1.50	2.00E-05	5140	16034.9	-2.00	2.00E-05	Down-regulated	asparagine synthetase
LOC_Os03g18130	24hr	13111	18409.5	-1.00	2.00E-05	13080	18097.9	-0.90	2.00E-05	Un-changed	
Os.11404.1.S1_s_at	6hr	1198.1	2378.6	-1.10	2.00E-05	1182.9	2499.8	-1.20	2.00E-05	Down-regulated	WRKY transcription factor 4
LOC_Os08g17400	24hr	1483.7	1951.6	-0.30	3.70E-03	1565	2286.1	-0.40	2.00E-05	Un-changed	
Os.11450.1.S1_at	6hr	617.9	1455.5	-1.40	2.00E-05	527.7	1742.6	-1.70	2.00E-05	Down-regulated	RING finger and CHY zinc finger domain-containing protein 1
LOC_Os01g52110	24hr	1863.5	3757.9	-1.10	2.00E-05	3285.1	3020.7	0.10	2.13E-01	Un-changed	
Os.11450.1.S3_a_at	6hr	2157.7	4196.6	-1.00	2.00E-05	1610.8	4709.3	-1.60	2.00E-05	Down-regulated	RING finger and CHY zinc finger domain-containing protein 1
LOC_Os01g52110	24hr	6084.5	10638.2	-0.90	2.00E-05	9416.8	11744.9	-0.30	1.49E-03	Un-changed	
Os.11608.1.S1_at	6hr	953.1	2416.7	-1.00	2.00E-05	1288	3083.7	-1.30	2.00E-05	Down-regulated	OsIAA6 - Auxin-responsive Aux/IAA gene family member
LOC_Os01g53880	24hr	2240.1	2590.4	-0.30	3.50E-05	3169.5	3129.8	0.00	5.00E-01	Un-changed	
Os.11611.1.S1_at	6hr	674.5	2013.8	-1.60	2.00E-05	789.5	2257.5	-1.50	2.00E-05	Down-regulated	11668.m01231
LOC_Os02g13350	24hr	1608.1	2820.1	-0.80	2.00E-05	2238.2	3236.6	-0.50	2.00E-05	Un-changed	
Os.11969.1.S1_at	6hr	2393	5594.8	-1.10	2.00E-05	2516.7	5313.4	-1.00	2.00E-05	Down-regulated	expressed protein
LOC_Os05g01530	24hr	11817.1	14783	-0.30	2.14E-04	12738.7	15112.3	-0.20	1.20E-02	Un-changed	
Os.11997.1.S1_at	6hr	2963.6	5560.8	-1.40	2.00E-05	2428.7	6629.1	-1.50	2.00E-05	Down-regulated	thioredoxin-like 1
LOC_Os07g48510	24hr	4695.3	4667.2	0.20	1.25E-01	4357.6	4707.9	0.00	5.00E-01	Un-changed	
Os.12244.1.S1_at	6hr	513	2395.9	-2.00	2.00E-05	628.1	3118.2	-2.30	2.00E-05	Down-regulated	17.4 kDa class I heat shock protein 3
LOC_Os03g15960	24hr	2302	4597.6	-1.00	2.00E-05	7560.9	4950.4	0.60	2.30E-05	Un-changed	
Os.12342.1.S1_at	6hr	1590.6	4363.1	-1.40	2.00E-05	1798.6	7168.5	-2.00	2.00E-05	Down-regulated	expressed protein
LOC_Os12g04204	24hr	5602.8	6199.4	-0.10	5.00E-01	6617.2	7236.1	0.00	5.00E-01	Un-changed	
Os.12342.1.S2_at	6hr	1765.6	3750.7	-1.10	2.00E-05	2115.7	7183.7	-1.70	2.00E-05	Down-regulated	expressed protein
LOC_Os12g04204	24hr	5488.9	6725.2	-0.20	1.83E-03	6901.9	8551.2	-0.40	3.00E-05	Un-changed	
Os.12664.1.S1_at	6hr	601.2	1622.4	-1.40	3.00E-05	631.7	1788.2	-1.40	2.30E-05	Down-regulated	serine/threonine kinase-like protein
LOC_Os07g35810	24hr	807	1521.5	-0.80	2.30E-05	324.3	1273.4	-1.90	3.07E-04	Un-changed	
Os.12977.1.S1_at	6hr	156.7	354.2	-1.10	7.80E-05	218.4	440.3	-1.30	1.89E-04	Down-regulated	CCT motif family protein
LOC_Os06g19444	24hr	177.9	190.7	-0.30	5.00E-01	164.7	178.3	-0.40	2.96E-01	Un-changed	
Os.1307.1.S1_a_at	6hr	276	729.6	-1.50	2.00E-05	441	762.3	-1.20	1.30E-04	Down-regulated	phytosulfokines 2 precursor
LOC_Os11g05190	24hr	187.9	239.2	-0.50	1.42E-02	173.9	289.8	-0.90	6.92E-04	Un-changed	
Os.13646.1.S1_at	6hr	117	666.8	-2.20	2.00E-05	166.2	570.6	-1.60	2.00E-05	Down-regulated	expressed protein
LOC_Os09g07154	24hr	208.9	700.3	-1.60	2.00E-05	592.5	607.7	-0.20	2.33E-01	Un-changed	
Os.13866.2.S1_at	6hr	358.5	871	-1.40	1.67E-04	399	1249.5	-1.40	8.90E-05	Down-regulated	expressed protein
LOC_Os03g55670	24hr	142.1	211.4	-0.80	2.25E-03	345	349	-0.10	5.00E-01	Un-changed	
Os.14076.1.S1_s_at	6hr	649.9	2131	-1.40	2.00E-05	834.8	2988.1	-1.70	2.00E-05	Down-regulated	cellular retinaldehyde-binding/triple function, C-terminal
LOC_Os02g44820	24hr	2205	2990.7	-0.50	2.00E-05	2386.4	2926.6	-0.40	5.20E-05	Un-changed	
Os.14139.1.S1_at	6hr	2531.7	5739.4	-1.10	2.00E-05	3021.6	7107.3	-1.10	2.00E-05	Down-regulated	AKIN gamma

Probe Set ID/Locus ID	Time	Quinclorac vs. Mock Repeat 1				Quinclorac vs. Mock Repeat 2				Assignment	Description
		Quinclorac	Mock	Log Ratio	P-value	Quinclorac	Mock	Log Ratio	P-value		
LOC_Os04g31340	24hr	2394.2	4425	-0.80	2.00E-05	3884.1	4604.1	-0.20	1.20E-02	Un-changed	
Os.14151.1.S1_at	6hr	965.4	1730.5	-1.00	2.70E-05	1012.7	2117.7	-1.10	2.00E-05	Down-regulated	expressed protein
LOC_Os01g49310	24hr	365.9	766.1	-0.90	2.00E-05	389	807.7	-0.70	4.00E-05	Un-changed	
Os.14181.1.S1_at	6hr	564.8	1814.9	-1.70	2.00E-05	608.8	1896.9	-1.70	2.00E-05	Down-regulated	dnaJ protein
LOC_Os02g54130	24hr	2615.1	3039.4	-0.20	6.56E-02	2945.2	2948.1	0.00	5.00E-01	Un-changed	
Os.14862.1.S1_a_at	6hr	805.9	2613.6	-1.70	2.19E-04	951	2637.1	-1.40	2.19E-04	Down-regulated	SHR5-receptor-like kinase
LOC_Os08g10320	24hr	1174.4	1100.5	0.20	6.04E-02	547.8	1136.2	-1.20	2.19E-04	Un-changed	
Os.14862.1.S1_s_at	6hr	973.7	2365.8	-1.30	2.00E-05	1122.3	3302.8	-1.40	2.00E-05	Down-regulated	SHR5-receptor-like kinase
LOC_Os08g10320	24hr	1317	1294.3	0.00	5.00E-01	663.5	1378.5	-1.20	2.00E-05	Un-changed	
Os.15191.1.S1_s_at	6hr	1106.5	2456.1	-1.60	2.00E-05	1211.1	2785.1	-1.50	2.00E-05	Down-regulated	SNF1-related protein kinase regulatory subunit beta-1
LOC_Os05g41220	24hr	1393.3	2520	-0.90	2.00E-05	2002.2	2661.7	-0.30	1.30E-04	Un-changed	
Os.15428.1.S1_at	6hr	3849.8	7372.1	-1.30	4.00E-05	4514.6	10423.6	-1.50	2.00E-05	Down-regulated	wound inducive gene
LOC_Os01g50622	24hr	78	62.4	-0.10	5.00E-01	75.8	136.4	-0.30	3.31E-01	Un-changed	
Os.15537.1.S1_at	6hr	163.1	526.7	-1.50	1.47E-04	143.2	470.4	-1.60	2.30E-05	Down-regulated	cytochrome P450 76C4
LOC_Os08g39660	24hr	491.5	776.4	-0.30	7.12E-03	321	712.9	-1.30	2.30E-05	Un-changed	
Os.15607.1.S1_at	6hr	741.5	1434.2	-1.10	2.00E-05	746.9	1639.5	-1.30	2.00E-05	Down-regulated	influenza virus NS1A binding protein isoform 3
LOC_Os04g31120	24hr	2373.7	3194.6	-0.40	5.20E-05	1973.5	3648.6	-0.70	2.00E-05	Un-changed	
Os.16218.1.S1_at	6hr	170.1	652.8	-1.40	8.90E-05	154.5	515.1	-1.40	6.00E-05	Down-regulated	OsMFT1 - Rice MFT-Like1 homogous to Arabidopsis Mother of FT and TFL1 gene
LOC_Os06g30370	24hr	143.5	808.5	-1.90	6.80E-05	290.9	568.2	-0.60	4.92E-04	Un-changed	
Os.16761.1.S1_at	6hr	422.2	1336.9	-1.10	2.00E-05	483.1	1203.4	-1.30	2.00E-05	Down-regulated	expressed protein
LOC_Os07g16364	24hr	1468.1	1209.8	0.50	2.41E-04	1852.1	1008.8	0.70	7.80E-05	Un-changed	
Os.170.3.S1_at	6hr	6664	13826.5	-1.10	2.00E-05	5669.4	13937.3	-1.10	2.30E-05	Down-regulated	ferredoxin-6, chloroplast precursor
LOC_Os01g64120	24hr	12333.9	16209.8	-0.40	1.14E-04	13885.1	14149.3	0.00	5.00E-01	Un-changed	
Os.17014.1.S1_at	6hr	635.1	1334	-1.20	1.14E-04	752.7	1857	-1.50	2.00E-05	Down-regulated	leucoanthocyanidin reductase
LOC_Os04g53850	24hr	516.1	1174.3	-1.20	2.00E-05	797.7	1244.2	-0.70	2.00E-05	Un-changed	
Os.17014.1.S1_s_at	6hr	323.4	676	-1.40	7.80E-05	406.5	1259.2	-1.70	1.01E-04	Down-regulated	leucoanthocyanidin reductase
LOC_Os04g53850	24hr	407.8	696.3	-0.90	8.65E-04	564.4	833	-0.70	3.46E-04	Un-changed	
Os.17144.1.A1_at	6hr	316.8	784.9	-1.30	2.00E-05	361.7	1250.9	-1.70	2.00E-05	Down-regulated	ATP binding protein
LOC_Os04g59000	24hr	372.8	756.8	-0.80	2.70E-05	425.1	1094.3	-1.30	2.00E-05	Un-changed	
Os.17190.1.A1_s_at	6hr	435.3	835.8	-1.00	2.00E-05	492.5	1092	-1.20	2.00E-05	Down-regulated	receptor-like protein kinase precursor
LOC_Os11g36150	24hr	412	536.1	-0.40	1.83E-03	353.6	669.6	-1.20	2.00E-05	Un-changed	
Os.17219.1.S1_at	6hr	413.9	1027.5	-1.30	2.00E-05	489	1557	-1.50	2.00E-05	Down-regulated	expressed protein
LOC_Os11g09680	24hr	705	1212.1	-0.80	6.00E-05	1045.9	1467.1	-0.50	6.80E-05	Un-changed	
Os.17219.2.S1_s_at	6hr	873.3	2041.8	-1.40	2.00E-05	794.8	2243.2	-1.60	2.00E-05	Down-regulated	expressed protein
LOC_Os11g09684	24hr	1257.7	2289.1	-0.80	2.00E-05	1678.6	2713.6	-0.60	2.00E-05	Un-changed	
Os.17356.1.A1_a_at	6hr	1354.5	4580.6	-1.80	2.00E-05	1543.1	5917.8	-1.90	2.00E-05	Down-regulated	benzoate carboxyl methyltransferase
LOC_Os11g15040	24hr	4669.1	5033.2	-0.10	5.00E-01	2457	5094.5	-1.00	2.00E-05	Un-changed	
Os.17405.1.S1_a_at	6hr	329.1	1104.9	-1.50	4.60E-05	466	1821.4	-1.90	3.50E-05	Down-regulated	ureide permease 4
LOC_Os12g31860	24hr	1082.1	2615.7	-1.30	2.30E-05	4093.9	2806.3	0.60	2.14E-04	Un-changed	
Os.17446.2.S1_at	6hr	276.3	521.5	-1.10	4.60E-05	255.5	626.7	-1.20	2.30E-05	Down-regulated	ATPP2-A13
LOC_Os04g48270	24hr	515.2	437.2	0.20	1.81E-02	349.9	366.7	0.10	5.00E-01	Un-changed	
Os.17474.1.A1_at	6hr	60.5	162.9	-1.70	2.00E-05	69.8	420.9	-2.90	3.00E-05	Down-regulated	protein kinase Kelch repeat:Kelch
LOC_Os02g11790	24hr	144.9	197.4	-0.20	9.29E-03	111.9	219.4	-1.20	1.47E-04	Un-changed	
Os.17655.1.S1_at	6hr	182.7	430.4	-1.30	6.00E-05	279.6	604.1	-1.20	2.30E-05	Down-regulated	OsIAA8 - Auxin-responsive Aux/IAA gene family member
LOC_Os02g49160	24hr	543.7	311.1	0.20	2.23E-01	396.2	492.7	-0.40	6.92E-04	Un-changed	
Os.17761.1.S1_a_at	6hr	792.3	2454.5	-1.50	2.00E-05	628.4	2184.9	-1.60	2.00E-05	Down-regulated	ACR4
LOC_Os04g32110	24hr	625.7	1219.2	-0.90	4.00E-05	440.3	846.1	-1.00	3.00E-05	Un-changed	
Os.17761.2.S1_x_at	6hr	586.6	1468.9	-1.30	2.00E-05	416.6	1508.9	-1.60	2.00E-05	Down-regulated	ACR4
LOC_Os04g32110	24hr	557.2	797.3	-0.70	2.30E-05	356.4	734.2	-0.70	2.30E-05	Un-changed	
Os.17824.1.S1_x_at	6hr	217.4	408.2	-1.10	8.90E-05	151.8	468	-1.00	2.00E-05	Down-regulated	ATP binding protein
LOC_Os02g42620	24hr	241.5	228.5	-0.10	3.31E-01	78.2	201.2	-1.30	7.12E-03	Un-changed	
Os.17918.1.S1_at	6hr	2164.8	5136.7	-1.20	2.00E-05	1475.3	4634.6	-1.60	2.00E-05	Down-regulated	serine/threonine kinase-like protein
LOC_Os03g16950	24hr	3007.8	4642.6	-0.60	2.00E-05	814.1	2321.7	-1.30	2.00E-05	Un-changed	

Probe Set ID/Locus ID	Time	Quinclorac vs. Mock Repeat 1				Quinclorac vs. Mock Repeat 2				Assignment	Description
		Quinclorac	Mock	Log Ratio	P-value	Quinclorac	Mock	Log Ratio	P-value		
Os.18397.1.S1_at	6hr	783.9	1897.3	-1.00	2.00E-05	1145.9	2241.6	-1.00	2.00E-05	Down-regulated	chaperone protein dnaJ
LOC_Os02g46640	24hr	1667.4	2483.9	-0.50	2.30E-05	2840.1	2753.3	0.00	5.00E-01	Un-changed	
Os.18633.1.S1_at	6hr	229.9	529.4	-1.30	7.80E-05	243.2	525.1	-1.20	6.80E-05	Down-regulated	calcium-dependent protein kinase
LOC_Os04g43710	24hr	520.2	578.1	-0.10	5.00E-01	572.8	470.9	0.20	2.85E-01	Un-changed	
Os.18829.1.A1_at	6hr	100.9	414.4	-2.00	2.00E-05	184.1	560.8	-1.70	2.00E-05	Down-regulated	hypothetical protein
LOC_Os11g20020	24hr	457.2	444.4	0.00	5.00E-01	418.7	484.3	0.00	5.00E-01	Un-changed	
Os.19105.1.S1_at	6hr	63.1	235.7	-1.50	2.41E-04	92.4	477.3	-2.70	8.65E-04	Down-regulated	influenza virus NS1A binding protein isoform 3
LOC_Os04g31120	24hr	491.4	524.6	0.20	1.95E-01	422.5	255.1	0.60	6.92E-04	Un-changed	
Os.20163.1.S1_at	6hr	320.4	886.9	-1.60	2.00E-05	301	975.1	-1.70	1.47E-04	Down-regulated	metal ion binding protein
LOC_Os03g02860	24hr	313	355.3	0.00	5.00E-01	218.6	259.7	-0.30	7.79E-03	Un-changed	
Os.20482.1.S1_at	6hr	592	1464.5	-1.60	2.00E-05	570.7	2256.9	-2.10	2.30E-05	Down-regulated	ATPP2-A13
LOC_Os03g02550	24hr	1685.9	1173.7	0.50	2.00E-05	1433.2	1508.5	0.00	5.00E-01	Un-changed	
Os.20861.1.S1_at	6hr	280.8	613.5	-1.10	2.70E-05	385	784	-1.10	2.00E-05	Down-regulated	receptor-like protein kinase precursor
LOC_Os11g36150	24hr	347.4	390.2	-0.20	1.42E-02	259.9	427.2	-0.70	2.00E-05	Un-changed	
Os.20936.1.S1_a_at	6hr	623.5	1945.9	-1.80	2.00E-05	807.2	2760.2	-2.10	2.00E-05	Down-regulated	Photosystem II 10kDa polypeptide, chloroplast precursor
LOC_Os07g05360	24hr	2577.8	4813.1	-0.90	2.00E-05	3239.8	4937.4	-0.60	2.00E-05	Un-changed	
Os.21282.1.S1_at	6hr	1585.7	3430.5	-1.10	4.60E-05	1263.2	2444.1	-1.00	5.20E-05	Down-regulated	zinc finger protein 7
LOC_Os03g13600	24hr	1337.6	2212.1	-0.90	6.80E-05	1530.1	1754.8	-0.50	8.90E-05	Un-changed	
Os.21349.1.S1_at	6hr	2098.8	4427.5	-1.10	2.00E-05	1929.3	4856	-1.30	2.00E-05	Down-regulated	expansin-like 3 precursor
LOC_Os03g04020	24hr	4494.6	5753.6	-0.40	1.65E-03	4008.7	5849.3	-0.50	2.00E-05	Un-changed	
Os.21803.1.S1_at	6hr	451	1300.6	-1.40	2.00E-05	709.4	1984.3	-1.40	2.00E-05	Down-regulated	expressed protein
LOC_Os06g24190	24hr	421.4	703.3	-0.90	1.14E-04	811.4	959.9	-0.10	5.00E-01	Un-changed	
Os.21893.3.A1_at	6hr	147.8	451.1	-1.50	1.67E-04	83.6	655.5	-2.20	3.50E-05	Down-regulated	trehalose 6-phosphate synthase
LOC_Os08g31980	24hr	83.5	328.7	-1.70	3.89E-04	227.2	336.9	-0.60	2.12E-02	Un-changed	
Os.22312.3.A1_a_at	6hr	10208.7	19895.8	-1.00	2.00E-05	12282.2	24696.9	-1.00	2.00E-05	Down-regulated	universal stress protein
LOC_Os05g28740	24hr	8085.8	13239.9	-0.80	2.00E-05	10062.9	13751.7	-0.60	2.00E-05	Un-changed	
Os.22415.1.S1_s_at	6hr	973.4	2381.2	-1.20	1.47E-04	1351.1	2836	-1.00	2.00E-05	Down-regulated	cytochrome P450 71D10
LOC_Os06g43430	24hr	1601.2	1548.7	-0.10	5.00E-01	1515.9	1977	-0.40	1.67E-04	Un-changed	
Os.22678.1.A1_s_at	6hr	854.1	1400.8	-1.20	2.00E-05	761.9	1932	-1.40	2.00E-05	Down-regulated	expressed protein
LOC_Os02g37834	24hr	1199	2382.9	-0.80	2.00E-05	1932.9	2804.8	-0.40	2.30E-05	Un-changed	
Os.22820.1.A1_at	6hr	234.6	413.6	-1.10	2.00E-05	204.7	646.6	-1.50	3.46E-04	Down-regulated	expressed protein
LOC_Os09g07150	24hr	260.3	504.1	-1.50	2.00E-05	490.5	716	-0.50	1.34E-03	Un-changed	
Os.22947.1.S1_a_at	6hr	1460.7	3142.7	-1.10	2.00E-05	1221.4	3404.5	-1.40	2.00E-05	Down-regulated	cDNA clone:J023037I04, full insert sequence.
	24hr	2854.5	4715	-0.70	2.00E-05	2530	3922.1	-0.70	2.30E-05	Un-changed	
Os.23067.1.S1_at	6hr	908.9	1887.3	-1.40	2.00E-05	829.9	2148	-1.30	2.00E-05	Down-regulated	expressed protein
LOC_Os04g55600	24hr	1858.6	3486.7	-0.80	2.00E-05	2135.2	2669.8	-0.40	4.92E-04	Un-changed	
Os.23145.1.S1_at	6hr	208	435	-1.10	2.00E-05	184.2	559.6	-1.50	1.47E-04	Down-regulated	RNA binding protein
LOC_Os08g03310	24hr	970.5	673.2	0.70	1.30E-04	1119.6	799.3	0.50	2.41E-04	Un-changed	
Os.23327.2.S1_a_at	6hr	542.5	4723.2	-3.20	2.00E-05	1001.3	6527.3	-2.80	2.00E-05	Down-regulated	OsWAK112d - OsWAK receptor-like protein kinase
LOC_Os10g10130	24hr	2604.1	3964.3	-0.50	2.00E-05	2080	4995.9	-1.20	2.00E-05	Un-changed	
Os.23635.1.S1_at	6hr	137.9	520.4	-1.70	2.00E-05	232	793	-1.80	2.00E-05	Down-regulated	expressed protein
LOC_Os01g62830	24hr	253.3	311.5	-0.30	3.58E-02	280.7	306.6	-0.10	5.00E-01	Un-changed	
Os.24100.1.A1_at	6hr	151.7	343.6	-1.40	1.34E-03	106.3	391.2	-1.60	8.65E-04	Down-regulated	F-box domain containing protein
LOC_Os07g13890	24hr	146.8	248	0.10	4.16E-01	152.7	189.5	-0.40	1.61E-01	Un-changed	
Os.24864.3.S1_at	6hr	610.9	1213.6	-1.00	2.70E-05	394.8	1116.6	-1.50	2.00E-05	Down-regulated	acid phosphatase
LOC_Os05g10310	24hr	345.5	750.4	-1.00	2.00E-05	865	834	0.00	5.00E-01	Un-changed	
Os.25117.1.A1_at	6hr	461.4	1271	-1.40	2.00E-05	554.1	1274.9	-1.40	2.00E-05	Down-regulated	expressed protein
LOC_Os01g07890	24hr	1160.7	1581.9	-0.40	1.30E-04	1093.3	1414.3	-0.20	2.67E-02	Un-changed	
Os.25589.1.S1_at	6hr	2028.6	4886	-1.20	2.00E-05	2248.5	6127.3	-1.50	2.00E-05	Down-regulated	octicosapeptide/Phox/Bem1p
LOC_Os11g25780	24hr	3987.7	6085.3	-0.70	2.00E-05	5461.6	7474.7	-0.40	1.34E-03	Un-changed	
Os.26494.1.S1_at	6hr	86.6	203.4	-1.40	1.20E-03	138.3	382.9	-1.20	1.20E-03	Down-regulated	SRC2
LOC_Os07g47400	24hr	115.2	144.6	0.20	5.00E-01	501.9	171.1	1.20	4.92E-04	Un-changed	
Os.26511.1.S1_at	6hr	477	1426.3	-1.40	2.00E-05	565.7	3072.2	-2.40	2.00E-05	Down-regulated	expressed protein

Probe Set ID/Locus ID	Time	Quinclorac vs. Mock Repeat 1				Quinclorac vs. Mock Repeat 2				Assignment	Description
		Quinclorac	Mock	Log Ratio	P-value	Quinclorac	Mock	Log Ratio	P-value		
LOC_Os04g49370	24hr	6011.6	3163.8	0.80	2.00E-05	2793	3538.5	-0.40	4.60E-05	Un-changed	
Os.26687.1.S1_at	6hr	3772.3	9064.9	-1.10	2.00E-05	1455.4	7203.3	-2.20	2.00E-05	Down-regulated	malate synthase, glyoxysomal
LOC_Os04g40990	24hr	7830.6	15838.9	-0.90	2.00E-05	4785.4	8783.9	-0.90	2.00E-05	Un-changed	
Os.26698.1.S1_a_at	6hr	325.4	1331.6	-1.90	2.00E-05	538.1	1977.9	-1.80	2.00E-05	Down-regulated	universal stress protein
LOC_Os01g19820	24hr	799.8	1202.7	-0.50	6.00E-05	967.9	1323.1	-0.30	6.92E-04	Un-changed	
Os.26698.4.S1_s_at	6hr	168.7	446.2	-1.10	2.30E-05	265.8	583.3	-1.30	5.20E-05	Down-regulated	universal stress protein
LOC_Os01g19820	24hr	375	499	-0.50	4.92E-04	367.6	571.9	-0.60	7.12E-03	Un-changed	
Os.26792.1.S1_at	6hr	434.4	1245.9	-1.50	2.00E-05	493.1	1345.6	-1.40	2.00E-05	Down-regulated	receptor-like protein kinase precursor
LOC_Os11g36160	24hr	463.9	956.2	-1.10	2.00E-05	608.3	1032.6	-0.80	2.00E-05	Un-changed	
Os.26816.1.A1_s_at	6hr	193.3	808.9	-2.10	2.00E-05	220.7	920.8	-1.80	2.00E-05	Down-regulated	protein kinase
LOC_Os07g35410	24hr	403.6	734.9	-0.80	2.00E-05	451.7	825.9	-0.70	3.50E-05	Un-changed	
Os.27039.1.S1_at	6hr	358.9	644.8	-1.10	2.00E-05	358.4	747.9	-1.00	2.73E-04	Down-regulated	OsWRKY30 - Superfamily of rice TFs having WRKY and
LOC_Os08g38990	24hr	412.3	412.1	0.20	1.18E-01	308.5	513.7	-0.70	1.01E-02	Un-changed	zinc finger domains
Os.27092.1.S1_at	6hr	226.8	448.4	-1.10	2.00E-05	167	502.1	-1.30	2.00E-05	Down-regulated	serine/threonine-protein kinase Nek4
LOC_Os07g08000	24hr	330.7	419.4	-0.70	6.92E-04	499.8	462	-0.10	5.00E-01	Un-changed	
Os.27185.1.S1_x_at	6hr	101.5	275.4	-1.50	5.20E-05	72.8	199.1	-1.60	1.89E-04	Down-regulated	CRK10
LOC_Os07g35750	24hr	176	216.3	-0.20	1.61E-01	103.1	167.3	-1.00	2.49E-03	Un-changed	
Os.27242.1.S1_at	6hr	583.5	1640.6	-1.40	2.00E-05	874.5	1951.9	-1.20	2.00E-05	Down-regulated	SHR5-receptor-like kinase
LOC_Os04g52614	24hr	827.8	1063.2	-0.40	2.73E-04	873.6	1019.5	-0.50	1.20E-03	Un-changed	
Os.27242.1.S1_x_at	6hr	489.9	1117.2	-1.40	2.00E-05	683.8	1609.7	-1.10	2.00E-05	Down-regulated	SHR5-receptor-like kinase
LOC_Os04g52614	24hr	625.7	835.9	-0.50	2.88E-02	690.2	1017.6	-0.40	2.49E-03	Un-changed	
Os.27290.1.A1_at	6hr	1782.9	3863.3	-1.20	2.00E-05	1349.4	3832	-1.60	2.00E-05	Down-regulated	alkaline alpha galactosidase 2
LOC_Os06g07600	24hr	2238.6	4146.5	-0.90	2.00E-05	2835.2	3570.3	-0.40	1.49E-03	Un-changed	
Os.27490.1.A1_at	6hr	127.4	655.3	-2.00	2.00E-05	157.7	1046.5	-2.50	2.00E-05	Down-regulated	expressed protein
LOC_Os01g29804	24hr	181.2	744.5	-1.80	2.00E-05	515.8	834.3	-0.50	3.46E-04	Un-changed	
Os.27497.1.S1_at	6hr	48.6	225.5	-2.00	2.00E-05	11.7	387	-4.70	2.30E-05	Down-regulated	transposon protein, Ac/Ds sub-class
LOC_Os12g12390	24hr	203.8	277.4	-0.60	1.47E-04	159.5	424.2	-1.50	2.00E-05	Un-changed	
Os.27506.1.A1_at	6hr	274.3	830.2	-1.60	2.00E-05	404.5	970.1	-1.30	3.50E-05	Down-regulated	benzoate carboxyl methyltransferase
LOC_Os11g15340	24hr	563.8	1723	-1.50	2.00E-05	955.3	1681.1	-0.80	2.00E-05	Un-changed	
Os.27638.1.S1_at	6hr	1391.6	7131.3	-2.30	2.00E-05	2380.6	10822.5	-2.20	2.00E-05	Down-regulated	glutamate synthase, chloroplast precursor
LOC_Os05g48200	24hr	54.2	157	-1.10	5.52E-04	145	275.2	-0.70	9.99E-02	Un-changed	
Os.27702.1.S1_x_at	6hr	256.6	421.3	-1.00	3.00E-05	264	572.8	-1.00	1.14E-04	Down-regulated	disease resistance protein RPS2
LOC_Os09g10054	24hr	499.5	331.1	0.10	2.63E-01	234.1	267.8	-0.60	1.83E-03	Un-changed	
Os.27712.2.S1_x_at	6hr	644.7	1506.9	-1.30	3.50E-05	765.6	1734.9	-1.20	2.00E-05	Down-regulated	choline monooxygenase, chloroplast precursor
LOC_Os06g48510	24hr	991.6	1363.9	-0.50	2.73E-04	1167.1	1576	-0.10	5.00E-01	Un-changed	
Os.27715.1.S1_a_at	6hr	311.2	1067.2	-1.80	2.00E-05	394.2	1334.1	-1.60	2.00E-05	Down-regulated	cytochrome P450 76C4
LOC_Os10g08474	24hr	645.7	1137.3	-0.90	2.00E-05	994.6	1339.3	-0.20	3.04E-03	Un-changed	
Os.27765.1.S1_at	6hr	1488.9	3357.3	-1.30	2.00E-05	1538	2957.3	-1.10	2.00E-05	Down-regulated	expressed protein
LOC_Os06g50080	24hr	960.5	769.7	0.20	5.00E-01	562	811.1	-0.30	1.46E-01	Un-changed	
Os.27780.2.S2_a_at	6hr	1249.5	2559.7	-1.10	2.00E-05	1543.5	3348.9	-1.10	2.00E-05	Down-regulated	expressed protein
LOC_Os10g36260	24hr	985.7	1458.6	-0.60	1.30E-04	1157.8	1859.1	-0.70	2.00E-05	Un-changed	
Os.27804.1.S1_at	6hr	1234	2635.4	-1.10	2.00E-05	1426	3399.6	-1.10	2.00E-05	Down-regulated	SHR5-receptor-like kinase
LOC_Os08g10310	24hr	2951.9	2380.5	0.30	3.46E-04	438.6	1808.7	-1.90	2.00E-05	Un-changed	
Os.27828.1.S1_a_at	6hr	196.7	483.8	-1.60	2.00E-05	201.5	463.9	-1.30	2.00E-05	Down-regulated	vacuolar cation/proton exchanger 1b
LOC_Os05g51610	24hr	211.3	297.6	-0.60	1.89E-04	179.9	137.9	0.10	5.00E-01	Un-changed	
Os.28301.1.S2_at	6hr	62.6	150.7	-1.40	3.46E-04	98.8	205	-1.10	7.80E-05	Down-regulated	photosystem II 10 kDa polypeptide, chloroplast
LOC_Os07g05360	24hr	514.1	349.7	0.60	1.30E-04	173.5	382.8	-1.10	2.00E-05	Un-changed	precursor
Os.28435.4.S1_at	6hr	125.8	394.7	-1.30	2.00E-05	313.6	621.5	-1.30	2.00E-05	Down-regulated	flavonol synthase/flavanone 3-hydroxylase
LOC_Os10g40934	24hr	183.4	186.9	0.10	2.23E-01	152.8	183.9	-0.50	1.46E-01	Un-changed	
Os.28435.4.S1_x_at	6hr	745	1447.5	-1.20	2.00E-05	946	2059	-1.30	2.00E-05	Down-regulated	flavonol synthase/flavanone 3-hydroxylase
LOC_Os10g40934	24hr	829.1	915.1	-0.10	5.00E-01	466.2	812.9	-0.60	1.20E-02	Un-changed	
Os.2938.1.S1_at	6hr	291.6	606.5	-1.00	2.00E-05	294.2	744.3	-1.10	4.48E-03	Down-regulated	expansin-like 3 precursor
LOC_Os10g39640	24hr	424.3	432.6	-0.60	9.29E-03	453.3	429.8	0.20	5.00E-01	Un-changed	

Probe Set ID/Locus ID	Time	Quinclorac vs. Mock Repeat 1				Quinclorac vs. Mock Repeat 2				Assignment	Description
		Quinclorac	Mock	Log Ratio	P-value	Quinclorac	Mock	Log Ratio	P-value		
Os.29966.1.S1_at	6hr	138.2	377.9	-1.10	5.20E-05	147	443.2	-1.50	5.93E-03	Down-regulated	apocytochrome F, C-terminal family protein
LOC_Os01g65910	24hr	162.3	382.2	-1.10	1.89E-04	218.7	315.7	-0.50	2.75E-03	Un-changed	
Os.30101.1.S1_at	6hr	429.8	818.7	-1.30	3.00E-05	417.4	1069	-1.20	2.00E-05	Down-regulated	expressed protein
LOC_Os06g12129	24hr	510.1	946	-0.70	2.30E-05	714.1	990.6	-0.50	1.01E-04	Un-changed	
Os.30993.1.S1_at	6hr	580.5	1892.6	-1.40	2.00E-05	731.5	1775.8	-1.20	2.30E-05	Down-regulated	expressed protein
LOC_Os07g12390	24hr	1083	1686.6	-0.40	3.00E-05	1401.7	1760.4	-0.30	7.12E-03	Un-changed	
Os.31088.1.S1_at	6hr	662.4	4529.2	-2.20	2.00E-05	821.3	4943.3	-2.40	2.00E-05	Down-regulated	OsGrx_C7.2 - glutaredoxin subgroup III
LOC_Os01g26912	24hr	2939.9	4439.2	-0.50	6.00E-05	3568.6	4846.1	-0.30	2.00E-05	Un-changed	
Os.32455.1.S1_at	6hr	373.1	1252.7	-1.50	2.30E-05	349	1123.5	-1.50	2.30E-05	Down-regulated	coiled-coil domain-containing protein 25
LOC_Os01g54670	24hr	1001	1007.9	0.10	5.00E-01	1053	1004	0.10	5.00E-01	Un-changed	
Os.33624.2.S1_at	6hr	133.4	332.3	-1.40	1.67E-04	140.2	349.1	-1.40	2.49E-03	Down-regulated	proteasome subunit beta type 1
LOC_Os09g32800	24hr	132.4	194.5	-0.70	1.42E-02	288.1	319.7	-0.30	5.40E-02	Un-changed	
Os.33834.1.S1_x_at	6hr	206.1	386.6	-1.20	3.50E-05	196	461.1	-1.10	2.70E-05	Down-regulated	expressed protein
LOC_Os09g04924	24hr	395.2	415.7	-0.20	4.94E-01	582.2	407.5	0.50	9.66E-04	Un-changed	
Os.3400.1.S1_s_at	6hr	530.5	1137.5	-1.00	2.00E-05	590.4	1080.1	-1.10	2.00E-05	Down-regulated	zinc finger protein CONSTANS-LIKE 11
LOC_Os02g49230	24hr	737.9	750.3	0.10	5.00E-01	807.2	805.2	0.00	5.00E-01	Un-changed	
Os.34466.1.S1_s_at	6hr	1550.1	5172.6	-1.50	2.00E-05	1829.4	6984.6	-2.00	2.00E-05	Down-regulated	expressed protein
LOC_Os01g07970	24hr	5235.4	4397.9	0.10	4.55E-01	5211	5330.2	-0.10	5.00E-01	Un-changed	
Os.35028.1.S1_at	6hr	413.5	842.1	-1.00	2.00E-05	367.7	770.3	-1.30	2.00E-05	Down-regulated	expressed protein
LOC_Os04g23040	24hr	470.9	620.3	-0.50	1.14E-04	631.7	697.2	-0.10	2.96E-01	Un-changed	
Os.35049.1.S1_a_at	6hr	306.2	743.1	-1.10	2.00E-05	251.3	496	-1.10	4.00E-05	Down-regulated	transcription factor HBP-1b
LOC_Os01g59350	24hr	786.3	1343.9	-0.70	2.00E-05	815.5	1117.1	-0.50	1.30E-04	Un-changed	
Os.35196.1.S1_at	6hr	669	1742.7	-1.20	2.30E-05	838.3	2060.8	-1.30	2.00E-05	Down-regulated	MCB2 protein
LOC_Os05g51160	24hr	2014.6	2234.8	0.20	1.83E-03	2036.8	2482.2	-0.20	1.42E-02	Un-changed	
Os.35583.1.S1_at	6hr	21.3	145.8	-2.30	6.92E-04	38.9	231.2	-3.00	1.14E-04	Down-regulated	expressed protein
LOC_Os01g48950	24hr	112.7	151.6	-0.60	3.04E-03	89.4	156.4	-0.40	3.04E-03	Un-changed	
Os.36350.1.A1_at	6hr	109.8	589.6	-2.00	2.00E-05	198.1	833.7	-2.20	2.00E-05	Down-regulated	expressed protein
LOC_Os04g09350	24hr	364.9	686.6	-1.10	3.50E-05	460.1	817.5	-0.60	1.14E-04	Un-changed	
Os.36969.1.S1_at	6hr	3377.2	7363	-1.10	2.00E-05	3773.1	8103.9	-1.10	2.00E-05	Down-regulated	expressed protein
LOC_Os04g02880	24hr	1596.6	2529.4	-0.60	2.00E-05	1009.6	2596.1	-1.30	2.00E-05	Un-changed	
Os.37718.1.S1_at	6hr	8105.3	21433.8	-1.50	2.30E-05	5852.4	28243.1	-2.40	2.00E-05	Down-regulated	polygalacturonase-1 non-catalytic beta subunit precursor
LOC_Os10g26940	24hr	14805.1	27369.7	-0.90	2.00E-05	13484	31370.5	-1.30	2.00E-05	Un-changed	
Os.37745.1.S1_at	6hr	761	1903.6	-1.20	2.00E-05	671.4	1938.6	-1.20	2.00E-05	Down-regulated	pathogen-related protein
LOC_Os01g53090	24hr	3475.9	2594.9	0.40	6.80E-05	807.4	2066.5	-1.30	2.00E-05	Un-changed	
Os.37834.1.S1_a_at	6hr	4815.1	11785.4	-1.20	2.00E-05	4056	11014.9	-1.40	6.80E-05	Down-regulated	stem-specific protein TSJT1
LOC_Os04g58280	24hr	8084.5	10401.2	-0.40	2.70E-05	7567.7	8697.9	-0.20	4.41E-02	Un-changed	
Os.37913.1.S1_at	6hr	576	899.2	-1.00	2.00E-05	615	986.6	-1.00	6.80E-05	Down-regulated	expressed protein
LOC_Os04g31870	24hr	1355.3	1338.2	-0.20	2.43E-01	1451.3	1527.4	-0.20	2.96E-01	Un-changed	
Os.38192.1.S1_at	6hr	424.9	1417	-1.70	2.00E-05	658.6	1643.5	-1.40	2.00E-05	Down-regulated	polygalacturonase-like protein
LOC_Os07g14160	24hr	290.5	784.6	-0.80	3.50E-05	432.3	820.4	-0.80	2.00E-05	Un-changed	
Os.38205.1.S1_at	6hr	342.2	910	-1.60	2.00E-05	351.8	736.9	-1.00	2.00E-05	Down-regulated	expressed protein
LOC_Os01g57710	24hr	707.3	912.3	-0.40	5.93E-03	607.9	725.6	-0.30	3.04E-03	Un-changed	
Os.38207.1.S1_x_at	6hr	217.1	499.5	-1.20	2.30E-05	308.9	728.9	-1.10	1.67E-04	Down-regulated	expressed protein
LOC_Os11g41034	24hr	361.4	447.1	-0.30	1.31E-01	241.4	288.5	-0.30	1.20E-02	Un-changed	
Os.38249.2.S1_at	6hr	1786.9	3852	-1.10	2.00E-05	2462.6	5093.3	-1.10	2.00E-05	Down-regulated	early flowering 3
LOC_Os01g38530	24hr	1480.4	1787.4	-0.30	3.58E-02	2629.6	2242.4	0.20	3.33E-02	Un-changed	
Os.38269.1.S1_x_at	6hr	95.1	230.7	-1.50	7.74E-04	69.2	496	-1.50	2.41E-04	Down-regulated	F-box domain containing protein
LOC_Os10g03850	24hr	154.7	399.3	-0.80	9.66E-04	218.5	387	-0.60	2.48E-02	Un-changed	
Os.38309.1.S1_at	6hr	376.9	1094.1	-1.60	2.00E-05	365.1	1593.4	-2.00	2.00E-05	Down-regulated	expressed protein
LOC_Os06g36390	24hr	795.8	866.6	-0.30	2.23E-01	405.6	1028.8	-1.30	2.70E-05	Un-changed	
Os.38346.1.S1_x_at	6hr	146.6	479.9	-1.50	2.00E-05	205.2	483.2	-1.30	4.00E-05	Down-regulated	cytochrome P450 71C4
LOC_Os01g36350	24hr	373.7	448.1	-0.40	7.43E-02	346.1	635.9	-0.50	3.89E-04	Un-changed	
Os.39066.1.S1_at	6hr	316.1	1050.8	-2.00	2.00E-05	440	1231.3	-2.10	2.00E-05	Down-regulated	lichenase-2 precursor

Probe Set ID/Locus ID	Time	Quinclorac vs. Mock Repeat 1				Quinclorac vs. Mock Repeat 2				Assignment	Description
		Quinclorac	Mock	Log Ratio	P-value	Quinclorac	Mock	Log Ratio	P-value		
LOC_Os05g31140	24hr	3429.3	3313.8	0.10	3.07E-01	1866.8	1602	0.20	5.06E-02	Un-changed	
Os.39066.2.S1_at	6hr	269.3	708.5	-1.60	2.00E-05	189.3	803.8	-2.20	2.00E-05	Down-regulated	lichenase-2 precursor
LOC_Os05g31140	24hr	2225.1	1865.9	0.20	1.10E-02	1132.8	599.7	0.90	2.00E-05	Un-changed	
Os.39087.1.S1_at	6hr	223.5	764.7	-1.70	4.00E-05	318.3	894.4	-1.20	1.49E-03	Down-regulated	pathogen-related protein
LOC_Os01g14550	24hr	181	403.2	-1.10	5.20E-05	269.9	354.8	-0.50	9.66E-04	Un-changed	
Os.39652.1.S1_at	6hr	644.9	2476.5	-2.10	2.00E-05	915.9	3405.1	-2.00	2.00E-05	Down-regulated	OsSAUR39 - Auxin-responsive SAUR gene family member
LOC_Os09g37330	24hr	2868.6	1867.2	0.70	2.00E-05	1309.6	1970.6	-0.50	1.01E-04	Un-changed	
Os.39993.1.S1_at	6hr	517.5	1086	-1.00	2.00E-05	510.9	1526.4	-1.40	2.00E-05	Down-regulated	expressed protein
LOC_Os03g60639	24hr	1351.1	1547.1	-0.20	3.84E-02	1747	1614.2	0.00	5.00E-01	Un-changed	
Os.40028.1.A1_at	6hr	179.7	533.2	-1.50	2.00E-05	251.2	850.4	-1.60	2.00E-05	Down-regulated	conserved hypothetical protein
LOC_Os02g27830	24hr	301.1	652.2	-1.10	7.80E-05	745.2	1182.9	-0.60	1.67E-04	Un-changed	
Os.40338.1.S1_at	6hr	469.4	1000.4	-1.10	2.00E-05	596.1	1689.5	-1.20	2.00E-05	Down-regulated	Transcribed sequence
	24hr	548.2	668.3	-0.10	5.00E-01	1429.8	1464.7	0.20	6.15E-02	Un-changed	
Os.408.1.S1_a_at	6hr	738.7	1976.5	-1.40	2.00E-05	891.6	2956.8	-1.60	2.00E-05	Down-regulated	MYB59
LOC_Os01g74410	24hr	1592.3	2248.8	-0.50	1.01E-04	1878	2921.7	-0.60	2.00E-05	Un-changed	
Os.41164.1.S1_at	6hr	183.8	585.9	-1.70	2.00E-05	138.7	548.9	-2.10	1.67E-04	Down-regulated	DNA binding protein
LOC_Os01g44390	24hr	368.4	487.3	-0.30	3.36E-03	277.7	583.4	-1.10	3.04E-03	Un-changed	
Os.43417.1.S1_at	6hr	147.3	418.4	-1.20	3.00E-05	144.7	375.5	-1.40	4.00E-05	Down-regulated	cytochrome P450 71A6
LOC_Os01g12760	24hr	233.4	486.2	-1.00	2.30E-05	147.3	360.6	-0.90	2.00E-05	Un-changed	
Os.43624.1.S1_at	6hr	79.8	146.9	-1.00	1.83E-03	96.8	182.3	-1.20	4.92E-04	Down-regulated	hypothetical protein
LOC_Os07g23494	24hr	154.2	149.4	-0.10	3.67E-01	201.4	159.5	0.10	5.00E-01	Un-changed	
Os.4453.1.S1_at	6hr	111.4	995.4	-3.10	2.00E-05	135	1062	-2.90	2.00E-05	Down-regulated	expressed protein
LOC_Os06g03520	24hr	82.3	76.8	0.00	3.07E-01	196.5	128	0.60	6.18E-04	Un-changed	
Os.46444.1.S1_at	6hr	670.2	2995.4	-2.40	2.00E-05	882.5	3456.1	-1.90	2.00E-05	Down-regulated	expressed protein
LOC_Os09g07154	24hr	1341.4	3757.2	-1.60	2.00E-05	2556.3	3922.9	-0.70	2.00E-05	Un-changed	
Os.46444.1.S1_x_at	6hr	518.1	1444.2	-1.90	6.80E-05	630.2	1640.7	-1.50	2.00E-05	Down-regulated	expressed protein
LOC_Os09g07154	24hr	746.9	1875.4	-1.70	2.00E-05	1335	2029.5	-0.70	2.30E-05	Un-changed	
Os.46941.1.S1_s_at	6hr	1077.2	3275.8	-1.60	2.00E-05	1115	3658.9	-1.50	2.00E-05	Down-regulated	expressed protein
LOC_Os06g48500	24hr	3093.4	3742.4	-0.30	2.25E-03	3144.5	3508.3	-0.10	5.00E-01	Un-changed	
Os.47694.1.S1_at	6hr	706.1	1576.9	-1.10	1.30E-04	829	1833	-1.10	4.60E-05	Down-regulated	transposon protein, Mutator sub-class
LOC_Os03g10880	24hr	2060.3	2076.4	-0.10	5.00E-01	1679	2153.7	-0.20	5.00E-01	Un-changed	
Os.49077.1.A1_at	6hr	239.6	554.4	-1.10	1.67E-04	284.2	488.9	-1.00	1.01E-04	Down-regulated	glucan endo-1,3-beta-glucosidase 5 precursor
LOC_Os03g45390	24hr	481.4	547.2	-0.20	1.18E-01	928.9	634.9	0.40	9.29E-03	Un-changed	
Os.49079.1.A1_at	6hr	136.9	268.1	-1.10	4.60E-05	128.6	503.8	-1.70	4.60E-05	Down-regulated	PDI-like protein
LOC_Os04g51920	24hr	246.1	303.4	-0.20	4.55E-01	401.1	428.7	0.10	5.00E-01	Un-changed	
Os.49137.1.S1_at	6hr	70	253.6	-1.10	2.30E-05	193.6	495.5	-1.50	1.01E-04	Down-regulated	expressed protein
LOC_Os03g12879	24hr	359.8	440.2	-0.10	5.00E-01	571.8	691.9	-0.10	4.55E-01	Un-changed	
Os.49159.1.A1_x_at	6hr	923.2	1747.1	-1.30	2.00E-05	895.4	1814.8	-1.10	2.00E-05	Down-regulated	expressed protein
LOC_Os05g32970	24hr	2667.8	3500.6	-0.50	4.00E-05	1868	3390.6	-0.80	2.00E-05	Un-changed	
Os.49225.1.S1_at	6hr	138	429.5	-1.70	1.67E-04	178.5	666.7	-2.40	2.00E-05	Down-regulated	resistance protein
LOC_Os08g42700	24hr	1399.2	935.5	0.50	2.00E-05	442.8	518.9	-0.50	3.70E-03	Un-changed	
Os.49225.1.S2_at	6hr	196.8	1179.1	-2.60	2.70E-05	328.3	2170.3	-2.90	2.00E-05	Down-regulated	resistance protein
LOC_Os08g42700	24hr	3949.3	3125.1	0.40	1.83E-03	1054.3	3055.8	-1.50	2.00E-05	Un-changed	
Os.49328.1.S1_at	6hr	130.6	436.1	-1.40	1.89E-04	106.7	557.1	-1.40	1.47E-04	Down-regulated	peptidase/ subtilase
LOC_Os04g03796	24hr	310.6	343.2	-0.50	5.40E-02	4	212.6	-4.30	1.20E-03	Un-changed	
Os.49365.1.S1_at	6hr	982.2	1886.9	-1.00	2.00E-05	956.4	2196.8	-1.20	2.00E-05	Down-regulated	transposon protein, CACTA, En/Spm sub-class
LOC_Os01g56270	24hr	1927.4	1801.1	0.20	2.67E-02	2302	1953	0.20	8.51E-03	Un-changed	
Os.49529.1.S1_at	6hr	1267.6	2557.1	-1.00	2.00E-05	1811	4326.3	-1.20	2.00E-05	Down-regulated	expressed protein
LOC_Os06g43810	24hr	1848.2	1589.1	0.10	5.00E-01	1102.1	2031.2	-1.00	2.00E-05	Un-changed	
Os.49566.1.S1_at	6hr	88.9	196.1	-1.20	4.60E-05	124.6	288.6	-1.20	7.74E-04	Down-regulated	cytochrome P450 89A2
LOC_Os06g22340	24hr	111.6	220.6	-0.70	3.33E-02	145.4	176.6	-0.40	3.54E-01	Un-changed	
Os.49582.1.S1_at	6hr	2067.1	7347.6	-1.70	2.00E-05	3385.9	11175.8	-1.70	2.00E-05	Down-regulated	expressed protein
LOC_Os02g45930	24hr	11127.5	12064.2	-0.10	5.00E-01	9578.8	13037	-0.40	2.41E-04	Un-changed	

Probe Set ID/Locus ID	Time	Quinclorac vs. Mock Repeat 1				Quinclorac vs. Mock Repeat 2				Assignment	Description
		Quinclorac	Mock	Log Ratio	P-value	Quinclorac	Mock	Log Ratio	P-value		
Os.49676.1.S1_at	6hr	846.9	1532.8	-1.00	2.00E-05	896.7	2162.3	-1.20	2.00E-05	Down-regulated	expressed protein
LOC_Os04g55610	24hr	1170.4	2452.9	-1.10	2.70E-05	1217	1950.1	-0.70	4.60E-05	Un-changed	
Os.50234.1.S1_at	6hr	349.4	691.7	-1.10	1.14E-04	347.8	834.6	-1.20	2.00E-05	Down-regulated	expressed protein
LOC_Os06g47930	24hr	231.6	196.3	0.10	2.13E-01	135.8	221.9	-0.40	4.41E-02	Un-changed	
Os.50246.2.S1_x_at	6hr	1132	3285	-1.60	2.00E-05	1226.7	3119.7	-1.40	2.00E-05	Down-regulated	zinc finger protein CONSTANS-LIKE 16
LOC_Os02g49880	24hr	704.9	680.9	-0.30	1.54E-02	700.1	880.1	-0.70	2.14E-04	Un-changed	
Os.50366.1.S1_x_at	6hr	437.2	864.4	-1.20	7.80E-05	660.5	1455.4	-1.30	6.80E-05	Down-regulated	expressed protein
LOC_Os10g26150	24hr	389.9	478.3	-0.20	5.00E-01	501.4	712.2	-0.50	2.00E-05	Un-changed	
Os.50457.2.S1_at	6hr	155.4	467.1	-1.50	4.92E-04	246.5	466.6	-1.40	2.30E-05	Down-regulated	expressed protein
LOC_Os02g13304	24hr	211.8	482.8	-1.20	5.20E-05	342.9	533.6	-0.30	6.15E-02	Un-changed	
Os.50548.1.S1_at	6hr	372.1	698.1	-1.00	4.60E-05	465.3	1000.4	-1.10	2.30E-05	Down-regulated	protein binding protein
LOC_Os09g17610	24hr	604.1	593.3	-0.60	2.30E-05	750.1	910.8	-0.20	3.91E-01	Un-changed	
Os.50548.2.S1_x_at	6hr	243.3	382.2	-1.30	4.38E-04	253.4	617.8	-1.30	7.80E-05	Down-regulated	protein binding protein
LOC_Os09g17610	24hr	227	279.7	-0.40	4.92E-03	363.2	499	-0.10	5.00E-01	Un-changed	
Os.50801.1.S1_at	6hr	216	533.5	-1.20	4.00E-05	341.3	664.4	-1.10	7.80E-05	Down-regulated	expressed protein
LOC_Os07g17560	24hr	403.8	864.1	-0.90	6.00E-05	406.9	952.1	-0.90	7.80E-05	Un-changed	
Os.50889.1.S1_at	6hr	202.1	723.1	-1.70	3.00E-05	199.3	1014.6	-1.80	2.00E-05	Down-regulated	hypothetical protein
LOC_Os08g38150	24hr	269.2	802.9	-1.70	2.00E-05	947.2	979.2	-0.30	1.67E-02	Un-changed	
Os.51029.1.S1_at	6hr	848.5	2817.5	-1.70	2.00E-05	1355.7	4838.7	-1.80	3.50E-05	Down-regulated	PE-PGRS family protein
LOC_Os09g02770	24hr	2466.3	3937.9	-0.70	2.30E-05	3196.6	5138.5	-0.50	2.00E-05	Un-changed	
Os.51364.1.A1_at	6hr	297.3	470.7	-1.00	3.46E-04	131.6	353.8	-1.40	2.00E-05	Down-regulated	expressed protein
LOC_Os08g36580	24hr	251.3	487	-0.80	3.07E-04	209.4	401.3	-0.60	6.56E-02	Un-changed	
Os.51601.1.S1_at	6hr	189.5	431.7	-1.20	3.00E-05	196.3	654.8	-1.60	1.01E-04	Down-regulated	zinc finger protein 7
LOC_Os07g40300	24hr	501.1	256.6	0.80	2.00E-05	344.8	434	-0.40	2.85E-01	Un-changed	
Os.51637.1.S1_x_at	6hr	270.2	1008.7	-2.00	2.00E-05	272.2	1757.5	-2.50	2.00E-05	Down-regulated	expressed protein
LOC_Os01g29804	24hr	292.1	1148.3	-2.20	2.00E-05	1037.2	1459.6	-0.50	2.30E-05	Un-changed	
Os.51883.1.S1_at	6hr	227	559.7	-1.30	2.00E-05	275.7	678.3	-1.40	2.00E-05	Down-regulated	glucan endo-1,3-beta-glucosidase 5 precursor
LOC_Os03g45390	24hr	702.8	541.6	0.50	2.25E-03	796.5	406.2	0.60	2.30E-05	Un-changed	
Os.52163.1.S1_at	6hr	4.4	217.3	-4.00	2.00E-05	116.3	246.2	-1.00	1.89E-04	Down-regulated	ATP binding protein
LOC_Os06g35850	24hr	102.1	110.5	0.40	2.74E-01	121	158.9	-0.70	3.67E-01	Un-changed	
Os.52185.1.A1_at	6hr	617.6	1471.7	-1.30	2.00E-05	877.4	1783.1	-1.20	2.00E-05	Down-regulated	expressed protein
LOC_Os04g35790	24hr	519.6	621.4	-0.40	7.12E-03	649	706.1	0.10	5.00E-01	Un-changed	
Os.52240.1.S1_at	6hr	1378.8	3887.4	-1.60	3.00E-05	1593.3	4202.6	-1.60	2.00E-05	Down-regulated	resistance protein CAN_RGA1
LOC_Os11g39190	24hr	2682	3098.5	-0.20	1.12E-01	2609.7	2998.7	-0.20	4.16E-01	Un-changed	
Os.52627.1.S1_at	6hr	132.9	365.8	-1.30	2.00E-05	142.2	372.4	-1.40	3.46E-04	Down-regulated	expressed protein
LOC_Os08g26230	24hr	85.2	114.6	-0.30	5.00E-01	14	34.7	-1.30	4.94E-01	Un-changed	
Os.52655.2.S1_at	6hr	167.3	355.7	-1.20	1.67E-04	178.5	296.9	-1.20	1.65E-03	Down-regulated	expressed protein
LOC_Os02g13020	24hr	241.3	353.1	-0.40	5.52E-04	366.7	340.7	-0.10	4.41E-02	Un-changed	
Os.5271.1.S1_at	6hr	128.2	323.9	-1.40	6.00E-05	137.6	315.2	-1.20	2.30E-05	Down-regulated	amidase
LOC_Os04g10530	24hr	63	118.6	-0.60	4.41E-02	123.8	64	0.70	4.81E-01	Un-changed	
Os.53014.1.S1_at	6hr	1133.3	2528.6	-1.00	2.00E-05	1128	3002.8	-1.30	2.00E-05	Down-regulated	polcalcin Jun o 2
LOC_Os06g47640	24hr	1948	3599.5	-0.70	2.00E-05	2046.6	3089.6	-0.50	4.60E-05	Un-changed	
Os.53103.1.S1_at	6hr	1577.4	3201	-1.00	2.00E-05	1068.6	2795.5	-1.40	2.00E-05	Down-regulated	expressed protein
LOC_Os05g32970	24hr	4799.1	7830.1	-0.60	2.00E-05	3198.4	5924.7	-0.80	2.00E-05	Un-changed	
Os.53103.1.S1_x_at	6hr	1362.9	3184.5	-1.10	2.00E-05	988.7	3103.4	-1.50	2.00E-05	Down-regulated	expressed protein
LOC_Os05g32970	24hr	4589.4	6775.1	-0.50	2.00E-05	3408.7	5409.1	-0.70	2.00E-05	Un-changed	
Os.54340.1.S1_at	6hr	713.8	1749.9	-1.70	2.00E-05	888.7	1672.6	-1.40	2.00E-05	Down-regulated	hypothetical protein
LOC_Os11g31250	24hr	897.9	1723.1	-0.90	2.00E-05	1166	1618.8	-0.40	4.92E-03	Un-changed	
Os.54340.1.S1_x_at	6hr	405.4	1302.9	-1.60	2.70E-05	523.1	1380.6	-1.40	2.00E-05	Down-regulated	hypothetical protein
LOC_Os11g31250	24hr	704	1388.9	-0.80	2.00E-05	999	1377.6	-0.30	3.70E-03	Un-changed	
Os.54443.1.S1_at	6hr	889.4	1696.4	-1.00	2.30E-05	557	1548	-1.20	2.00E-05	Down-regulated	ring-H2 zinc finger protein
LOC_Os07g42610	24hr	2209.3	2375.1	-0.20	5.00E-01	1063.1	1739.7	-0.50	6.50E-03	Un-changed	
Os.54859.1.S1_at	6hr	125.1	241.8	-1.10	2.30E-05	83	437	-1.40	3.50E-05	Down-regulated	RING-H2 finger protein ATL2A

Probe Set ID/Locus ID	Time	Quinclorac vs. Mock Repeat 1				Quinclorac vs. Mock Repeat 2				Assignment	Description
		Quinclorac	Mock	Log Ratio	P-value	Quinclorac	Mock	Log Ratio	P-value		
LOC_Os04g49550	24hr	246.5	262.4	-0.10	5.00E-01	240.2	279.4	-0.30	1.81E-02	Un-changed	
Os.55270.1.S1_s_at	6hr	1877.8	7238.4	-1.50	2.00E-05	1724.3	7565.2	-2.10	2.00E-05	Down-regulated	expressed protein
LOC_Os04g54210	24hr	7374.6	5412.3	0.40	3.00E-05	7198	5468.6	0.30	6.18E-04	Un-changed	
Os.55386.1.A1_at	6hr	235.4	789	-1.50	6.80E-05	394.3	1601.6	-2.10	5.20E-05	Down-regulated	ethylene-responsive transcription factor 5
LOC_Os10g41330	24hr	1546.3	598.2	1.30	2.70E-05	410.5	447.6	0.00	4.16E-01	Un-changed	
Os.55402.1.S1_at	6hr	337.7	2206.2	-2.90	2.00E-05	221.6	2216.9	-3.10	2.00E-05	Down-regulated	OsGrx_C17 - glutaredoxin subgroup III
LOC_Os11g43520	24hr	821.3	934.7	-0.10	5.00E-01	1098.1	1044.3	0.20	2.85E-01	Un-changed	
Os.55511.1.S1_at	6hr	105	313.4	-1.70	4.38E-04	86.3	345.5	-1.90	3.50E-05	Down-regulated	integral membrane protein
LOC_Os04g45520	24hr	47.3	33.9	0.20	4.42E-01	64.1	19.8	1.80	7.89E-02	Un-changed	
Os.55524.1.S1_at	6hr	282.9	621	-1.30	2.00E-05	271.8	599.3	-1.30	2.00E-05	Down-regulated	RING-H2 finger protein ATL3C
LOC_Os05g36310	24hr	214.9	497	-1.10	5.20E-05	571.3	467.2	0.40	1.20E-03	Un-changed	
Os.55612.1.S1_at	6hr	2036.3	5524.4	-1.40	2.00E-05	2140.2	6564.9	-1.50	2.70E-05	Down-regulated	cyclase/dehydrase family protein
LOC_Os03g18600	24hr	6295.6	9099.5	-0.60	6.80E-05	4701.6	9034.2	-0.70	2.70E-05	Un-changed	
Os.55696.1.S1_at	6hr	428	797.6	-1.00	1.30E-04	353	848.3	-1.10	3.00E-05	Down-regulated	expressed protein
LOC_Os12g39100	24hr	123	103.4	0.30	2.96E-01	97.7	79.6	0.20	3.07E-01	Un-changed	
Os.5583.1.S1_at	6hr	166.5	594.3	-1.70	3.00E-05	229.5	765	-1.70	2.00E-05	Down-regulated	ER6 protein
LOC_Os03g19270	24hr	695.4	759.3	-0.10	2.96E-01	1218.8	977.5	0.40	1.34E-03	Un-changed	
Os.56029.1.S1_at	6hr	234.4	506.2	-1.50	3.50E-05	273.1	655.7	-1.10	2.30E-05	Down-regulated	C1-like domain containing protein
LOC_Os12g40470	24hr	284.6	282.1	0.10	5.00E-01	449.5	378.1	0.20	5.00E-01	Un-changed	
Os.56154.1.S1_s_at	6hr	201.1	299.3	-1.00	1.47E-04	90.3	181.8	-1.30	8.90E-05	Down-regulated	expressed protein
LOC_Os09g07150	24hr	119.6	371.9	-1.80	1.01E-04	172.5	246.7	-0.40	2.49E-03	Un-changed	
Os.6125.1.S1_at	6hr	1128.4	2689.9	-1.30	2.00E-05	1777.7	3420.7	-1.10	2.00E-05	Down-regulated	PDI-like protein
LOC_Os03g29190	24hr	2856.4	4550.2	-0.60	2.00E-05	4596.9	5430.2	-0.20	5.00E-01	Un-changed	
Os.6177.1.S2_a_at	6hr	97.4	364.8	-1.70	2.00E-05	146.4	622.3	-1.70	2.00E-05	Down-regulated	expressed protein
LOC_Os10g14180	24hr	195.1	459.5	-1.10	2.00E-05	442.3	521.9	-0.10	5.00E-01	Un-changed	
Os.6325.1.A1_x_at	6hr	255.4	606.5	-1.20	2.00E-05	352.1	764.4	-1.10	2.00E-05	Down-regulated	calcineurin B-like protein 10
LOC_Os01g39770	24hr	493.8	511.9	-0.10	4.29E-01	486.9	643.8	-0.40	2.70E-05	Un-changed	
Os.6375.1.S1_s_at	6hr	2123.7	3983.9	-1.00	2.00E-05	2319.5	4815.1	-1.00	2.00E-05	Down-regulated	catalytic/ hydrolase
LOC_Os01g63990	24hr	4613.2	5176.4	-0.10	5.00E-01	4009.2	5753.8	-0.50	2.00E-05	Un-changed	
Os.6375.2.S1_x_at	6hr	1762.4	4518	-1.00	2.00E-05	2318.7	4675.3	-1.00	2.00E-05	Down-regulated	catalytic/ hydrolase
LOC_Os01g63990	24hr	4896.3	4628.5	-0.10	5.00E-01	3475.2	4428	-0.50	2.00E-05	Un-changed	
Os.6542.1.S1_at	6hr	594.6	1166	-1.10	3.46E-04	558.7	1335.8	-1.40	2.00E-05	Down-regulated	dihydroflavonol-4-reductase
LOC_Os03g08624	24hr	1134.5	2035	-0.90	2.00E-05	1139.2	1913.9	-0.90	2.00E-05	Un-changed	
Os.6764.1.S1_at	6hr	134.3	852.5	-2.50	2.00E-05	109.1	1052.8	-2.90	2.00E-05	Down-regulated	conserved hypothetical protein
LOC_Os04g54310	24hr	1942.4	1207.8	0.60	3.50E-05	935.7	1332.8	-0.40	2.70E-05	Un-changed	
Os.7028.1.S1_at	6hr	715.5	1207.2	-1.00	2.00E-05	622.1	1719.4	-1.50	2.00E-05	Down-regulated	RING zinc finger protein-like
LOC_Os02g45710	24hr	3289.5	2082.7	0.40	2.70E-05	1018.1	1096.6	0.10	5.00E-01	Un-changed	
Os.7339.1.S1_at	6hr	1830.5	3772.5	-1.00	2.00E-05	1809.4	4407.1	-1.20	2.00E-05	Down-regulated	RING finger and CHY zinc finger domain-containing protein 1
LOC_Os10g31850	24hr	1426.5	2646.1	-0.90	2.00E-05	2494.7	3103.5	-0.30	2.00E-05	Un-changed	
Os.7612.1.S1_at	6hr	2601.9	7579.2	-1.40	2.00E-05	4449.8	11372.5	-1.20	2.00E-05	Down-regulated	Bowman-Birk type trypsin inhibitor
LOC_Os03g60840	24hr	7497.9	11166.2	-0.40	3.00E-05	11300.5	12972.6	-0.20	6.50E-03	Un-changed	
Os.7705.1.S1_at	6hr	394.7	1200.4	-1.60	2.00E-05	570.4	1502.1	-1.60	2.00E-05	Down-regulated	wound induced protein
LOC_Os04g54300	24hr	3829.3	1873.6	1.00	2.00E-05	1790.9	1773.3	0.00	5.00E-01	Un-changed	
Os.7935.1.S1_at	6hr	3511.5	10570.5	-1.60	2.00E-05	4256.9	15815.1	-1.90	2.00E-05	Down-regulated	sex determination protein tasselseed-2
LOC_Os07g46852	24hr	4744.9	11989.2	-1.30	2.00E-05	7343.5	12883.2	-0.70	2.00E-05	Un-changed	
Os.7935.1.S1_x_at	6hr	3171.5	7953.9	-1.60	2.00E-05	3500.3	11827.2	-2.00	2.00E-05	Down-regulated	sex determination protein tasselseed-2
LOC_Os07g46852	24hr	3444.3	9725.7	-1.30	2.00E-05	6177.2	9873.9	-0.70	2.00E-05	Un-changed	
Os.7935.2.S1_x_at	6hr	879.4	2562.3	-1.50	2.70E-05	769.7	3992.5	-2.00	2.00E-05	Down-regulated	sex determination protein tasselseed-2
LOC_Os07g46852	24hr	1151.2	3122.1	-1.40	2.00E-05	1467.7	2666.5	-0.80	6.00E-05	Un-changed	
Os.7947.1.S1_a_at	6hr	7973	18456.8	-1.20	2.00E-05	8337.1	20539.9	-1.30	2.00E-05	Down-regulated	lichenase-2 precursor
LOC_Os05g31140	24hr	22138.7	32612.3	-0.60	2.00E-05	28071.8	37723.8	-0.40	2.00E-05	Un-changed	
Os.8188.1.S1_s_at	6hr	310.3	865.5	-1.10	2.30E-05	550.6	1076.2	-1.10	2.00E-05	Down-regulated	pre-mRNA-splicing factor SF2
LOC_Os07g47630	24hr	607	818.1	-0.40	1.20E-03	659.6	1069	-0.50	6.80E-05	Un-changed	

Probe Set ID/Locus ID	Time	Quinclorac vs. Mock Repeat 1				Quinclorac vs. Mock Repeat 2				Assignment	Description
		Quinclorac	Mock	Log Ratio	P-value	Quinclorac	Mock	Log Ratio	P-value		
Os.8263.1.S1_s_at	6hr	725.3	2203.3	-1.20	2.00E-05	943.2	1911.9	-1.00	2.00E-05	Down-regulated	ATFP3
LOC_Os04g32030	24hr	2197.9	2329	0.00	5.00E-01	2331.5	2592.5	-0.10	5.00E-01	Un-changed	
Os.8493.1.A1_at	6hr	492.8	1480.9	-1.20	2.00E-05	613.1	1252.9	-1.10	2.00E-05	Down-regulated	cytochrome P450 71D10
LOC_Os06g43430	24hr	848.3	921.9	0.00	5.00E-01	698.6	1042.4	-0.40	2.41E-04	Un-changed	
Os.8507.1.S1_at	6hr	360.4	588.1	-1.20	2.00E-05	278.8	549.8	-1.20	2.00E-05	Down-regulated	UDP-glucose 4-epimerase
LOC_Os09g35800	24hr	454.2	559.9	-0.20	1.81E-02	475.1	706.7	-0.30	9.99E-02	Un-changed	
Os.8700.1.S1_at	6hr	691.7	2309.3	-1.80	2.00E-05	625.5	2292.3	-2.00	2.00E-05	Down-regulated	dynein light chain LC6, flagellar outer arm
LOC_Os02g16909	24hr	228	873.9	-1.70	2.00E-05	365.7	687.3	-0.60	2.00E-05	Un-changed	
Os.8735.1.S1_at	6hr	1880	5313	-1.50	1.14E-04	2448.1	5276.7	-1.00	3.07E-04	Down-regulated	trehalose synthase
LOC_Os05g44100	24hr	4405.7	4527.8	0.00	5.00E-01	4485	5109.8	-0.10	5.00E-01	Un-changed	
Os.8940.1.S1_at	6hr	311.4	617.7	-1.10	7.80E-05	371.9	855.8	-1.30	4.00E-05	Down-regulated	electron transporter
LOC_Os06g12030	24hr	300.8	287.4	-0.20	5.00E-01	305.5	290.8	0.00	5.00E-01	Un-changed	
Os.9556.1.S1_at	6hr	405.5	972.4	-1.30	2.00E-05	435.5	912.9	-1.20	2.00E-05	Down-regulated	nitrate and chloride transporter
LOC_Os12g44060	24hr	555.3	740.2	-0.70	2.30E-05	453.5	540.4	-0.20	2.04E-01	Un-changed	
Os.9580.1.S1_at	6hr	206.7	461.8	-1.00	8.65E-04	150.9	618.5	-1.30	6.80E-05	Down-regulated	cDNA clone:002-104-C04, full insert sequence.
	24hr	288.5	458.9	-0.60	4.92E-04	355.6	435.1	-0.30	1.67E-02	Un-changed	
OsAffx.12016.1.S1_at	6hr	304.5	945	-1.50	2.70E-05	307.5	972.9	-1.50	4.00E-05	Down-regulated	nudix hydrolase 8
LOC_Os02g13350	24hr	643.3	1175.3	-0.90	1.14E-04	839.5	1096.2	-0.40	3.50E-05	Un-changed	
OsAffx.13147.1.S1_s_at	6hr	54.5	308.8	-2.00	2.00E-05	19.7	464.5	-4.30	2.00E-05	Down-regulated	conserved hypothetical protein
LOC_Os03g31780	24hr	56.9	118.3	-1.20	7.80E-05	94.7	124.4	-0.60	7.43E-02	Un-changed	
OsAffx.13994.1.S1_at	6hr	59.9	265.1	-2.20	2.00E-05	44.2	376.5	-2.60	2.00E-05	Down-regulated	terpene synthase 7
LOC_Os04g27430	24hr	12.4	565	-5.60	2.00E-05	783.9	729.4	0.10	5.00E-01	Un-changed	
OsAffx.14410.1.S1_s_at	6hr	2.6	73.2	-4.80	1.14E-04	21.4	112.4	-2.60	1.47E-04	Down-regulated	wound-induced protein
LOC_Os04g54230	24hr	1224.7	627.1	0.80	5.20E-05	605.1	528.5	0.40	1.49E-03	Un-changed	
OsAffx.15371.1.S1_at	6hr	105.5	249.9	-1.20	7.80E-05	135.9	325.5	-1.20	2.49E-03	Down-regulated	LMBR1-like conserved region family protein
LOC_Os06g11520	24hr	177.2	204.5	-0.20	5.00E-01	60.8	149.8	-1.40	3.10E-02	Un-changed	
OsAffx.17384.1.S1_s_at	6hr	320.6	710.6	-1.10	6.92E-04	422.9	1334.5	-1.20	1.47E-04	Down-regulated	zinc finger, C3HC4 type family protein
LOC_Os08g37760	24hr	400.5	436.1	-0.40	4.07E-03	441.8	493.7	-0.40	4.60E-05	Un-changed	
OsAffx.20014.1.S1_at	6hr	216.4	796.6	-1.90	2.00E-05	227.9	776.1	-1.80	2.00E-05	Down-regulated	nucleotide pyrophosphatase/phosphodiesterase
LOC_Os12g38770	24hr	702.8	1211.6	-0.60	5.20E-05	237.6	1264.7	-2.20	2.00E-05	Un-changed	
OsAffx.27364.1.S1_at	6hr	102.1	460.7	-2.20	5.20E-05	86.8	518.2	-2.40	2.30E-05	Down-regulated	glutamate synthase, chloroplast precursor
LOC_Os01g48960	24hr	30.4	34.7	-0.10	5.00E-01	29.4	28.2	0.20	5.00E-01	Un-changed	
OsAffx.28454.1.S1_s_at	6hr	300.9	1241.8	-1.80	2.00E-05	288.8	1256.2	-1.90	2.00E-05	Down-regulated	expressed protein
LOC_Os07g15130	24hr	836.1	1257.2	-0.50	5.20E-05	950	1350.8	-0.50	4.00E-05	Un-changed	
OsAffx.29892.1.S1_at	6hr	336.3	823.7	-1.30	2.00E-05	511.5	1111.5	-1.10	2.30E-05	Down-regulated	expressed protein
LOC_Os08g36060	24hr	142	333.1	-1.30	1.67E-04	209.4	362.8	-0.90	4.92E-04	Un-changed	
OsAffx.30538.1.S1_x_at	6hr	288	682.1	-1.10	2.00E-05	491.6	1260.9	-1.40	2.00E-05	Down-regulated	expressed protein
LOC_Os10g26150	24hr	293.6	264.2	0.00	5.00E-01	377.4	484.1	-0.30	7.43E-02	Un-changed	
OsAffx.31409.1.S1_s_at	6hr	93.3	337.1	-2.10	2.19E-04	213.2	528.8	-1.40	2.19E-04	Down-regulated	ER6 protein
LOC_Os03g19270	24hr	307.2	520.9	-0.60	1.14E-03	735.5	666.3	0.40	1.49E-02	Un-changed	
OsAffx.3970.1.S1_at	6hr	334.9	2928.6	-3.10	2.00E-05	656.9	4234	-2.60	2.00E-05	Down-regulated	expressed protein
LOC_Os04g31760	24hr	1600.6	1981.1	-0.30	4.00E-05	768.5	2476	-1.60	2.00E-05	Un-changed	
OsAffx.4907.1.S1_x_at	6hr	102.6	193	-1.40	3.07E-04	84	312.7	-1.70	6.80E-05	Down-regulated	xyloglucan endotransglucosylase/hydrolase protein
LOC_Os06g22919	24hr	70.3	193.2	-2.10	2.41E-04	174.8	414.1	-0.60	1.31E-02	Un-changed	21 precursor
OsAffx.7527.1.S1_x_at	6hr	102.9	306.5	-1.60	4.00E-05	136.5	521.5	-2.00	2.00E-05	Down-regulated	retrotransposon protein
LOC_Os12g08564	24hr	112.1	418.7	-2.10	2.00E-05	275.1	615.3	-0.90	2.00E-05	Un-changed	
Os.10002.1.S1_at	6hr	180.2	349	-1.40	2.73E-04	526.8	997.7	-0.60	9.66E-04	Un-changed	altronate dehydratase
LOC_Os07g46950	24hr	328.5	755.3	-1.00	1.67E-04	644	1158.1	-1.30	3.89E-04	Down-regulated	
Os.10280.1.A1_at	6hr	710.1	537.3	0.20	4.94E-01	505.2	837.9	-0.70	2.00E-05	Un-changed	expressed protein
LOC_Os06g41110	24hr	626.7	3061.2	-2.00	2.00E-05	1296.8	2729.8	-1.00	2.00E-05	Down-regulated	
Os.10908.1.S1_a_at	6hr	240.5	177.6	0.80	3.70E-03	171.2	518.4	-1.90	2.00E-05	Un-changed	alpha-amylase isozyme 3D precursor
LOC_Os08g36910	24hr	3.5	275.2	-4.60	2.00E-05	109.7	576.1	-2.50	2.00E-05	Down-regulated	
Os.11164.1.S1_at	6hr	6403.1	7922.4	-0.20	8.38E-02	4535.3	10247.2	-1.00	2.00E-05	Un-changed	pathogenesis-related protein 1 precursor

Probe Set ID/Locus ID	Time	Quinclorac vs. Mock Repeat 1				Quinclorac vs. Mock Repeat 2				Assignment	Description
		Quinclorac	Mock	Log Ratio	P-value	Quinclorac	Mock	Log Ratio	P-value		
LOC_Os07g03458	24hr	3651.1	13447.8	-1.90	2.00E-05	1302.1	10706.9	-2.60	2.00E-05	Down-regulated	
Os.12452.1.S1_s_at	6hr	1046.6	539.9	0.80	2.00E-05	744.6	3182.2	-2.00	2.00E-05	Un-changed	alpha-amylase isozyme 3D precursor
LOC_Os08g36910	24hr	2.3	1027.2	-8.40	2.00E-05	184.1	2768.8	-3.80	2.00E-05	Down-regulated	
Os.13862.1.S1_at	6hr	920.6	1449.7	-0.60	4.00E-05	851.5	1074.7	-0.40	7.12E-03	Un-changed	nuclear ribonuclease Z
LOC_Os09g30466	24hr	370.7	930	-1.30	2.00E-05	418.2	999.6	-1.10	2.00E-05	Down-regulated	
Os.14423.1.S1_at	6hr	411	367.3	0.40	2.03E-03	399	340.1	0.30	2.04E-01	Un-changed	threonine endopeptidase
LOC_Os06g11400	24hr	84.4	198.6	-1.40	2.30E-05	63.8	261.8	-2.10	5.20E-05	Down-regulated	
Os.14849.1.S1_a_at	6hr	6522.9	7646	-0.30	3.36E-03	5989.8	9432.4	-0.60	2.00E-05	Un-changed	nodulin-like protein
LOC_Os06g49500	24hr	1403	3684.8	-1.40	2.00E-05	1744.3	3715.4	-1.10	2.00E-05	Down-regulated	
Os.15007.1.S1_at	6hr	1484.9	1787.2	-0.30	8.51E-03	1652	2028.8	-0.20	1.86E-01	Un-changed	cDNA clone:J013152E19, full insert sequence
	24hr	218.3	662	-1.30	2.30E-05	396.1	886.7	-1.10	2.00E-05	Down-regulated	
Os.15198.1.S1_at	6hr	2003.4	2475.9	-0.50	3.00E-05	2090.9	2434.9	-0.30	2.70E-05	Un-changed	B-box zinc finger family protein
LOC_Os01g10580	24hr	367.6	664.3	-1.10	6.18E-04	294.5	692.9	-1.60	2.00E-05	Down-regulated	
Os.15942.1.S1_s_at	6hr	12561.1	7550	0.80	2.00E-05	11249.7	8394.2	0.40	2.00E-05	Un-changed	ABA/WDS induced protein
LOC_Os04g34600	24hr	1321.3	3313.5	-1.10	2.00E-05	2231	3898.2	-1.10	2.00E-05	Down-regulated	
Os.17502.1.S1_at	6hr	3018.8	3694.7	-0.40	6.92E-04	3086.5	3958.2	-0.40	1.89E-04	Un-changed	transcription-repair coupling factor
LOC_Os11g32880	24hr	231.9	682.9	-1.60	2.00E-05	384.2	814	-1.20	2.00E-05	Down-regulated	
Os.19031.1.A1_at	6hr	599.9	680.6	-0.30	3.36E-03	533.2	769.4	-0.60	2.30E-05	Un-changed	senescence-induced receptor-like serine/threonine-protein kinase precursor
LOC_Os09g18360	24hr	273.1	563.3	-1.10	2.00E-05	72.4	548.4	-2.60	2.00E-05	Down-regulated	
Os.19584.1.A1_at	6hr	154	242.7	-1.10	1.14E-04	188.4	402.5	-0.90	8.90E-05	Un-changed	serine/threonine kinase-like protein
LOC_Os07g35390	24hr	144.6	380.3	-1.00	2.73E-04	91.2	148.8	-1.00	4.00E-05	Down-regulated	
Os.22277.1.S1_at	6hr	4735.6	7818.5	-0.70	2.00E-05	3039.8	7387.3	-1.40	2.00E-05	Un-changed	isocitrate lyase
LOC_Os07g34520	24hr	6873.3	16192.9	-1.20	2.00E-05	7532.8	15051.2	-1.00	2.00E-05	Down-regulated	
Os.2321.1.S1_at	6hr	432.3	689.1	-0.60	5.20E-05	602.2	1301.6	-1.10	2.30E-05	Un-changed	germin-like protein subfamily 1 member 11 precursor
LOC_Os08g09060	24hr	578.1	1124	-1.10	2.00E-05	408.8	1269.6	-1.40	2.00E-05	Down-regulated	
Os.24834.1.S1_at	6hr	4248.8	6219.8	-0.50	2.00E-05	4847.7	7034.1	-0.50	3.00E-05	Un-changed	monooxygenase/ oxidoreductase
LOC_Os09g37620	24hr	333.8	1021.3	-1.40	1.67E-04	629.3	1255.4	-1.10	1.67E-04	Down-regulated	
Os.25556.1.S1_at	6hr	4362.1	8166.1	-0.90	3.00E-05	5171.9	9205	-0.80	2.00E-05	Un-changed	transposon protein, CACTA, En/Spm sub-class
LOC_Os08g28350	24hr	2588.6	6085	-1.20	2.00E-05	2860.4	6116.2	-1.10	2.00E-05	Down-regulated	
Os.26569.1.S1_at	6hr	1151.3	2284.4	-0.90	2.00E-05	1181	3569.4	-1.60	2.00E-05	Un-changed	caffeic acid 3-O-methyltransferase
LOC_Os04g09604	24hr	949.5	1838.4	-1.00	2.00E-05	621.9	1827.6	-1.30	2.00E-05	Down-regulated	
Os.27811.1.S1_at	6hr	8768	13220.2	-0.60	2.00E-05	7651.4	12662.4	-0.80	2.00E-05	Un-changed	expressed protein
LOC_Os09g17329	24hr	2773.4	9065.7	-1.60	2.00E-05	2115.8	6289.3	-1.60	2.00E-05	Down-regulated	
Os.27864.1.S1_at	6hr	7257.4	11574.3	-0.70	2.00E-05	6508.1	11065.8	-0.80	2.00E-05	Un-changed	peroxidase 12 precursor
LOC_Os04g59200	24hr	1891.6	6734.4	-2.10	2.00E-05	928.7	4393.6	-2.50	2.00E-05	Down-regulated	
Os.29979.1.S1_at	6hr	477.2	547.4	-0.10	5.00E-01	427.1	650.2	-0.60	5.20E-05	Un-changed	OsWRKY10 - Superfamily of rice TFs having WRKY and zinc finger domains
LOC_Os01g09100	24hr	131.3	458.7	-1.70	6.00E-05	194	489.1	-1.30	2.30E-05	Down-regulated	
Os.30049.1.S1_at	6hr	6886.5	6480.6	0.10	5.00E-01	6871.4	6409.6	0.10	5.00E-01	Un-changed	ribonucleoprotein A, chloroplast precursor
LOC_Os03g25960	24hr	2244.7	5369.3	-1.10	2.00E-05	2479.9	5414.9	-1.10	2.00E-05	Down-regulated	
Os.31171.1.S1_at	6hr	1858.8	1055.7	0.60	4.00E-05	2501.1	1286.2	0.70	1.47E-04	Un-changed	expressed protein
LOC_Os01g40290	24hr	53	1129.4	-4.10	2.00E-05	120.5	786.3	-2.30	2.00E-05	Down-regulated	
Os.32482.2.S1_x_at	6hr	3152	2155.5	0.60	2.14E-04	1725.3	1820	-0.10	5.00E-01	Un-changed	expressed protein
LOC_Os01g59090	24hr	636.8	1837.8	-1.40	2.00E-05	474.3	1289.6	-1.00	2.00E-05	Down-regulated	
Os.33704.3.S1_x_at	6hr	1342	1727.5	-0.30	9.29E-03	1239.8	1672.3	-0.20	4.41E-02	Un-changed	expressed protein
LOC_Os01g20110	24hr	395.9	832.2	-1.10	2.00E-05	511.1	1107.1	-1.20	2.00E-05	Down-regulated	
Os.33770.1.S1_s_at	6hr	2035.2	1806.8	0.10	5.00E-01	1572.3	1546.5	0.10	5.00E-01	Un-changed	RNA binding protein
LOC_Os01g59510	24hr	386.9	913.8	-1.40	2.00E-05	405.6	949.7	-1.20	2.00E-05	Down-regulated	
Os.3386.1.S1_x_at	6hr	300	223.6	0.60	5.52E-04	244.9	248.2	-0.10	5.00E-01	Un-changed	anthocyanin regulatory C1 protein
LOC_Os06g10350	24hr	164.8	409.5	-1.30	1.89E-04	258.2	495.8	-1.00	1.47E-04	Down-regulated	
Os.34496.1.S1_at	6hr	4246.2	2702.9	0.70	3.50E-05	1198.5	2576.8	-1.00	2.00E-05	Un-changed	PGPS/D12
LOC_Os03g61500	24hr	60.1	1067.7	-3.90	2.00E-05	7.7	565.4	-5.70	2.00E-05	Down-regulated	
Os.34624.2.S1_s_at	6hr	3294.2	3132.5	0.00	5.00E-01	3704	3761.1	0.00	5.00E-01	Un-changed	glutamate formiminotransferase
LOC_Os03g38540	24hr	189	433.5	-1.20	6.00E-05	223.2	516.5	-1.10	2.00E-05	Down-regulated	

Probe Set ID/Locus ID	Time	Quinclorac vs. Mock Repeat 1				Quinclorac vs. Mock Repeat 2				Assignment	Description
		Quinclorac	Mock	Log Ratio	P-value	Quinclorac	Mock	Log Ratio	P-value		
Os.37247.1.S1_at	6hr	2568.1	1764.6	0.50	5.52E-04	2126.3	1787.6	0.20	2.12E-02	Un-changed	elongation factor P
LOC_Os12g02380	24hr	1184.9	2460	-1.00	2.00E-05	1150.7	2776.7	-1.20	2.00E-05	Down-regulated	
Os.37644.1.S1_at	6hr	1746.8	728.2	0.90	2.00E-05	2279.7	1014.9	1.00	2.00E-05	Un-changed	cDNA clone:002-134-B03, full insert sequence.
	24hr	29.5	228.5	-3.00	1.14E-04	45	197.6	-2.00	2.41E-04	Down-regulated	
Os.38638.1.S1_at	6hr	55.8	222.5	-1.60	4.00E-05	127.3	143.1	-0.40	2.74E-01	Un-changed	early nodulin 93
LOC_Os06g04990	24hr	133.2	362.3	-1.50	1.01E-04	113.7	268.5	-1.20	4.07E-03	Down-regulated	
Os.40210.1.S1_at	6hr	344.9	406.8	-0.50	1.65E-03	355.3	333.6	0.10	5.00E-01	Un-changed	KHG/KDPG aldolase
LOC_Os07g12160	24hr	140.6	314.4	-1.10	2.00E-05	127.4	462.7	-1.80	2.00E-05	Down-regulated	
Os.41106.1.S1_at	6hr	116.4	148.7	-0.50	6.56E-02	137.9	137	0.00	5.00E-01	Un-changed	xylanase inhibitor protein 1 precursor
LOC_Os07g43820	24hr	335.8	966	-1.40	2.00E-05	360.7	965.4	-1.10	4.60E-05	Down-regulated	
Os.4618.1.S1_at	6hr	7996.6	3469.6	1.20	2.00E-05	8263.9	6101.4	0.40	2.30E-05	Un-changed	expressed protein
LOC_Os01g32670	24hr	386	3302.9	-3.10	2.00E-05	397.4	1555.2	-1.80	2.00E-05	Down-regulated	
Os.46576.1.S1_at	6hr	141.5	250.1	-0.70	8.38E-02	92	285.5	-1.40	8.65E-04	Un-changed	nonspecific lipid-transfer protein precursor
LOC_Os10g36100	24hr	168.4	530.6	-2.20	2.00E-05	263.8	538.2	-1.00	4.00E-05	Down-regulated	
Os.46576.1.S1_x_at	6hr	236	218.1	0.00	5.00E-01	38.3	210.1	-2.10	6.98E-02	Un-changed	nonspecific lipid-transfer protein precursor
LOC_Os10g36100	24hr	192.7	408.9	-1.00	1.47E-04	172.3	448.8	-1.10	1.49E-03	Down-regulated	
Os.4770.1.S1_a_at	6hr	353.8	374.6	-0.10	5.00E-01	310	267.1	0.30	4.29E-01	Un-changed	tumor-related protein
LOC_Os01g06560	24hr	411.1	948.5	-1.50	2.00E-05	472.1	817.8	-1.00	3.50E-05	Down-regulated	
Os.4844.1.S1_at	6hr	302	465.1	-0.70	1.89E-04	281.1	638.3	-1.20	2.70E-05	Un-changed	peroxidase 1 precursor
LOC_Os05g06970	24hr	292.2	833.5	-1.40	2.00E-05	316.6	516.8	-1.00	2.00E-05	Down-regulated	
Os.49191.1.S1_at	6hr	1026.6	1360.8	-0.40	1.01E-04	1295.6	1294.6	0.00	5.00E-01	Un-changed	expressed protein
LOC_Os09g21460	24hr	618.4	1696.7	-1.40	2.00E-05	781.3	2129.4	-1.40	2.00E-05	Down-regulated	
Os.49874.2.S1_x_at	6hr	3499.5	3050.9	0.10	5.00E-01	3590.1	2997.6	0.30	1.20E-03	Un-changed	chaperonin, chloroplast precursor
LOC_Os02g54060	24hr	1059.2	2303.2	-1.00	3.07E-04	908.4	3270.8	-1.70	2.30E-05	Down-regulated	
Os.50410.1.S1_x_at	6hr	892	800	0.00	5.00E-01	461.9	514.9	-0.40	1.86E-01	Un-changed	antiporter/ drug transporter/ transporter
LOC_Os04g59600	24hr	429.3	902.6	-1.20	4.60E-05	316.4	766.1	-1.30	2.00E-05	Down-regulated	
Os.52159.1.S1_at	6hr	542.2	516.3	-0.20	1.53E-01	626.4	564.7	0.00	5.00E-01	Un-changed	FAD dependent oxidoreductase
LOC_Os05g34040	24hr	269.7	448.9	-1.00	2.00E-05	271.9	699.4	-1.30	6.00E-05	Down-regulated	
Os.53912.1.S1_x_at	6hr	7727.8	3553.5	1.00	3.00E-05	5646.7	3659.8	0.50	5.52E-04	Un-changed	ABA/WDS induced protein
LOC_Os04g34600	24hr	598.2	1516.2	-1.30	5.20E-05	680.1	1561.6	-1.10	2.49E-03	Down-regulated	
Os.54421.1.S1_at	6hr	301	200.6	0.30	5.00E-01	291.9	108.1	0.30	5.00E-01	Un-changed	cytochrome P450 94A2
LOC_Os05g37250	24hr	154.8	298.8	-1.10	4.00E-05	103.6	259.3	-1.20	2.00E-05	Down-regulated	
Os.57400.1.S1_at	6hr	342.1	319.4	-0.10	5.00E-01	295.7	278.3	0.00	5.00E-01	Un-changed	conserved hypothetical protein
LOC_Os04g54380	24hr	169	439.9	-1.10	2.30E-05	153.7	393.7	-1.60	2.00E-05	Down-regulated	
Os.5754.1.S1_at	6hr	6429.1	8114.3	-0.50	2.00E-05	6186.6	7974.4	-0.50	1.14E-04	Un-changed	DAG protein, chloroplast precursor
LOC_Os08g04450	24hr	1143.8	2626.1	-1.20	2.00E-05	1223.9	2896	-1.30	2.00E-05	Down-regulated	
Os.7004.1.S1_at	6hr	1590.2	788.3	0.90	7.80E-05	871.6	536.3	0.60	1.01E-04	Un-changed	expressed protein
LOC_Os12g13340	24hr	911.3	2091	-1.20	2.00E-05	709.7	3571.4	-2.30	2.00E-05	Down-regulated	
Os.7278.1.S1_x_at	6hr	3068.6	4984.7	-0.70	2.00E-05	2363.6	6933.8	-1.30	2.00E-05	Un-changed	peptidase/ subtilase
LOC_Os04g03796	24hr	2706.5	5821.8	-1.10	2.70E-05	1111.6	4045.8	-1.90	2.00E-05	Down-regulated	
Os.7756.2.S1_at	6hr	8251.7	9986.4	-0.40	1.67E-04	9119.1	13180.2	-0.60	2.00E-05	Un-changed	ripening-related protein-like
LOC_Os10g36500	24hr	1459.4	3768.1	-1.40	2.00E-05	1493.9	4315.8	-1.50	2.00E-05	Down-regulated	
Os.7756.2.S1_x_at	6hr	8144.7	9618.1	-0.30	2.30E-05	8175.4	12153.5	-0.70	2.00E-05	Un-changed	ripening-related protein-like
LOC_Os10g36500	24hr	1144.7	3608.1	-1.40	2.00E-05	1330.6	3567.9	-1.60	2.00E-05	Down-regulated	
Os.7944.1.S1_at	6hr	2198.6	1868.3	0.20	5.00E-01	1839.8	2204.5	-0.30	9.99E-02	Un-changed	cDNA clone:J023011I05, full insert sequence.
	24hr	114.5	220.3	-1.00	1.47E-04	141	274.9	-1.10	3.50E-05	Down-regulated	
OsAffx.12379.1.S1_at	6hr	3028.7	4949.4	-0.60	2.70E-05	3120.5	5324.5	-0.80	2.00E-05	Un-changed	oxygen evolving enhancer protein 3
LOC_Os02g36850	24hr	379.7	1306.9	-1.40	1.89E-04	541.8	1279.4	-1.20	5.20E-05	Down-regulated	
OsAffx.12954.1.S1_at	6hr	272.4	101.6	1.60	8.65E-04	144.4	106.7	0.70	1.20E-03	Un-changed	embryonic protein DC-8
LOC_Os03g20680	24hr	106.4	505.3	-2.40	2.70E-05	129.3	807.4	-2.30	4.00E-05	Down-regulated	
OsAffx.17468.1.S1_s_at	6hr	127.2	326.9	-1.00	2.00E-05	148.4	326.2	-0.80	2.30E-05	Un-changed	ACR5
LOC_Os08g42080	24hr	49.2	133.5	-1.50	2.14E-04	86	198.1	-1.20	1.30E-04	Down-regulated	
OsAffx.25285.1.S1_at	6hr	15289	16844	-0.20	1.53E-01	17898.3	23557.1	-0.40	2.00E-05	Un-changed	EF hand family protein

Probe Set ID/Locus ID	Time	Quinclorac vs. Mock Repeat 1				Quinclorac vs. Mock Repeat 2				Assignment	Description
		Quinclorac	Mock	Log Ratio	P-value	Quinclorac	Mock	Log Ratio	P-value		
LOC_Os03g29770	24hr	1830.9	6527.5	-1.80	2.00E-05	3395.9	6808.9	-1.10	2.00E-05	Down-regulated	
OsAffx.4648.1.S1_at	6hr	3861.4	4770.9	-0.30	8.90E-05	3114	3903.5	-0.40	6.80E-05	Un-changed	CRS1
LOC_Os05g47850	24hr	792.1	1819	-1.10	2.00E-05	880.4	1993.5	-1.00	7.80E-05	Down-regulated	
OsAffx.7530.1.S1_s_at	6hr	1006.8	633.6	0.80	2.30E-05	826.6	893.9	-0.10	5.00E-01	Un-changed	expressed protein
LOC_Os12g08930	24hr	92.5	234.7	-1.00	4.92E-04	120.2	297.4	-1.00	5.41E-03	Down-regulated	
