# Supplementary material for: Rice transcriptome analysis to identify possible herbicide quinclorac detoxification genes
Source: Front Genet. 2015 Sep 29;6:306. doi: 10.3389/fgene.2015.00306 (PMC4586585; doi:10.3389/fgene.2015.00306)
Supplement: Supplemental Table 2 — List of primer sequences used in real-time RT-PCR. [file Table2.DOC]

**Supplemental Table 2. List of primer sequences used in real-time RT-PCR**

| **Gene Name** | **Forward** | **Reverse** |
| --- | --- | --- |
| **Rice** | | |
| **LOC_Os01g43700** | GTATAGATCCGTGCATCCGG | GGAACATGGAGCTTCCAGTG |
| **LOC_Os01g43710** | TACATCCACGCACCATTCAC | GCAAGTGAGCAGCATGGTTA |
| **LOC_Os03g55240** | GCTAGCAATCGCTCTTTGAGA | GAAATTGAAATACCTGGCGTGT |
| **LOC_Os07g23570** | CCTAAGGCTGTACAGCCCTG | ATCGGGATCGTCAATAGTGC |
| **LOC_Os07g44140** | TATCGATTCCGATTGCAACA | TATTTTCCCGCCTTTGACAC |
| **LOC_Os01g55940** | GGGGTAAGCTGGTAACGTCA | TTCACTCCCATTTTTGCACA |
| **LOC_Os09g20220** | GCCAAGACGCTTACTGATCC | GCGATTGTTAACCAAACCAAA |
| **LOC_Os09g34250** | CAGCTCCGACAAAAATATCG | GACGAATCCACCTGTCTCAA |
| **LOC_Os06g11290** | CGAGGAGGTTTGAGCTTGAC | TGACTCCTCATCAATCTCCTCA |
| **Arabidopsis** | | |
| **At3g28740** | TTTTGAGTGGGAGAGGGTTG | TGCAACGATGTAGCTTTTCG |
| **At4g37370** | TTGGAATGGGAGAAGATTGG | ACAACAGAGGGACGTGCTCT |
| **At3g14620** | GGGGACCAAGATTCTGTCCT | TGCGTGAGTGTACGAAGGAG |
| **At1g59500** | GCTGTTTGGAGATGGAGGAA | CACCACACGTATCTCCAACG |
| **At1g05680** | GCAGAAGGTGATGGGTTTGT | TTCCCTTTCTCTCCCTCCAT |
| **At1g05560** | GGAAAACGCAAAGAAATGGA | CTCCACAAAAGCCTCCATGT |
